# Public health impact of catch-up vaccination or additional booster doses with pre-erythrocytic malaria vaccine R21/Matrix-M: a modelling study

**DOI:** 10.1186/s12916-026-04822-y

**Published:** 2026-03-25

**Authors:** Kelly McCain, Hillary M. Topazian, Joseph D. Challenger, Lucy Okell, Peter Winskill, Azra C. Ghani

**Affiliations:** 1 MRC Centre for Global Infectious Disease Analysis, School of Public Health, Imperial College London, London, UK; 2 Saw Swee Hock School of Public Health, National University of Singapore and National University Health System, Singapore, Singapore; 3 Lee Kong Chian School of Medicine, Nanyang Technological University, Singapore, Singapore

**Keywords:** Scenario modelling, Malaria vaccine, Malaria transmission model, School-aged children, Catch-up vaccination, Malaria control

## Abstract

**Background:**

The malaria vaccine R21/Matrix-M is recommended for young children in malaria-endemic regions. However, the small vaccine-eligible population and waning vaccine efficacy mean that routine vaccination is unlikely to prevent severe cases in older children who experience significant malaria burden. As R21/Matrix-M vaccination expands, targeting older age groups may be warranted, depending on funding.

**Methods:**

Using a stochastic, individual-based *Plasmodium falciparum* malaria transmission model, we estimate the impact of (1) one-off catch-up campaigns with R21/Matrix-M to previously unvaccinated age groups between age 6 months and 14 years, and/or (2) extra boosters at 2, 5, and/or 10 years after the primary series in low, moderate, and high transmission settings. We assume that vaccine immunogenicity in older children is equivalent to that of the standard target age group, though clinical trials have shown lower immunogenicity in older children.

**Results:**

Catch-up campaigns in moderate-to-high transmission settings targeting younger children averted the most uncomplicated cases per 1000 additional doses (358 (95% credible interval (CI) 113–570) in children aged 6 months–2 years at 45% *Pf*PR_2-10_), compared with targeting older children. In low transmission settings, the impact was similar across age groups, with a slightly higher impact when targeting school-aged children (373 (95% CrI 240–518) in children aged 5–9 years at 5% *Pf*PR_2-10_). Across extra booster strategies, an extra booster 10 years post- primary series averted the most severe cases per 1000 additional doses at low transmission (12 (95% CrI 6–18) at 5% *Pf*PR_2-10_), but the least at high transmission (− 4 (95% CrI − 11–3) at 45% *Pf*PR_2-10_).

Expanding the vaccine-eligible population in areas of moderate-to-high transmission often had higher incremental efficiency than routine age-based vaccination at low transmission. For example, an extra booster 5 years post-primary series averted 835 (95% CrI 605-1274) clinical cases per 1000 additional doses in a 45% *Pf*PR_2-10_ perennial setting versus 247 (95% CrI 177-345) clinical cases per 1000 doses with routine vaccination in a 5% *Pf*PR_2-10_ perennial setting. Sensitivity analyses assuming lower immunogenicity in older children modestly reduced the per-dose impact, but overall conclusions remained unchanged.

**Conclusions:**

Catch-up campaigns or extra booster doses of R21/Matrix-M can provide benefits beyond routine administration, with the per-additional-dose value approaching that of routine vaccination, but this varies by transmission and seasonality setting. Further empirical studies, particularly on vaccine efficacy in older children, are warranted to inform policy guidance for malaria vaccination implementation.

**Supplementary Information:**

The online version contains supplementary material available at 10.1186/s12916-026-04822-y.

## Background

Malaria is a major contributor to morbidity and mortality globally, with an estimated 263 million cases in 2023, of which 94% of cases and 95% of deaths were in sub-Saharan Africa (SSA) [1]. Two malaria vaccines, RTS,S/AS01 and R21/Matrix-M, have been recommended by the World Health Organization (WHO) for use in young children in SSA in 2021 and 2023, respectively, prioritising areas with moderate to high malaria transmission [2, 3]. Both vaccines induce anti-circumsporozoite protein (CSP) antibodies, which act during the pre-erythrocytic stage of infection when sporozoites are inoculated from an infectious bite from an *Anopheles* mosquito.

A phase III trial of RTS,S/AS01 demonstrated 46% (95% CI 42–50%) vaccine efficacy among children receiving 4 doses over 18 months with age-based implementation (3 monthly doses with a booster dose 12 months post-dose 3) [4], declining to 36.3% (95% CI 31.8–40.5%) over 4 years [5]. However, efficacy for the combination compared to SMC alone was maintained over 5 years (57.7% [95% CI 53.3–61.7%]) with seasonal implementation (3 monthly doses delivered before the high transmission season) alongside annual booster doses and seasonal malaria chemoprevention (SMC) [6]. The phase III trial for R21/Matrix-M in 2021–2022 evaluated age-based and seasonal vaccination of children aged 5–36 months. Vaccine efficacy against clinical malaria after 1 year was 75% (95% CI 71–78%) and 67% (95% CI 59–73%) for seasonal and age-based implementation, respectively [7]. Both efficacy against clinical malaria and antibody titres after 12 months were higher in children aged 5–17 months at the time of vaccination compared to children aged 17–36 months [7].

Accordingly, for both vaccines, WHO currently recommends a four-dose schedule delivered either through age-based (implemented by 23 out of the 25 countries who have introduced malaria vaccination as of March 2026), seasonal (no countries have implemented this yet), or hybrid delivery strategies (implemented by 2 out of the 25 countries), in which the primary series is delivered year round to children from 5 months of age as under age-based delivery and with up to 2 booster doses delivered annually before the start of the high transmission season [2, 8]. A fifth dose 1 year after the fourth dose may be considered in seasonal settings.

Protection from both vaccines is likely to wane over time. Longer-term follow-up data for R21/Matrix-M are not yet available, although clinical efficacy was maintained up to 2 years following the fourth dose [7]. However, in the phase III trial of the similar vaccine RTS,S/AS01, vaccine efficacy against clinical malaria among children aged 5–17 months was 50.4% (95% CI 45.8–54.6) after 14 months [9], compared to 36.3% (95% CI 31.8–40.5) over 4 years of follow-up [5]. After protection from an imperfect intervention wanes, rebound or delayed malaria cases among the previously protected population are often observed due to slower natural immunity acquisition than in an unprotected population [10]. Delayed malaria may result in negative vaccine efficacy at older ages when a vaccinated cohort with waning vaccine-induced immunity and low natural immunity experiences more cases of malaria relative to an unvaccinated cohort with high levels of natural immunity [11]. Over 7 years of follow-up of the RTS,S/AS01 vaccine trial in 2 sites, vaccine efficacy waned to 4.4% (− 14.5–24.6) and showed negative efficacy against clinical malaria among highly exposed children in the fifth year post-vaccination [11]. However, the benefit of the protective intervention often outweighs the negative rebound effects [12, 13]. Additional booster doses given before vaccine-induced immunity wanes could prevent or delay a rebound of clinical malaria [14].

In sub-Saharan Africa, 76% of deaths from malaria are in children under 5 years old, with the highest relative burden among young children in moderate to high transmission settings [1]. However, 20-40% of clinical cases of malaria are in school-aged children (aged 5–15 years), with the proportional burden of cases in this age group peaking in moderate transmission sites [15]. Although school-aged children have a lower risk of symptomatic malaria infection compared to children under 5, they contribute disproportionately to the infectious reservoir as they are not typically targeted for seasonal malaria chemoprevention (SMC), sleep less frequently under bed nets, and are often the age group with the highest parasite prevalence [16–19]. In low transmission settings, the risk of severe malaria is similar across all age groups [20]. Furthermore, the small proportion of the current vaccine-eligible population, combined with waning efficacy against clinical and severe disease from a vaccine, means that routine administration to young children is unlikely to generate indirect protection (i.e. herd immunity) for older children and adults [21]. Expanding vaccination to older age groups will provide both direct protection to a larger proportion of the population and may also provide indirect protection through preventing infection across a larger proportion of the infectious reservoir [22].

Mathematical modelling has previously been used to estimate the public health impact of malaria vaccine introduction across SSA for both the RTS,S/AS01 and R21/Matrix-M vaccines [23–25]. Here, we extend the work presented in Schmit et al. for the R21/Matrix-M vaccine [24] to estimate the benefit in terms of clinical and severe cases averted of extending vaccination through catch-up campaigns to children up to age 14 years and/or via additional boosting to older children at combinations of 2, 5, and/or 10 years after the primary series. Results from this study can inform ongoing discussions about wider use cases for the R21/Matrix-M malaria vaccine.

## Methods

### Modelling

We used a stochastic individual-based model of *Plasmodium falciparum* malaria developed at Imperial College London [26–28] using the open-source *malariasimulation* R package (v 2.0.1) [29]*.* We estimated the impact of (1) a catch-up campaign expanding the population eligible for the initial 4-dose R21/Matrix-M vaccination to a range of age groups between 6 months and 14 years; (2) adding extra boosters at 2, 5, and/or 10 years after third dose of routine age-based vaccination (beyond the standard 1-year booster); and (3) the combination of (1) and (2)*.* The model incorporates transmission dynamics between the mosquito vectors and the human hosts and a range of malaria control interventions, including pre-erythrocytic vaccines. A detailed description of the model can be found in Additional file 1 and is summarised in brief below.

After age-dependent exposure to an infected bite, individuals may become infected and, following a latent period, develop clinical disease or asymptomatic infection. A proportion of those with clinical disease are successfully treated. Treated individuals recover at a specified rate and return to the susceptible state, while those with unsuccessful treatment or no treatment move first to the asymptomatic state, then to a sub-patent infection state as the parasite load falls below the microscopy-detectable limit before the individual returns to the susceptible state. The model incorporates maternally acquired immunity against clinical disease, which decays over the first few months of life, and pre-erythrocytic and blood stage immunity, each modelled to increase as a function of prior exposure, which generally increases with age. The mosquito vectors are modelled using a deterministic compartmental structure, following the female mosquito through its life stages (egg, larval, pupal, adult), with infection of adult females occurring from biting an infected human. The model diagram and transition rates between human infection states are in Additional file 1: Fig. S1 and Table S1 [3, 7, 15, 23, 24, 28, 30–45].

Modelled anti-CSP antibody titres and the relationship of antibody titre with clinical protection were previously fit to immunogenicity and case data from the phase II trial for R21/Matrix-M [24, 35]. In this analysis, we modelled the R21/Matrix-M vaccine in *malariasimulation* using the median values of the derived antibody titre and vaccine efficacy parameters (Additional file 1: Table S2) [24]. We assume vaccine efficacy in the model to be zero until after the third dose. Antibody dynamics are described by a biphasic exponential decay model that allows short- and long-term decays of anti-CSP antibodies.

The results presented in the main text assume the same immunogenicity and vaccine efficacy regardless of age at vaccination, while in Additional file 1 (pp. 39 to 51), we show a sensitivity analysis assuming the peak antibody titre for age at vaccination of 5 years or older has a geometric mean ratio (GMR) of 0.64 compared to the parameters fitted to data on young children, based on a study comparing the immunogenicity of RTS,S/AS02, a pre-erythrocytic vaccine similar to R21/Matrix-M, in children aged 6 to 11 years and 1 to 5 years [40], and assuming a lower GMR of 0.4 compared to the fitted values. Under both scaling assumptions, we assumed that the relationship between antibody titre and vaccine efficacy remains unchanged regardless of the age group vaccinated.

### Model scenarios

We assumed that baseline prevalence (via microscopy) of *P. falciparum* in children aged 2–10 years (*Pf*PR_2–10_) at the beginning of the simulation incorporated existing usage of malaria control interventions (such as insecticide-treated bed nets, seasonal malaria chemoprevention, indoor residual spraying), so these interventions were not modelled explicitly. Scenarios were modelled for seasonal and perennial transmission settings (Additional file 1: Table S3) across a range of prevalence settings (*Pf*PR_2–10_ ranging 1–65%). Each additional vaccine intervention (expanded age groups or extra booster doses) was supplemental to routine age-based vaccination. We did not explicitly model the impact of expanded age groups or booster doses added to a routine seasonal vaccination strategy (we only modelled expanded age groups or extra booster doses added to a routine age-based strategy) as the overall impact of seasonal versus age-based delivery strategies for R21/Matrix-M in our model is similar (Additional file 1: Fig. S2). None of the countries currently implementing pre-erythrocytic malaria vaccination has chosen to use a seasonal routine implementation strategy, though Mali and Guinea-Bissau have introduced hybrid vaccination [8]. We modelled a population of 300,000 individuals of all ages with fixed treatment coverage of artemether-lumefantrine to 45% among clinical malaria cases (Additional file 1: Table S3) [23]. Simulations were run with a burn-in period of 20 years, following which the vaccine was introduced. Routine vaccination was continually implemented from this time onwards, while catch-up campaigns were assumed to be delivered at a single timestep (see below). To capture the impact of extra booster doses given 10 years after the primary series and any effects of delayed malaria, the simulations were run for 30 years after initial vaccination.

Vaccination strategies are summarised in Table 1. Age-based vaccination was implemented with the primary series of 3 doses starting at 6 months of age and a booster dose at 12 months post-primary series, resulting in vaccine doses at 6, 7, 8, and 20 months of age. We also evaluated one, two, or three additional booster doses at combinations of 2, 5, and/or 10 years after the primary series. Catch-up vaccination to a range of age groups was modelled as a single targeted campaign of the primary series and the booster dose 12 months later and was supplementary to ongoing routine age-based vaccination. The catch-up vaccination campaign was delivered at the time of routine age-based vaccine introduction in perennial settings and at 5.5 months before the peak clinical incidence in the year of vaccine introduction in seasonal settings. Vaccination coverage for all scenarios was assumed to be 80% for the primary series, and 80% of those who received the primary series received any following booster dose (64% of the target population), similar to coverage achieved during the Malaria Vaccine Implementation Programme [3] (Additional file 1: Table S2).

**Table 1:** Summary of modelled vaccination strategies. All children receiving the vaccine through a catch-up campaign were previously unvaccinated against malaria. Age groups are inclusive (e.g. a catch-up campaign to children 6 months to 2 years old would include children up to 3 years minus 1 day old)

Additional vaccine strategy	Background vaccination	Additional boosters	Age groups targeted for catch-up campaign (inclusive)
No vaccination	–	–	–
Routine age-based only	Primary series of three doses + single booster 12 months post-dose 3 to children from 6 months of age	–	–
Catch-up		–	5–9 years 5–14 years 6 months–2 years 6 months–4 years 6 months–9 years 6 months–14 years
Additional boosters		2*,* 5, 10, 2 and 5, 2 and 10, 5 and 10, or 2, 5, and 10 years post-primary series	–
Catch-up + additional boosters		2*,* 5, 10, 2 and 5, 2 and 10, 5 and 10, or 2, 5, and 10 years post-primary series	5–9 years 5–14 years 6 months–2 years 6 months–4 years 6 months–9 years 6 months–14 years

Primary model outcomes were clinical and severe cases averted per 1000 people and per 1000 doses compared to a baseline of no vaccination, and clinical and severe cases averted per 1000 additional doses delivered compared to a counterfactual of routine age-based vaccination. Clinical and severe cases and doses delivered were summed over the 30 years after the introduction of vaccination. Estimates are represented as the median, and 2.5% and 97.5% percentiles of 50 parameter draws of the posterior distribution of the main malaria transmission model for each scenario.

### Cohort analysis

We followed cohorts of children in the routine age-based and extra booster strategies initiated every 6 months until the end of the 30-year simulation. For example, a cohort of children vaccinated in the first 6 months of the simulation was followed for 30 years, while children vaccinated in the first 6 months of the 25th year were followed for 5 years. For no vaccination, routine age-based, and extra booster scenarios, we averaged outcomes by 1-year age group (except for age 6 months to 1 year) across 60 cohorts (one cohort per half-year for the 30-year simulation). For catch-up scenarios, a single cohort of vaccinated children was followed throughout the simulation (e.g. for the scenario assuming a catch-up campaign to children aged 6 months to 4 years, all children in this age group at the beginning of the simulation were followed for 30 years). Outcomes averted were calculated by comparing the number of clinical and severe cases per age group in the vaccinated cohort and a baseline cohort (unvaccinated or routine age-based vaccination) in the same half-year. We report the annual medians across 50 stochastic draws of the posterior distributions of the main malaria transmission model for each scenario.

### Incremental efficiency frontier

An efficiency frontier compares the benefits of an intervention against its cost [43]. We used the additional number of R21/Matrix-M doses delivered per person as a proxy for cost and the total additional clinical or severe cases averted per 1000 people as proxies for benefit, both compared to the routine age-based strategy over the 30-year simulation and assuming a proportional increase in benefit. We calculated incremental cost-effectiveness ratios (ICER) using the *dampack* package (v 1.0.1) in R and plotted the efficiency frontier for each transmission and seasonality setting. Non-dominated scenarios fell on the efficiency frontier, and strongly dominated scenarios, those with fewer clinical or severe cases averted and higher costs, were removed. Weakly dominated scenarios, in which a combination of two other strategies could provide a higher benefit with fewer doses, were kept in the plots, but did not lie on the efficiency frontier. We also conducted a sensitivity analysis by calculating the expected loss for each strategy, details of which, along with extended methods for the efficiency frontier analysis, are in Additional file 1 (pp. 47–49).

## Results

### Catch-up strategies

**Fig. 1:**
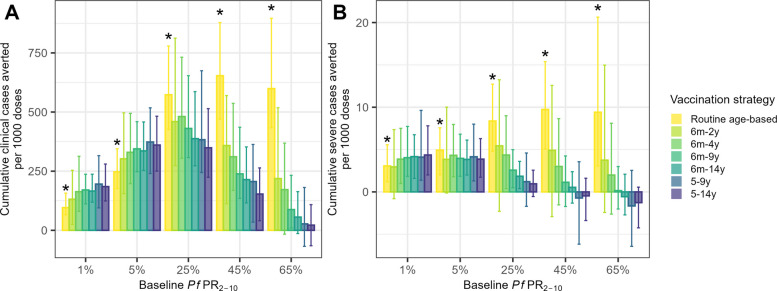
Catch-up campaign impact per 1000 additional doses, perennial setting. Cumulative clinical (**A**) and severe (**B**) cases averted per 1000 doses over a 30-year simulation in a perennial setting. Values for routine age-based vaccination (bars with * above) show the outcome per 1000 doses delivered relative to no vaccination, while all other plotted strategies show additional outcomes averted per additional doses delivered relative to routine age-based vaccination. Each catch-up strategy is supplementary to routine age-based vaccination, which includes a single booster dose at 12 months post-primary series. Additional clinical and severe cases averted, and additional doses are compared to a baseline of routine age-based vaccination on the y-axis. The bars show median values, and the error bars show 95% credible intervals from 50 stochastic model runs. Negative values indicate more clinical or severe cases relative to the baseline of no vaccination. Note that the y-axes in plots **A** and **B** are different

Figure 1 shows the modelled additional cases averted per dose of one-off catch-up campaigns to different age groups delivered at vaccine introduction, compared to routine age-based implementation in a perennial setting. In low transmission settings, a similar number of additional cases are averted per dose across target age groups, with a slight trend to higher impact when targeting school-aged children (from 5 to 9 or 14 years of age) (Fig. 1). As transmission intensity increases, the most efficient age group to target for a catch-up campaign becomes younger: targeting children aged 6 months to 4 years at 25% PfPR2–10 and targeting children 6 months to 2 years at high transmission (45–65% PfPR2–10), avert the most additional cases per additional dose. However, catch-up campaigns in the 65% *Pf*PR_2–10_ transmission setting are each projected to prevent similar numbers of clinical and severe cases per person regardless of target age group, while in all other transmission settings, delivering doses to wider age groups averts more clinical cases per person (Table 2, Additional file 1: Fig. S3). In contrast, in low transmission settings, expanding target age groups increases both clinical cases averted per person (Additional file 1: Fig. S3) and per additional dose (Fig. 1). The number of severe cases averted remains similar across target age groups, though targeting older children marginally increases efficiency (Fig. 1).

**Table 2:** Outcomes averted per 1000 people, per 1000 additional doses, and per 1000 doses in perennial settings. Strategies included in the table are either catch-up vaccination or additional boosters, but not a combination of catch-up and additional boosters, and are summarised over the total population and a 30-year time horizon. Clinical and severe cases averted per 1000 people and per 1000 doses are compared to a baseline of no vaccination, while additional clinical and severe cases averted per 1000 additional doses of catch-up or extra booster strategies are compared to a baseline of routine age-based vaccination. Each is supplementary to routine age-based vaccination that includes a single booster dose at 12 months post-primary series. The age groups listed are those targeted for a catch-up vaccination campaign (e.g. 6m-14y refers to a catch-up vaccination campaign to children between 6 months and 14 years at vaccine introduction that is supplementary to continuous routine age-based vaccination). Booster dose timing (e.g. 10y booster) is noted as the timing of additional booster dose(s) (e.g. 10y booster refers to routine age-based vaccination with a single additional booster at 10 years after the primary series, so this strategy has two booster doses, the standard one at 12 months plus the additional one at 10 years). The table is grouped by transmission intensity. Median values with 95% credible intervals of 50 stochastic model runs are presented

Strategy	Clinical cases averted per 1000 people	Severe cases averted per 1000 people	Additional clinical cases averted per 1000 additional doses (relative to routine age-based)	Additional severe cases averted per 1000 additional doses (relative to routine age-based)	Clinical cases averted per 1000 doses (relative to no vaccination)	Severe cases averted per 1000 doses (relative to no vaccination)
Very low transmission: *Pf*PR_2–10_ = 1%						
Routine age-based	284 (195, 466)	9 (4, 16)	Ref	Ref	96 (66, 157)	3 (1, 6)
2y booster	296 (216, 476)	9 (4, 16)	31 (− 44, 105)	1 (− 2, 3)	87 (63, 140)	3 (1, 5)
5y booster	358 (259, 561)	11 (5, 20)	198 (92, 345)	6 (2, 12)	107 (78, 168)	3 (1, 6)
10y booster	367 (274, 547)	12 (5, 21)	318 (215, 468)	9 (4, 20)	114 (85, 169)	4 (1, 6)
5y, 10y boosters	410 (310, 637)	13 (5, 23)	219 (166, 312)	8 (3, 13)	115 (87, 180)	4 (2, 7)
2y, 5y boosters	353 (274, 551)	11 (5, 19)	94 (46, 150)	3 (1, 6)	95 (74, 148)	3 (1, 5)
2y, 10y boosters	376 (279, 568)	12 (5, 20)	131 (77, 202)	4 (2, 7)	104 (77, 157)	3 (1, 6)
2y, 5y, 10y boosters	400 (304, 598)	12 (5, 22)	129 (86, 174)	4 (2, 7)	103 (78, 154)	3 (1, 6)
Catch-up 6m-2y	310 (228, 516)	10 (4, 18)	131 (25, 254)	3 (− 1, 7)	97 (71, 161)	3 (1, 6)
Catch-up 6m-4y	356 (254, 569)	11 (4, 19)	163 (80, 313)	4 (1, 8)	105 (75, 168)	3 (1, 6)
Catch-up 6m-9y	426 (326, 647)	12 (5, 21)	171 (112, 237)	4 (2, 8)	112 (85, 170)	3 (1, 5)
Catch-up 6m-14y	493 (384, 748)	14 (6, 23)	166 (119, 237)	4 (2, 7)	118 (92, 179)	3 (1, 5)
Catch-up 5-9y	363 (268, 559)	11 (4, 19)	195 (95, 316)	4 (1, 10)	107 (79, 165)	3 (1, 6)
Catch-up 5-14y	430 (331, 667)	12 (5, 21)	185 (124, 280)	4 (2, 8)	114 (88, 177)	3 (1, 5)
Low transmission: *Pf*PR_2–10_ = 5%						
Routine age-based	733 (523, 1022)	15 (6, 22)	Ref	Ref	247 (177, 345)	5 (2, 8)
2y booster	762 (542, 1083)	15 (7, 23)	64 (− 20, 171)	1 (− 1, 5)	224 (159, 318)	5 (2, 7)
5y booster	906 (646, 1273)	18 (7, 27)	472 (325, 692)	8 (3, 14)	272 (194, 382)	5 (2, 8)
10y booster	928 (681, 1302)	18 (8, 27)	740 (558, 1080)	12 (6, 18)	287 (211, 403)	6 (2, 8)
5y, 10y boosters	1041 (756, 1452)	20 (9, 32)	545 (409, 733)	10 (5, 16)	293 (213, 409)	6 (3, 9)
2y, 5y boosters	890 (643, 1264)	18 (8, 26)	225 (160, 317)	4 (2, 7)	240 (173, 341)	5 (2, 7)
2y, 10y boosters	936 (673, 1285)	18 (8, 27)	329 (226, 427)	5 (2, 9)	258 (185, 355)	5 (2, 8)
2y, 5y, 10y boosters	1002 (731, 1406)	20 (8, 30)	303 (231, 424)	5 (2, 9)	258 (189, 362)	5 (2, 8)
Catch-up 6m-2y	803 (578, 1130)	16 (7, 24)	302 (155, 497)	4 (0, 10)	250 (180, 352)	5 (2, 8)
Catch-up 6m-4y	864 (623, 1234)	16 (7, 25)	330 (197, 494)	4 (2, 8)	255 (184, 365)	5 (2, 7)
Catch-up 6m-9y	1008 (739, 1427)	19 (8, 28)	344 (248, 459)	4 (2, 7)	264 (193, 374)	5 (2, 7)
Catch-up 6m-14y	1141 (843, 1587)	19 (8, 29)	336 (254, 458)	4 (2, 6)	272 (201, 379)	5 (2, 7)
Catch-up 5-9y	873 (643, 1249)	17 (7, 25)	373 (240, 518)	4 (1, 8)	258 (190, 368)	5 (2, 7)
Catch-up 5-14y	1007 (745, 1436)	18 (8, 26)	361 (251, 482)	4 (2, 6)	268 (198, 381)	5 (2, 7)
Moderate transmission: *Pf*PR_2–10_ = 25%						
Routine age-based	1697 (1261, 2305)	25 (14, 38)	Ref	Ref	573 (426, 779)	8 (5, 13)
2y booster	1743 (1302, 2404)	26 (14, 38)	146 (57, 264)	1 (− 2, 6)	512 (382, 707)	8 (4, 11)
5y booster	2043 (1530, 2761)	27 (16, 40)	968 (666, 1296)	5 (2, 11)	613 (458, 830)	8 (5, 12)
10y booster	2015 (1492, 2677)	25 (15, 39)	1216 (843, 1602)	3 (− 3, 8)	625 (462, 829)	8 (5, 12)
5y, 10y boosters	2334 (1713, 3100)	28 (16, 42)	1085 (778, 1443)	5 (2, 8)	660 (484, 874)	8 (5, 12)
2y, 5y boosters	2069 (1515, 2804)	28 (16, 41)	482 (321, 688)	4 (1, 6)	559 (410, 759)	7 (4, 11)
2y, 10y boosters	2066 (1554, 2785)	28 (15, 40)	595 (428, 806)	3 (1, 5)	572 (429, 772)	8 (4, 11)
2y, 5y, 10y boosters	2271 (1655, 3087)	29 (17, 43)	642 (451, 862)	4 (2, 6)	587 (428, 799)	7 (4, 11)
Catch-up 6m-2y	1799 (1340, 2487)	26 (15, 39)	460 (274, 812)	5 (− 2, 13)	562 (418, 776)	8 (5, 12)
Catch-up 6m-4y	1883 (1404, 2562)	27 (15, 40)	481 (305, 732)	4 (0, 9)	556 (415, 756)	8 (5, 12)
Catch-up 6m-9y	2056 (1540, 2819)	27 (15, 41)	431 (308, 654)	3 (0, 5)	540 (405, 740)	7 (4, 11)
Catch-up 6m-14y	2179 (1595, 2979)	27 (15, 41)	387 (273, 586)	2 (0, 4)	521 (381, 711)	6 (4, 10)
Catch-up 5-9y	1840 (1371, 2535)	25 (15, 39)	383 (244, 675)	1 (− 2, 5)	544 (405, 748)	7 (4, 11)
Catch-up 5-14y	2000 (1465, 2699)	25 (15, 39)	349 (224, 514)	1 (− 1, 3)	532 (390, 717)	7 (4, 10)
Moderately high transmission: *Pf*PR_2–10_ = 45%						
Routine age-based	1934 (1393, 2605)	29 (15, 46)	Ref	Ref	654 (470, 878)	10 (5, 15)
2y booster	1993 (1479, 2729)	29 (14, 46)	162 (65, 301)	1 (− 3, 5)	586 (434, 801)	9 (4, 14)
5y booster	2260 (1639, 3087)	28 (16, 45)	835 (605, 1274)	1 (− 7, 6)	678 (493, 927)	9 (5, 13)
10y booster	2153 (1560, 2995)	28 (14, 44)	800 (482, 1361)	− 4 (− 11, 3)	667 (483, 930)	9 (4, 14)
5y, 10y boosters	2498 (1803, 3447)	29 (16, 45)	935 (655, 1424)	1 (− 3, 4)	706 (509, 973)	8 (4, 13)
2y, 5y boosters	2304 (1674, 3127)	30 (17, 48)	480 (342, 697)	2 (0, 5)	623 (452, 843)	8 (4, 13)
2y, 10y boosters	2282 (1649, 3098)	29 (16, 47)	506 (350, 731)	2 (− 2, 4)	631 (456, 854)	8 (4, 13)
2y, 5y, 10y boosters	2505 (1792, 3420)	30 (16, 49)	613 (429, 862)	2 (0, 4)	647 (463, 884)	8 (4, 13)
Catch-up 6m-2y	2015 (1471, 2734)	30 (15, 47)	358 (113, 570)	5 (− 3, 13)	631 (461, 853)	9 (5, 15)
Catch-up 6m-4y	2074 (1500, 2869)	30 (15, 46)	311 (169, 537)	3 (− 2, 9)	614 (443, 850)	9 (5, 14)
Catch-up 6m-9y	2150 (1530, 2994)	29 (16, 47)	239 (135, 436)	1 (− 2, 4)	565 (401, 786)	8 (4, 12)
Catch-up 6m-14y	2186 (1601, 3066)	29 (15, 46)	214 (116, 353)	1 (− 1, 2)	521 (383, 731)	7 (4, 11)
Catch-up 5-9y	2024 (1481, 2758)	28 (15, 45)	206 (35, 363)	− 1 (− 6, 4)	597 (438, 815)	8 (4, 13)
Catch-up 5-14y	2067 (1500, 2811)	28 (14, 45)	153 (41, 264)	0 (− 3, 2)	550 (398, 745)	7 (4, 12)
High transmission: *Pf*PR_2–10_ = 65%						
Routine age-based	1773 (1285, 2649)	28 (9, 61)	Ref	Ref	599 (434, 896)	9 (3, 21)
2y booster	1833 (1294, 2692)	27 (10, 62)	131 (− 3, 303)	0 (− 3, 4)	538 (379, 791)	8 (3, 18)
5y booster	1952 (1433, 2882)	27 (9, 62)	518 (304, 853)	− 1 (− 6, 3)	586 (431, 864)	8 (3, 19)
10y booster	1834 (1332, 2726)	26 (8, 60)	286 (57, 625)	− 6 (− 14, 1)	569 (414, 844)	8 (2, 19)
5y, 10y boosters	2063 (1534, 3081)	26 (9, 62)	543 (319, 865)	− 2 (− 5, 2)	582 (433, 867)	7 (3, 18)
2y, 5y boosters	2022 (1462, 3000)	28 (10, 63)	343 (218, 582)	1 (− 2, 3)	547 (395, 811)	8 (3, 17)
2y, 10y boosters	1937 (1423, 2892)	27 (9, 62)	273 (174, 520)	0 (− 3, 2)	536 (394, 799)	8 (3, 17)
2y, 5y, 10y boosters	2089 (1559, 3130)	28 (9, 62)	394 (244, 640)	0 (− 2, 2)	540 (403, 809)	7 (2, 16)
Catch-up 6m-2y	1802 (1326, 2704)	29 (10, 64)	219 (2, 518)	4 (− 2, 15)	563 (414, 845)	9 (3, 20)
Catch-up 6m-4y	1844 (1282, 2760)	28 (10, 64)	172 (− 16, 369)	2 (− 3, 8)	545 (379, 816)	8 (3, 19)
Catch-up 6m-9y	1823 (1301, 2806)	28 (9, 63)	88 (10, 232)	0 (− 2, 3)	479 (341, 739)	7 (2, 17)
Catch-up 6m-14y	1830 (1306, 2773)	27 (8, 64)	56 (− 14, 164)	− 1 (− 3, 2)	438 (312, 662)	6 (2, 15)
Catch-up 5-9y	1781 (1302, 2679)	27 (9, 61)	27 (− 68, 181)	− 2 (− 6, 3)	527 (385, 791)	8 (3, 18)
Catch-up 5-14y	1779 (1291, 2686)	27 (8, 61)	22 (− 65, 109)	− 1 (− 4, 1)	473 (342, 714)	7 (2, 16)

Comparing across transmission settings, the median value of catch-up campaigns per additional dose is greatest when targeting children under 2 years in 25–45% *Pf*PR_2–10_ settings when considering severe disease and when targeting children under 4 years in 25% *Pf*PR_2–10_ settings when considering clinical disease (Fig. 1). The per additional dose impact on severe cases is highest when targeting children aged 6 months–2 years in the 25–45% *Pf*PR_2–10_ settings but is higher in the lower transmission settings when targeting older children. The per additional dose impact on uncomplicated malaria is estimated to increase with transmission intensity up to 25% *Pf*PR_2–10_ and then decline again in the highest transmission settings. For example, over the 30-year simulation, a supplementary catch-up campaign targeting children aged 6 months to 4 years is projected to avert 311 (95% CrI 169–537) additional clinical and 3 (95% CrI − 2–9) additional severe cases per 1000 additional doses in a perennial setting with 45% *Pf*PR_2–10_ versus 330 (95% CrI 197–494) additional clinical and 4 (95% CrI 2–8) additional severe cases per additional 1000 doses in a perennial setting with 5% *Pf*PR_2–10_ (Table 2).

When deciding where to allocate extra vaccine doses, supplemental catch-up campaigns in moderate to high transmission areas often result in higher incremental efficiency than adding routine age-based vaccination in lower transmission areas. For example, implementing a catch-up campaign to children under 4 in a 45% *Pf*PR_2–10_ setting or to any age group at 5% or 25% *Pf*PR_2–10_ would have higher per additional dose incremental efficiency in terms of additional clinical cases averted per 1000 additional doses compared to the per-dose impact of routine age-based vaccination in 1–5% *Pf*PR_2–10_ (Fig. 1, Table 2). Similarly, for severe cases, catch-up vaccination of young children up to age 4 at 25% *Pf*PR_2–10_ and up to age 2 at 45% *Pf*PR_2–10_ averts more severe cases per additional dose than routine vaccination in a 1% *Pf*PR_2–10_ setting (Table 2). These trends are similar in seasonal settings; however, catch-up campaigns to young children at higher transmission are marginally more efficient per additional dose in seasonal versus perennial settings because the catch-up campaigns were timed in our model to be before the peak in transmission and hence provided the highest level of protection during the period of highest risk (Additional file 1: Fig. S4).

**Fig. 2:**
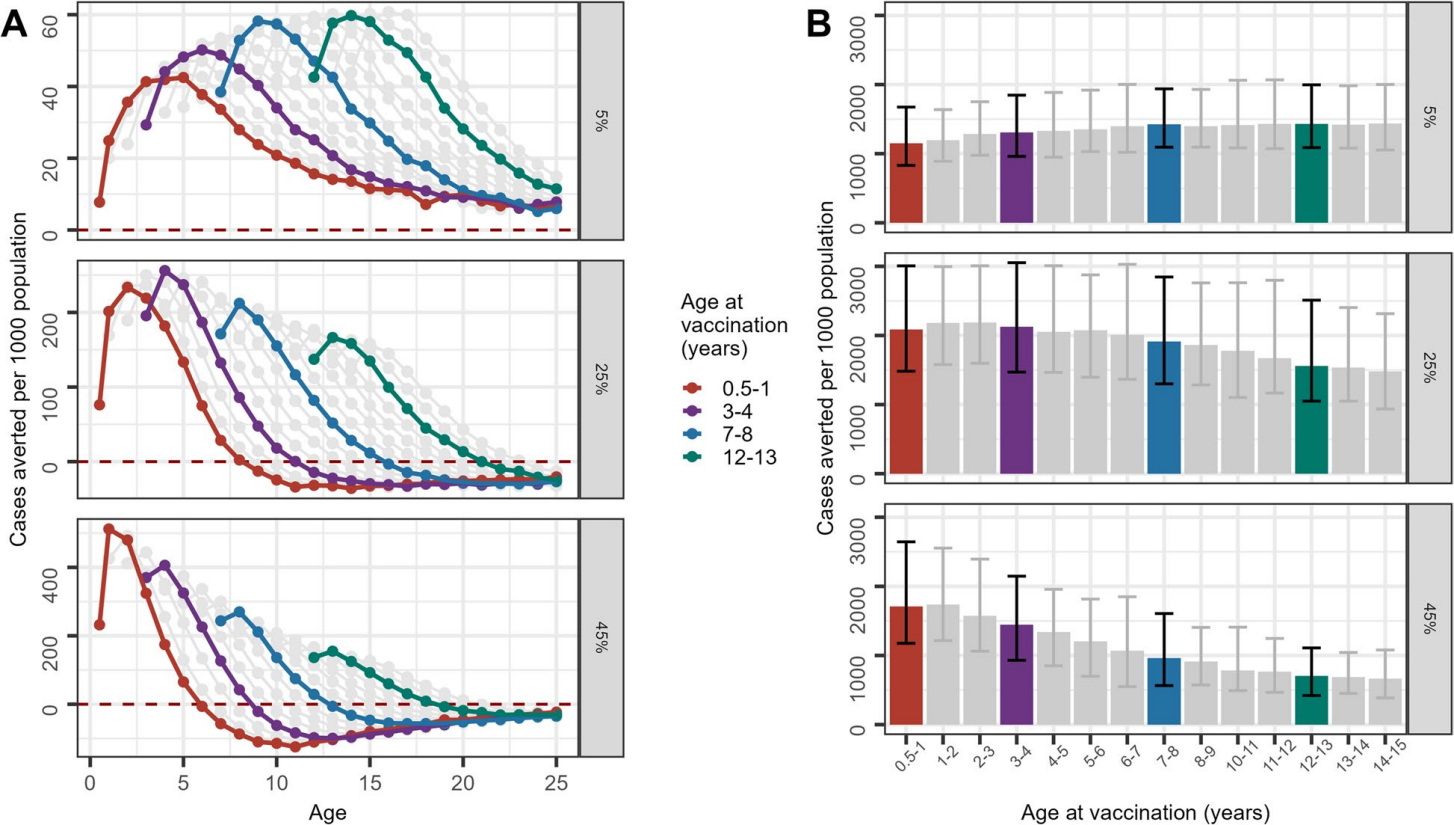
Clinical cases averted per 1000 population. Cases averted per 1000 people are summarised over 30 years in a cohort of children aged 6 months to 14 years old at vaccination in perennial settings. **A** shows the cases averted per 1000 people by age. Each point on **A** refers to the number of cases averted for each age group in a cohort vaccinated at specified ages. **B** shows the sum of cases averted per 1000 people in a cohort of people vaccinated at specified ages (x-axis). All ages at vaccination (age groups from 6 months to 14 years) are plotted; a few ages at vaccination have been highlighted in colour. The age range shown in **A** is up to 25 years; totals plotted in **B** are the sums of cases averted in cohorts over the 30-year simulation (e.g. the cohort vaccinated at age 14 would be followed until they are 44 years old at the end of the simulation)

Figure 2 shows how the projected number of clinical cases averted in a cohort of children vaccinated within different age groups changes as the cohort ages, depending on the age at first vaccination. In a low transmission setting (5% *Pf*PR_2–10_), the greatest number of cases averted immediately after vaccination increases with age at vaccination (for example, the group illustrated in green vaccinated at 12–13 years of age) (Fig. 2A). Although vaccination of older children is more efficient over the whole life course, a similar number of cases are averted across all ages at vaccination (Fig. 2B). In contrast, at high transmission (45% *Pf*PR_2–10_), vaccination of older children results in fewer total cases averted per person over the life course compared to vaccination of younger children; for example, vaccination to children aged 0.5–1 year averted 1710 (95% CrI 1176–2644) cases per 1000 population, while vaccination to children aged 12–13 years averted 706 (95% CrI 421–1110) cases per 1000 population over the life course.

Both routine age-based vaccination and routine plus catch-up campaigns are projected to reduce the overall burden of malaria and shift the age distribution of clinical cases (Additional file 1: Table S4). At low perennial transmission (5% *Pf*PR_2–10_), 10% (95% CrI 6–17) of cases are estimated to be among children under 5 years before vaccination, reducing to 6% (95% CrI 4–10) with routine age-based vaccination and to similar values with supplemental catch-up campaigns. Before vaccination in a high perennial transmission setting (45% *Pf*PR_2–10_), 41% (95% CrI 27–66) of cases are estimated to be in children under 5 years, in line with other modelling studies [15]. The percentage of cases in children under 5 years fell to 29% (95% CrI 19–46) with routine vaccination and to 28% (95% CrI 18–44) with additional catch-up campaigns (Additional file 1: Table S4). This change in age distribution follows shifting age burdens observed after effective control interventions such as indoor residual spraying and the distribution of long-lasting insecticide-treated nets among children [46] or prophylaxis given to infants [47] seen in field settings. The shifted age distribution also aligns with other modelling studies showing infection-blocking interventions such as pre-erythrocytic vaccination or seasonal malaria chemoprevention shifting the age distribution to older children [13, 48, 49].

### Extra booster doses

**Fig. 3:**
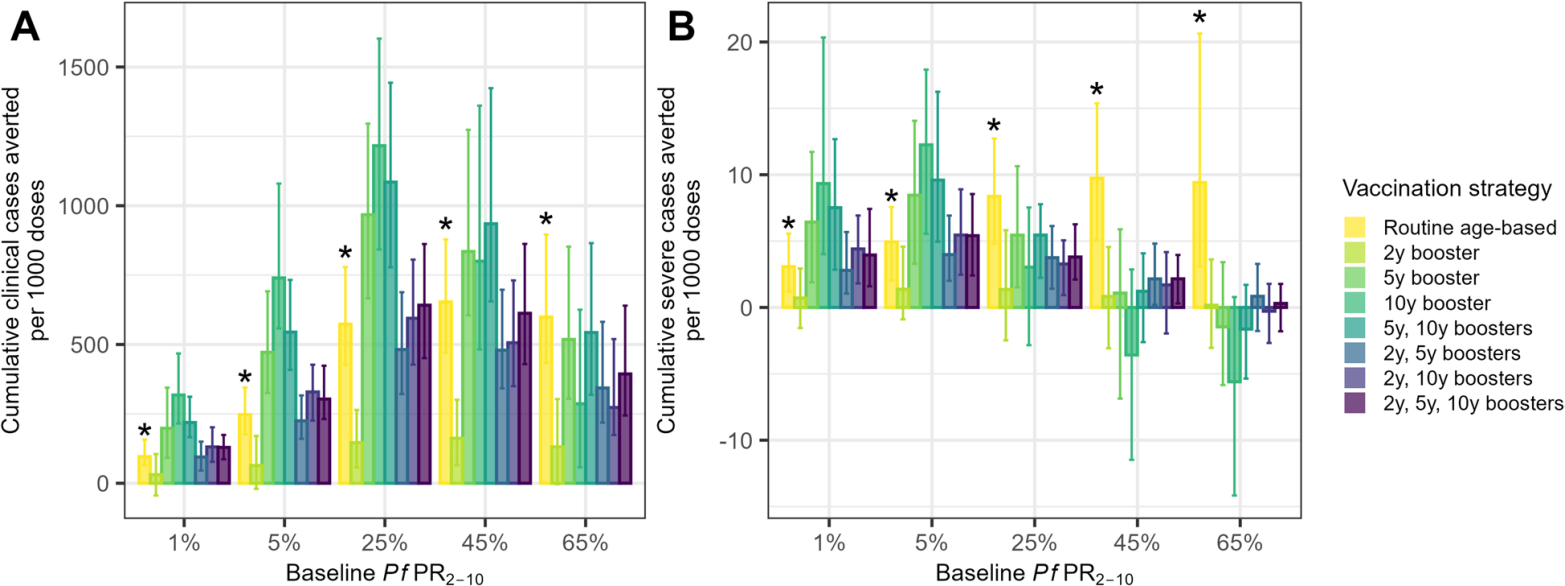
Extra booster impact per 1000 additional doses, perennial setting. Cumulative additional clinical cases (**A**) and severe cases (**B**) averted per 1000 additional doses in a perennial setting over the 30-year simulation. Values for routine age-based vaccination (bars with * above) show the outcome per 1000 doses delivered relative to no vaccination, while all other plotted strategies show additional outcomes averted per additional doses delivered relative to routine age-based vaccination. Age-based routine vaccination in 6-month-olds with supplementary catch-up vaccination in older children is assumed in all catch-up scenarios. The number of clinical or severe cases is compared to the routine age-based scenario baseline. The number of doses is calculated as the total number of doses under the specified vaccination strategy compared to a baseline scenario of routine age-based vaccination. The bars show median values, and the error bars show 95% credible intervals from 50 stochastic model runs. Negative values indicate more clinical or severe cases relative to the baseline of no vaccination. Note that the y-axes in plots **A** and **B** are different

Figure 3 shows the impact of supplemental booster doses at 2, 5, and/or 10 years after routine age-based vaccination in perennial settings. A single extra booster dose 2 years after the third dose provides minimal benefit in terms of uncomplicated clinical cases averted per additional dose compared to routine age-based vaccination and is less efficient compared to any other extra booster dose strategy, likely because of re-vaccination before vaccine-induced protection wanes (Fig. 3). Likewise, an extra booster at 2 years combined with other boosters at 5 and/or 10 years tends to be less efficient per additional dose compared to booster doses delivered only at 5 and/or 10 years (Fig. 3A). However, considering severe cases, boosters at 2 and 5 years is the strategy with the most severe cases averted per additional dose at 65% *Pf*PR_2–10_, reflecting the large burden among very young children at high transmission (Fig. 3B).

The scenarios with boosters at 5, 10, and 5 + 10 years consistently avert the most uncomplicated clinical cases per additional dose, though the single 10-year booster performs the best in settings 25% *Pf*PR_2–10_ and lower, while the 5 + 10-year booster strategy performs the best in higher transmission settings. Considering severe cases, the 10-year booster averts the most cases per additional dose in low transmission settings (e.g. 12 (95% CrI 6–18) in a 5% *Pf*PR_2–10_ perennial setting), but the least in high transmission settings (e.g. − 6 (95% CrI − 14–1) in a 65% *Pf*PR_2–10_ perennial setting) (Table 2), reflecting the low proportion of cases among older children at high transmission.

Adding one to three extra booster doses at 2, 5, and/or 10 years, except for a single extra booster at 2 years, in settings with *Pf*PR_2–10_ 5% or above, has higher predicted incremental efficiency in terms of additional cases averted per additional dose (e.g. medians of 800–1216 clinical cases per 1000 additional doses at 5- or 10-year boosters at 25–45% *Pf*PR_2–10_) compared to routine age-based vaccination in settings with 1–5% *Pf*PR_2–10_ (medians of 96–247 clinical cases per 1000 doses) (Fig. 3, Table 2). However, for severe cases, scenarios with booster doses at 5 or 10 years at 25% *Pf*PR_2–10_ are roughly equivalent to routine vaccination at 1–5% *Pf*PR_2–10_, but introducing boosters in higher transmission settings is less efficient than introducing routine age-based vaccination at low transmission.

The patterns of projected impacts of the additional booster strategies are relatively similar for both clinical cases and severe cases averted per population at low transmission, where the age pattern of malaria burden is evenly distributed across age groups and where delayed malaria due to waning vaccine-induced immunity is less evident (Additional file 1: Fig. S5A). However, at higher transmission, the relative impact of the additional booster doses on clinical cases versus severe cases diverges. At the highest levels of transmission (45% and 65% *Pf*PR_2–10_), there is a significant benefit in averting clinical cases but a less clear benefit in averting severe disease with extra booster doses in terms of absolute cases averted (Additional file 1: Fig. S5) and cases averted per additional dose relative to routine age-based vaccination (Fig. 3), due to the concentration of severe disease in younger age groups. In contrast, in lower transmission settings, extra boosters provide added protection against severe disease in the older population (Additional file 1: Fig. S5B). Seasonal settings show similar patterns (Additional file 1: Fig. S6).

**Fig. 4:**
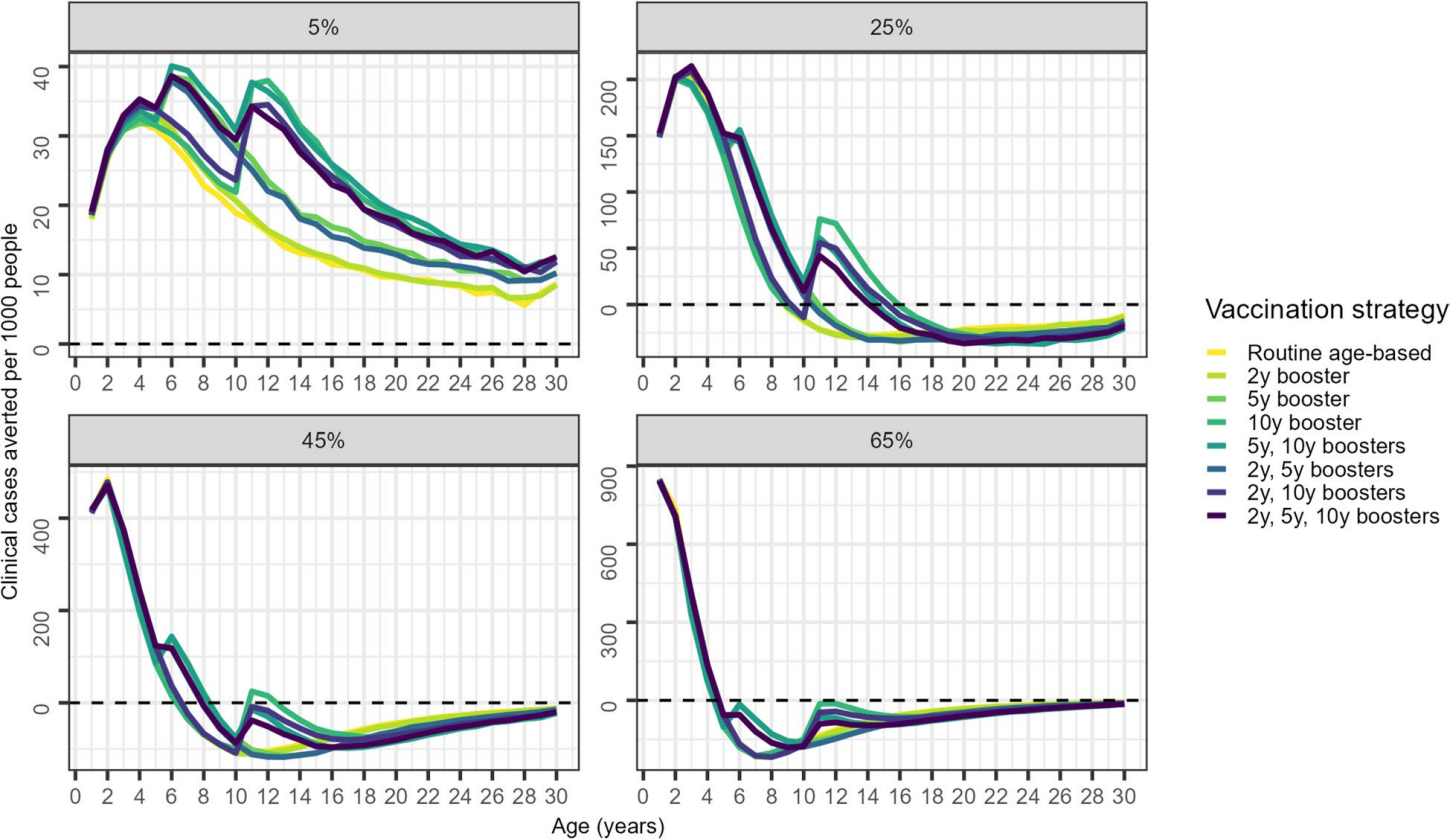
Additional booster impact: clinical cases averted per 1000 people by age. Cohort view of clinical cases averted per 1000 people in each 1-year age group compared to a no vaccination baseline scenario in a perennial setting. For example, for age 10, this is a snapshot summary of the state of all children aged 10 at any point during the simulation across all vaccinated cohorts. The coloured lines are the median values, and the shaded regions are the 95% credible intervals over the 60 cohorts and 50 stochastic parameter draws. The no vaccination baseline is indicated by the dashed lines

In cohorts vaccinated with a routine age-based strategy (Fig. 4), extra boosters recover the vaccine-induced protection that otherwise wanes over time, though the extent to which protection is recovered varies by transmission intensity. At 25% *Pf*PR_2–10_ and above, the number of cases averted as the cohort ages is projected to fall over the first few years after vaccination; extra booster doses are projected to both delay and reduce the subsequent rebound in clinical cases. However, at 65% *Pf*PR_2–10_, two to three additional boosters delivered after protection from the primary series wanes are not sufficient in themselves to entirely offset the rebound in incidence. Despite the rebound, the large number of cases averted in young children means that the total number of clinical and severe cases averted per person remains positive (Additional file 1: Fig. S6).

In contrast to their impact on clinical cases, extra boosters provide limited additional benefit in averting severe cases compared to the routine age-based primary series, especially at moderate to high transmission (Additional file 1: Fig. S7). At 25%, 45%, and 65% *Pf*PR_2–10_, a cohort of children vaccinated under the routine age-based strategy is predicted to have a higher incidence of clinical and severe malaria than the unvaccinated cohort (shown by the horizontal dotted line) for multiple years from the time when vaccine protection wanes (Fig. 4, Additional file 1: Fig. S7). Waning of vaccine protection is slower for both clinical and severe cases at lower transmission (e.g. the cohort who received routine age-based vaccination had zero vaccine efficacy against severe malaria in the model around 8 years post-vaccination at 5% *Pf*PR_2–10_ and around 2.5 years post-vaccination at 65% *Pf*PR_2–10_). However, the length of the rebound of malaria is not influenced by extra booster doses and decreases as transmission intensity increases (e.g. returns to baseline at around age 26 years at 25% *Pf*PR_2–10_ and around age 10 years at 65% *Pf*PR_2–10_). The length of time for severe incidence to return to baseline is shorter for severe malaria compared to clinical malaria, possibly due to our model assumption that susceptibility to severe disease being partially physiological, with the peak risk at younger ages. This result is consistent with RTS,S/AS01 trial data from 7 years of follow-up [11].

### Combination of catch-up vaccination and extra booster doses

At each perennial transmission intensity and seasonality, the greatest number of clinical cases averted per 1000 people is projected to occur with catch-up vaccination to children aged 6 months to 14 years combined with two extra booster doses at 5 and 10 years after the primary series except for 65% *Pf*PR_2–10_, where catch-up to children aged 6 months to 9 years plus three extra booster doses at 2, 5, and 10 years averts slightly more cases. Within a transmission setting, estimates of the number of clinical cases averted are very similar in many of the combination strategies. In perennial settings, the number of clinical cases averted per 1000 people for this strategy ranges from a median of 611 (95% CrI 480–897) at 1% *Pf*PR_2–10_ to 2820 (95% CrI 2083–3803) at 25% *Pf*PR_2–10_ (Additional file 1: Table S5). The most severe cases averted per person is with catch-up campaigns to children up to 14 years plus two extra boosters at 5 and 10 years in low transmission settings (1% *Pf*PR_2–10_: 18 (95% CrI 7–30), 5% *Pf*PR_2–10_: 25 (95% CrI 11–38)), catch-up to children up to 14 years plus three extra boosters at 25% *Pf*PR_2–10_ (31 (95% CrI 18–47)), catch-up to children up to 4 years plus two extra boosters at 2 and 5 years at 4% *Pf*PR_2–10_ (32 (95% CrI 17–49)), and catch-up to children up to 9 years plus three extra boosters at 65% *Pf*PR_2–10_ (29 (95% CrI 8–65)).

Over the 30-year simulation, the greatest number of cases averted per 1000 additional doses compared to a baseline of routine-age-based vaccination is projected to occur with catch-up vaccination to children 6 months to 2 years old at all transmission levels, combined with an extra booster at 10 years in low transmission settings and two extra boosters at 5 and 10 years in moderate to high transmission settings (25–45%) (Additional file 1: Table S6). When considering severe cases, the strategies that avert the most cases for each additional dose have more similar impact per 1000 doses, when considering multiple combinations of catch-up campaigns to children up to 4 years old with one or two extra booster doses.

### Efficiency

**Fig. 5:**
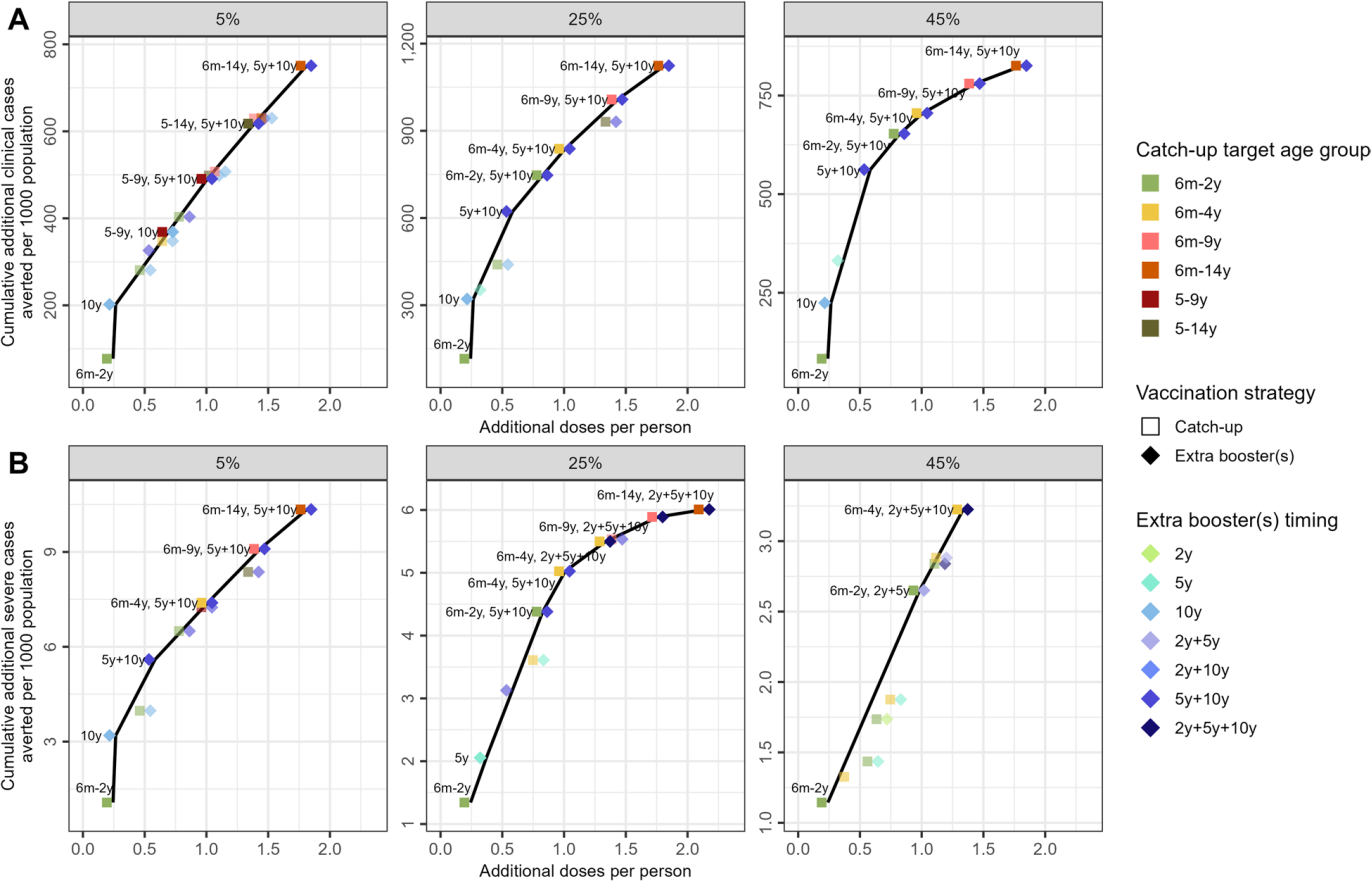
Efficiency frontier: additional cases averted compared to additional doses delivered. Perennial settings are plotted. Square points show vaccination strategies with a catch-up campaign, with colours indicating the age group, and diamond-shaped points show extra booster strategies, with colours indicating the booster timing. Both catch-up campaigns and extra boosters are supplementary to routine age-based vaccination. Labels indicate the number of booster doses (up to three additional boosters at 2, 5, and/or 10 years after the third dose in the routine age-based primary series) followed by the age group vaccinated in a catch-up campaign. The black line connects the points on the efficiency frontier; all strongly dominated strategies (higher cost, lower benefit) were removed. Extended dominated strategies are semi-transparent. **A** shows efficiency frontiers for clinical cases, and **B** shows efficiency frontiers for severe cases. Efficiency frontiers for seasonal settings are found in Additional file 1: Fig. S8

Figure 5 shows the efficiency frontier for a range of vaccination scenarios at different transmission levels for perennial settings (similar results for seasonal settings in Additional file 1: Fig. S8). The extra booster at 10 years is on the efficiency frontier in all settings for clinical cases, but only in the 5% *Pf*PR_2–10_ setting for severe cases. At 5% *Pf*PR_2–10_, catch-up scenarios targeting children over 5 years of age (not including children between 6 months and 5 years) are on the efficiency frontier only when considering clinical cases; for severe cases at the same transmission level, the most efficient strategies including catch-up campaigns always target children between age 6 months and 5 years, combined with extra booster doses. The value of catch-up vaccination in older age groups at moderate to high transmission is less clear than its value against clinical disease, as it depends on the extent to which severe disease is experienced at older ages. The strategies on the efficiency frontier for severe cases at high transmission are combinations of catch-up campaigns to children up to 4 years of age with extra booster doses. At all transmission levels, combinations of catch-up campaigns to target progressively wider age groups with two extra booster doses at 5 and 10 years are more efficient than including a third booster at 2 years post-primary series for clinical cases (Fig. 5A), but the three-booster combination strategies are more efficient than the two-dose strategies for severe cases.

### Sensitivity analysis of immunogenicity assumption

The main analysis assumes that the immunogenicity and efficacy of R21/Matrix-M is the same regardless of age. In Additional file 1, we present the sensitivity analysis results assuming that the antibody titres are 36% and 60% lower for children aged 5 years and older than those achieved by routine vaccination. This translates to a reduction of 5.7 percentage points in vaccine efficacy over 1 year for the immunogenicity scaler of 0.64 and 12.9 percentage points for the immunogenicity scaler of 0.4 (Additional file 1: Fig. S9), as described in the “Methods” section (Additional file 1: Figs. S9–S14, Table S7). Under the reduced immunogenicity assumptions, the scenarios where older children are vaccinated generally have lower impacts in terms of clinical and severe cases averted per person compared to scenarios with vaccination of younger children, but the difference is small for clinical and severe cases averted per person, with mean reductions across all vaccination strategies and transmission settings of 4.3% and 2.1% for clinical and severe cases, respectively, when assuming a scaler of 0.64, and of 8.6% and 4.0% for clinical and severe cases, respectively, when assuming a scaler of 0.4. There are slightly larger mean reductions for clinical and severe cases averted per additional dose of 16.1% and 15.0%, respectively, when assuming a scaler of 0.64, and 32.5% and 29.1%, respectively, when assuming a scaler of 0.4, relative to routine age-based vaccination. The scenarios on the efficiency frontier are broadly similar, except in low transmission settings, where the efficiency frontiers are modified due to most cases occurring in children over 5 years old, and strategies with a booster dose at 2 years post-primary series are more likely to appear when immunogenicity in older children is reduced. Despite the reduction in clinical and severe cases averted per 1000 additional doses with the assumption that immunogenicity in older children decreases, our conclusion that in many cases, expanding vaccination to older children through catch-up campaigns or extra booster doses in high transmission settings is often more efficient than introducing routine vaccination in low transmission settings.

## Discussion

Our results estimate that routine age-based vaccination, as currently recommended by WHO, is highly efficient, but that additional doses delivered through either expansion of targeted age groups through catch-up campaigns or via extra booster doses can provide additional benefits, though the value varies by transmission and seasonality setting. Prioritising extra booster doses to previously vaccinated children in areas of moderate to high transmission or targeting older children through catch-up vaccination in moderate transmission settings is predicted to often be more efficient per dose than expanding routine age-based vaccination to areas of low transmission when considering clinical cases. However, routine vaccination is almost always estimated to be the most efficient strategy in terms of reducing severe cases.

Catch-up vaccination aims to protect children who may have missed doses and/or are older than the recommended age group. It is commonly implemented for routine childhood vaccinations, including hepatitis B, and measles/mumps/rubella vaccines [50, 51]. We found that investment in supplementary catch-up vaccination campaigns with R21/Matrix-M to children under 4 years in 45% *Pf*PR_2–10_ and up to 14 years in 25% *Pf*PR_2–10_ transmission settings could avert more clinical and severe cases per dose compared to routine vaccination in 1–5% *Pf*PR_2–10_ settings. Catch-up vaccination to wider age groups also averts more cases per person, has relatively high marginal efficiency, especially when considering clinical cases in settings with low to moderate transmission, and may also improve equity in access to malaria control interventions. School-aged children have a high burden of uncomplicated malaria but are frequently unprotected due to lower ITN use and SMC ineligibility (typically limited to children up to 59 months of age), making them good targets for catch-up vaccination when the goal is to reduce uncomplicated cases [16–18, 18, 19].

A fourth dose of R21/Matrix-M 12 months post-primary series boosted efficacy and returned the anti-CSP antibody titres to levels close to the peak values after the primary series in previous studies [35]. Given that waning protection from pre-erythrocytic vaccines has been observed, an extra booster dose 2 years after the primary series is already recommended in settings with high malaria transmission risk and in seasonal settings [2]. However, we predict that a single extra booster dose could be more beneficial if delayed to 5 or 10 years after the primary series in many settings. We also show that two or three extra booster doses delivered 2, 5, and/or 10 years following dose 3 could avert more clinical cases per person and per additional dose than boosting solely at 2 years following dose 3 in most settings. Providing an extra dose at 2 years would boost protection before full vaccine efficacy wanes, limiting its additional benefit compared to delaying it to 5 years after dose 3. However, because the rebound of malaria occurs more rapidly in high transmission settings, providing an additional booster sooner than 5 years post-routine age-based vaccination may be warranted. Timing of additional boosters could therefore be tailored based on local evidence on the expected timing of rebound. Our results also suggest that extra boosters would avert additional severe cases per additional dose only in the low to moderate transmission settings where the period at risk extends to school-aged children and adults. Given the changing epidemiology of malaria resulting in a shift in both clinical and severe disease to older age groups as natural immunity declines due to vaccine-induced protection or decreasing transmission, empirical data are needed to support decisions on periods of highest risk.

If sufficient dose supply and funding are available, combining catch-up vaccination and extra booster doses is projected to avert more cases per person and per additional dose than either strategy alone, though the optimal target age group varies by transmission intensity. In most settings, the combination strategies were on the efficiency frontier and had the lowest expected loss. If extra vaccine doses are available in low transmission settings, many of the most incrementally efficient scenarios comprise catch-up campaigns to school-aged children with extra booster doses. In moderate to high transmission settings, strategies vaccinating progressively wider age groups but including children under 5 years old, in combination with 2 extra booster doses at 5 and 10 years after the primary series, are the most efficient per additional dose. However, considering severe cases at high transmission, our results suggest that targeting catch-up campaigns to children over 5 years is less impactful than adding extra booster doses to young children, as older children will already have acquired natural immunity.

Substantial funding cuts announced in early 2025 to government foreign aid budgets and global aid programmes, including the Global Fund and Gavi, critical sources of funding for malaria vaccines, have threatened the future of malaria control interventions, including vaccination [52–55]. In many malaria-endemic countries in SSA, health infrastructure weaknesses and limited health financing to cover delivery and logistics costs may further limit equitable access to malaria vaccines [56]. The choice of vaccination strategy may also depend on its acceptability to the target population. Although acceptance of the RTS,S/AS01 vaccine is high [57], some implementation strategies have been more successful than others. The Malaria Vaccine Implementation Programme (MVIP) for RTS,S/AS01 showed high community acceptance and demand for the vaccine, but they found that achieving high coverage of the fourth dose was challenging [3], which may limit the acceptability of some of the modelled scenarios with extra booster doses.

Our study has some limitations. In the absence of data across all age groups for R21/Matrix-M, we assumed in our main analysis that anti-CSP antibody response and subsequent vaccine efficacy were the same regardless of age. The phase III R21/Matrix-M trial data showed lower antibody titres in the 18–36-month group compared to the 5–17-month group, which was reflected in lower vaccine efficacy [7]. In the phase III RTS,S/AS01 trial, higher baseline anti-CSP antibodies, indicating more exposure to malaria, were associated with higher anti-CSP antibody titres post-vaccination [58, 59] while a pooled analysis of data from phase II RTS,S/AS01 studies showed vaccine efficacy to be highest in very young children, lower in children around 12 months of age, and then increasing in children up to 5 years old [60]. Factors like the maturity of the immune system, prior exposure to malaria, and the quality of immune response (antibody avidity) influence the immune response and vaccine efficacy; therefore, further trials, such as two ongoing trials of mass vaccination with R21/Matrix-M [38, 39], are needed to be able to capture potential variation in vaccine efficacy by age across different transmission settings. A sensitivity analysis in which the assumed immunogenicity in children over 5 years of the vaccine is 64% or 40% of that of the fitted immunogenicity parameters showed that, although the overall impact is expected to be lower, especially in scenarios where vaccination targets mostly older children, the scenarios on the efficiency frontier are largely the same. This result relies on the assumption that the relationship between antibody titres and efficacy is the same in older ages as in children aged 5–17 months.

We also made several simplifying assumptions. First, we assumed a population with no importation of new infections, which may have overestimated the benefits of vaccination in lower transmission settings where indirect protection will be more substantial in our model. Second, we assumed constant demography over the 30-year period to enable the effects of the vaccination schedules to be compared. However, given demographic shifts that are underway on the continent, the age distribution of the population is likely to change over time, leading to a relatively smaller proportion of the population being young children who could receive the recommended routine age-based vaccination.

Third, we assumed that the catch-up vaccination campaign started and finished on the same day as vaccine introduction, which may lead to overestimation of its impact, as in reality, catch-up campaigns may proceed over several months. Fourth, we assumed that the background interventions, which were not explicitly modelled, were held constant over the simulation period and had no synergistic effects with vaccination. Finally, we assumed that the per-unit costs of catch-up campaigns and booster dose strategies were the same, which could modify the efficiency frontiers if translated into cost. For example, catch-up campaigns may be more expensive because they require infrastructure outside of the Essential Programme on Immunization (EPI) [61], while routine age-based vaccination could be conducted at functioning EPI sites on established schedules. It is also possible that immunogenicity and efficacy of extra booster doses in older children could be affected by its co-administration with other vaccines in the EPI schedule, such as the human papillomavirus (HPV) vaccine in school-aged children. Additionally, the interventions with extra booster doses spread the costs of delivery and implementation over multiple years and would also require additional visits that could increase costs to the health system. Given this uncertainty, we chose not to conduct a full cost-effectiveness analysis at this stage.

In previous model predictions of the impact of R21/Matrix-M under routine age-based vaccination, the impact on clinical cases over a time horizon of 15 years increased with transmission intensity [24], while we found that after a peak at moderate transmission, the impact per additional dose with routine vaccination decreased with increasing transmission over a 30-year time horizon (Additional file 1: Fig. S2). Our model runs were twice as long, so summary calculations included a longer period of rebound malaria, which is more pronounced at high transmission. The impact on deaths in Schmit et al. plateaued at high transmission, which we also see in the impact on severe cases in our model runs (Additional file 1: Fig. S2).

Our model predictions showed a slightly higher benefit in seasonal compared to perennial settings for many of the catch-up strategies, an artefact of the optimised timing of the catch-up campaigns in seasonal settings to before the seasonal peak in transmission.

Routine vaccination with R21/Matrix-M in settings with endemic malaria delivered through an age-based or seasonal four-dose schedule was recommended by WHO in October 2023, with 25 countries implementing malaria vaccination as of March 2026, 23 of which are doing so via their EPI programmes under age-based strategies [8, 62, 63]. As the production of R21/Matrix-M increases to its predicted 200 million doses per year by 2026 [64], a sum that would cover the projected demand estimated by Gavi of 80–100 million annual doses by 2030 [65], malaria control programmes may wish to expand vaccination eligibility to older age groups or increase the number of booster doses administered if funding and delivery mechanisms remain viable.

## Conclusions

Our results demonstrate that expanding vaccination beyond the routine age-based programmes through catch-up campaigns, extra booster doses, or combination strategies, with target age groups and booster timing tailored to transmission intensity, could avert additional cases beyond routine administration, with per-additional-dose value approaching that of routine vaccination, and serve as valuable tools to further reduce malaria burden. Expanding eligible age groups through catch-up campaigns to children up to age 15 years is more efficient in averting clinical cases than routine vaccination in low transmission settings, but catch-up campaigns to children up to age 5 years in 5–25% *Pf*PR_2–10_ settings avert more clinical cases per dose than routine vaccination in 1–5% *Pf*PR_2–10_ settings. Implementing extra booster doses delayed until vaccine efficacy wanes 5 and/or 10 years after the primary series is often the most efficient strategy within and across transmission settings, except for at 65% *Pf*PR_2–10_, and requires relatively few extra doses to make a large impact on clinical cases. In most settings, routine age-based vaccination averts the most severe cases per dose delivered. As these results depend on the stated assumptions and modelling framework used in this study, empirical studies and further site-specific modelling to evaluate the impact of both strategies are warranted to inform future policy guidance regarding wider use of malaria vaccines.

## Supplementary Information

Additional file 1. Supplementary methods, Figures S1–S15, and Tables S1–S9. Fig. S1 Diagram of the human model of malaria transmission. Fig. S2 Cumulative clinical and severe cases averted per 1000 doses over the 30-year simulation with age-based, seasonal, and hybrid implementation. Fig. S3 Catch-up campaign impact per 1000 people, perennial settings. Fig. S4 Catch-up campaign impact per 1000 doses, seasonal setting. Fig. S5 Extra booster impact per 1000 people, perennial setting. Fig. S6 Extra booster impact per 1000 doses, seasonal setting. Fig. S7 Extra booster impact: severe cases averted per 1000 people. Fig. S8 Efficiency frontier in seasonal settings. Fig. S9 Comparison of scaled and non-scaled antibody titre and clinical efficacy. Fig. S10 Catch-up campaign impact per 1000 additional doses assuming age-scaled antibody titres. Fig. S11 Extra booster impact per 1000 additional doses, assuming age-scaled antibody titres. Fig. S12 Percent difference in cases averted per 1000 people between three different model assumptions. Fig. S13 Efficiency frontier for model runs with antibody dynamics in children 5 years of age and older, scaled by 0.64. Fig. S14 Efficiency frontier for model runs with antibody dynamics in children 5 years of age and older, scaled by 0.4. Fig. S15 Expected loss curves of all modelled scenarios for a range of perennial transmission intensities. Table S1 Transition rates between human infection states. Table S2 Default parameter values for the *malariasimulation* model. Table S3 Model assumptions and references. Table S4 Percent of clinical cases by age group and vaccination strategy. Table S5 Outcomes averted by combination vaccination strategies. Table S6 Percent of clinical and severe cases averted by age group and vaccination strategy. Table S7 Comparison of strategies on the efficiency frontier under different immunogenicity assumptions. Table S8 Outcomes averted per 1000 people, per 1000 additional doses, and per 1000 total doses in a perennial setting, assuming antibody titres in children 5 years and over are scaled to 0.64. Table S9 Outcomes averted per 1000 people, per 1000 additional doses, and per 1000 total doses in a perennial setting, assuming antibody titres in children 5 years and over are scaled to 0.4.

## Data Availability

The code required to rerun the analysis in this study is available at (https://github.com/kellymccain28/catchup/_extraboosters). The transmission model used in the study is freely available at (https://github.com/mrc-ide/malariasimulation) (v 2.0.1), and a modified version of this model to incorporate immunogenicity scaling by age is available at (https://github.com/kellymccain28/malariasimulation/_agescaling).

## Abbreviations

CrI: Credible interval
CSP: Circumsporozoite protein
EPI: Essential Programme on Immunization
GMR: Geometric mean ratio
HPV: Human papillomavirus
ICER: Incremental cost-effectiveness ratio
*Pf*PR_2-10_: *P. falciparum* Parasite prevalence by microscopy in children aged 2–10 years
SMC: Seasonal malaria chemoprevention
SSA: Sub-Saharan Africa
WHO: World Health Organization
