# Supplementary material for: Public health impact of catch-up vaccination or additional booster doses with pre-erythrocytic malaria vaccine R21/Matrix-M: a modelling study
Source: BMC Med. 2026 Mar 25;24:286. doi: 10.1186/s12916-026-04822-y (PMC13137673; doi:10.1186/s12916-026-04822-y)
Supplement: Supplementary file 1 — Additional file 1. Supplementary methods, Figures S1–S15, and Tables S1–S9. Fig. S1 Diagram of the human model of malaria transmission. Fig. S2 Cumulative clinical and severe cases averted per 1000 doses over the 30-year simulation with age-based, seasonal, and hybrid implementation. Fig. S3 Catch-up campaign impact per 1000 people, perennial settings. Fig. S4 Catch-up campaign impact per 1000 doses, seasonal setting. Fig. S5 Extra booster impact per 1000 people, perennial setting. Fig. S6 Extra booster impact per 1000 doses, seasonal setting. Fig. S7 Extra booster impact: severe cases averted per 1000 people. Fig. S8 Efficiency frontier in seasonal settings. Fig. S9 Comparison of scaled and non-scaled antibody titre and clinical efficacy. Fig. S10 Catch-up campaign impact per 1000 additional doses assuming age-scaled antibody titres. Fig. S11 Extra booster impact per 1000 additional doses, assuming age-scaled antibody titres. Fig. S12 Percent difference in cases averted per 1000 people between three different model assumptions. Fig. S13 Efficiency frontier for model runs with antibody dynamics in children 5 years of age and older, scaled by 0.64. Fig. S14 Efficiency frontier for model runs with antibody dynamics in children 5 years of age and older, scaled by 0.4. Fig. S15 Expected loss curves of all modelled scenarios for a range of perennial transmission intensities. Table S1 Transition rates between human infection states. Table S2 Default parameter values for the malariasimulation model. Table S3 Model assumptions and references. Table S4 Percent of clinical cases by age group and vaccination strategy. Table S5 Outcomes averted by combination vaccination strategies. Table S6 Percent of clinical and severe cases averted by age group and vaccination strategy. Table S7 Comparison of strategies on the efficiency frontier under different immunogenicity assumptions. Table S8 Outcomes averted per 1000 people, per 1000 additional doses, and per 1000 total doses in a pe [file 12916_2026_4822_MOESM1_ESM.docx]

**Supplementary information**

Public health impact of catch-up vaccination or additional booster doses with pre-erythrocytic malaria vaccine R21/Matrix-M: a modelling study

Kelly McCain*^a^; Hillary M Topazian^a^; Joseph D. Challenger^a^; Lucy Okell^a^; Peter Winskill^a^; Azra C. Ghani^a,b,c^

^a^MRC Centre for Global Infectious Disease Analysis, School of Public Health, Imperial College London, London, United Kingdom

^b^Saw Swee Hock School of Public Health, National University of Singapore and National University Health System, Singapore

^c^Lee Kong Chian School of Medicine, Nanyang Technological University, Singapore

*Corresponding author

E-mail: [k.mccain22@imperial.ac.uk](mailto:k.mccain22@imperial.ac.uk) (KM)

Contents

[Supplementary Methods 3](#_Toc208951221)

[Malaria transmission model overview 3](#_Toc208951222)

[Immunity 5](#_Toc208951223)

[Vector model 6](#_Toc208951224)

[Seasonality 6](#_Toc208951225)

[Interventions 7](#_Toc208951226)

[Treatment 7](#_Toc208951227)

[Vaccination 7](#_Toc208951228)

[Model parameter values 8](#_Toc208951229)

[Supplementary Results 12](#_Toc208951230)

[Sensitivity analysis of immunogenicity assumption 39](#_Toc208951231)

[Incremental Efficiency analysis 47](#_Toc208951232)

[Extended methods 47](#_Toc208951233)

[Expected loss 47](#_Toc208951234)

[References 50](#_Toc208951235)

# Supplementary Methods

## Malaria transmission model overview

In the human component of the model, individuals are born with a certain level of maternally acquired immunity that wanes exponentially over six months and after which they are susceptible (state
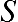
) to *P. falciparum* infection through infectious mosquito bites. The force of infection acting on individual *i* ($\Lambda_{i}$) depends on the entomological inoculation rate (EIR) and the age-based probability of infection which is modulated by pre-erythrocytic immunity. After a latent period ($d_{E}$), infected individuals may develop clinical disease or asymptomatic infection (state $A$), depending on their level of acquired immunity from cumulative exposure to malaria. A proportion of those with clinical disease are successfully treated at probability $f_{T}$, moving to the treated state $T$, and others are either not treated or fail treatment and develop disease at probability $1-f_{T}$. Treated individuals recover at rate $r_{T}$ before returning to the susceptible state but retain partial protection from reinfection dependent on the treatment drug that wanes over time according to a Weibull survivorship curve ^29^. Those with unsuccessful treatment move first to the asymptomatic state at rate $r_{D}$, then to a subpatent infection state once the parasite load falls below the detectable limit at rate $r_{A}$ before returning to the susceptible state at rate $r_{U}$. Superinfection from diseased ($D$), asymptomatic ($A$), and subpatent ($U$) states is possible. The progression of an infected human through the model states is shown in Figure S1 and the associated rates are summarised in Table S1.

Throughout the simulation, the population size remains constant. When individuals in the model die at daily age-specific death rates derived from the UNWPP Africa region demography and African neonatal mortality from UNICEF ^30^, they are replaced by a birth of an individual exposed to the same level of malaria exposure, defined by the biting rate. Deaths within the model are assumed to be from natural causes; we do not explicitly model deaths from malaria.

Biting rates are dependent on a relative biting rate assigned at birth, which changes over the lifespan of the individual as a function of body size which modulates the risk of malaria exposure.


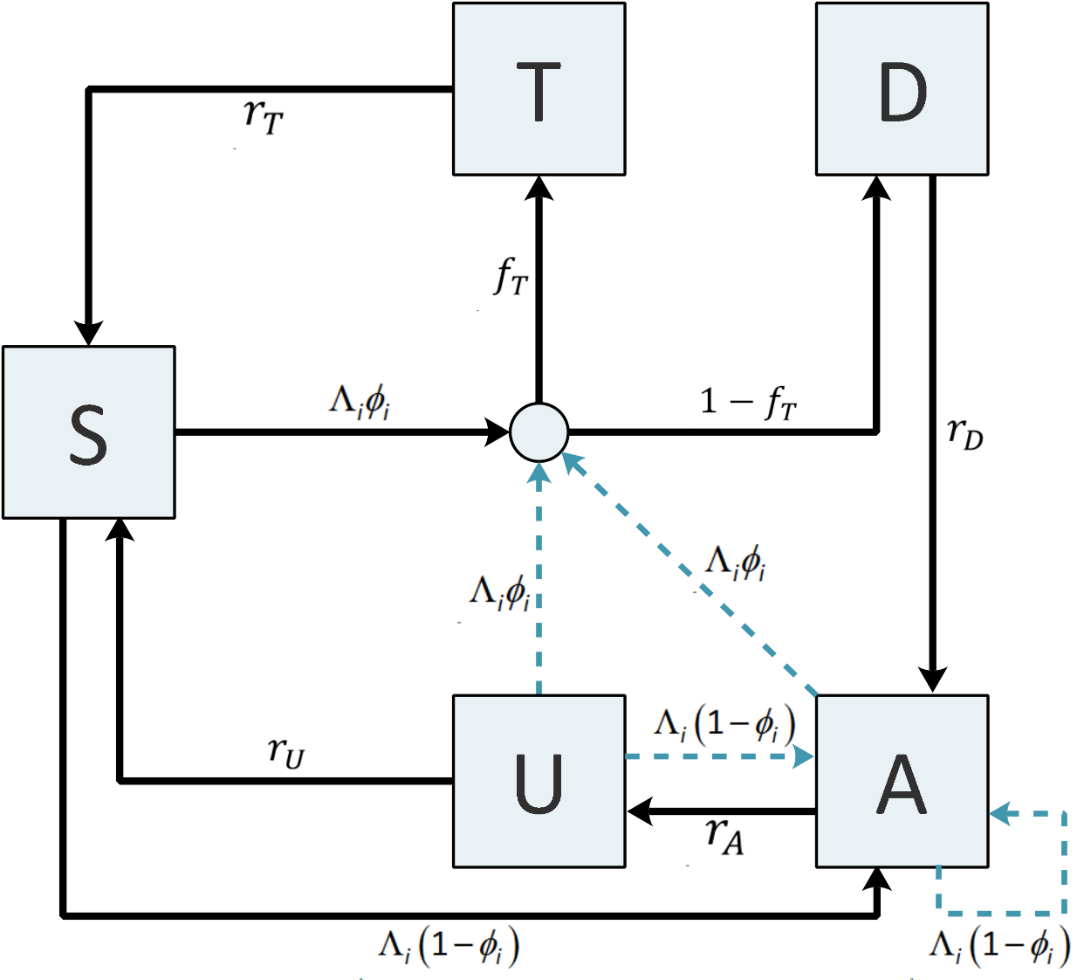


***Figure S1****. Diagram of the human model of malaria transmission was previously published by Winskill et.al ^27^. In the model, an infected human moves between states, represented by boxes, at associated hazard rates, represented by arrows. Dashed arrows represent superinfection. The circle represents the treatment node. Note: S = susceptible; T = treated clinical disease; D = untreated clinical disease; A = asymptomatic patent infection; U = asymptomatic sub-patent infection.*

***Table S1****. Transition rates between human infection states.*

| **Process** | **Transition** | **Rate** |
| --- | --- | --- |
| Infection resulting in treated clinical disease | S 🡪 T | $\Lambda_{i} (t-d_{E})\phi_{i}f_{T}$ |
| Infection resulting in untreated clinical disease | S 🡪 D | $\Lambda_{i} (t-d_{E})\phi_{i}(1-f_{T})$ |
| Infection resulting in asymptomatic infection | S 🡪 A | $\Lambda_{i} (t-d_{E})(1-\phi_{i})$ |
| Progression of untreated disease to asymptomatic infection | D 🡪 A | $r_{D}=\frac{1}{d_{D}}$ |
| Progression of asymptomatic infection to sub-patent infection | A 🡪 U | $r_{A}=\frac{1}{d_{A}}$ |
| Progression of sub-patent infection to susceptible | U 🡪 S | $r_{U}=\frac{1}{d_{U}}$ |
| Progression of treated disease to susceptible (treated individuals experience a period of drug-dependent partial protection from reinfection) | T 🡪 S | $r_{T}=\frac{1}{d_{T}}$ |
| Super-infection from asymptomatic or sub-patent infection to treated clinical disease | A 🡪 T  U 🡪 T | $\Lambda_{i}(t-d_{E})\phi_{i}f_{T}$ |
| Super-infection from asymptomatic or sub-patent infection to untreated clinical disease | A 🡪 D  U 🡪 D | $\Lambda_{i} (t-d_{E})\phi_{i}(1-f_{T})$ |
| Super-infection from asymptomatic or sub-patent infection to treated asymptomatic infection | A 🡪 A  U 🡪 A | $\Lambda_{i} (t-d_{E})(1-\phi_{i})$ |

## Immunity

The human component models multiple types of immunity.

Infants are born with maternal immunity, representing placental transfer of antibodies, to clinical disease and severe disease which are set to proportions $P_{CM}$ and $P_{VM}$ of the immunity in a population aged 15-35 years that has the same biting rate as the infant. Maternally acquired immunity reduces the probability of clinical or severe disease given an infection and wanes exponentially at rate $r_{M}$.

$$r_{M}=\frac{1}{d_{M}}$$

Pre-erythrocytic immunity is gradually acquired with age and exposure to malaria. It is modelled as a reduction in the probability of developing a patent infection and is boosted after each infectious bite, if the last exposure was at least $\mu_{B}$ days previously, then wanes exponentially at rate $r_{B}$**.**

$$r_{B}=\frac{1}{d_{B}}$$

Blood stage immunity against severe disease $I_{VA}$, clinical disease $I_{CA}$, and detectability of asymptomatic infection $I_{D}$is boosted after each patent infection if the last patent, or detectable, infection was at least $\mu_{V}$, $\mu_{C}$ or $\mu_{D}$days, respectively, ago, and then wanes exponentially following each malaria infection at rates $r_{VA}=1/d_{VA}$, $r_{CA}=1/d_{CA}$ and $r_{ID}=1/d_{ID}$, for severe disease, clinical disease, and detectability of asymptomatic infection, respectively. Immunity against the detectability of asymptomatic infection reduces the probability of detection and infectiousness to mosquitoes.

The probability of developing clinical disease after infection is

$$\phi_{i}\left( t \right)=\phi_{0}\left( \phi_{1}+\frac{1-\phi_{1}}{1+\left( \frac{I_{CA}\left( i,t \right)+I_{CM}(i,t)}{I_{C0}} \right)^{\kappa_{C}}} \right)$$

where individual $i$ at time $t$ has a baseline probability ($\phi_{0}$) of developing clinical disease assuming no immunity, and ${\phi_{0}\phi}_{1}$is the minimum probability of developing clinical disease. $I_{C0}$ is a scale parameter and $\kappa_{C}$ is a shape parameter, and $I_{CA}\left( i,t \right)$ and $I_{CM}(i,t)$ are the levels of acquired and maternally acquired immunity to clinical disease, respectively.

The probability that an individual develops severe disease depends on blood-stage and maternal immunity against severe disease and is described by

$$\theta_{i}\left( t \right)=\theta_{0}\left( \theta_{1}+\frac{1-\theta_{1}}{1+f_{V}(i,a)\left( \frac{I_{VA}\left( i,t \right)+I_{VM}(i,t)}{I_{V0}} \right)^{\kappa_{V}}} \right)$$

where individual $i$ at time $t$ has a baseline probability ($\theta_{0}$) of developing severe disease assuming no immunity and $\theta_{0}\theta_{1}$is the minimum probability. $I_{V0}$ is a scale parameter and $\kappa_{V}$ is a shape parameter, and $I_{VA}\left( i,t \right)$ and $I_{VM}(i,t)$ are the levels of acquired and maternally acquired immunity to severe disease, respectively. $f_{V}(i,a)$ modifies the risk of severe disease by age.

$$f_{V}\left( i,a \right)=1-\frac{(1-f_{V0})}{(1+\left( \frac{a}{a_{v}} \right)^{\gamma_{V}})}$$

Treated individuals with clinical disease have a reduced probability of developing severe disease, $f_{VT}$.

Blood-stage immunity reduces the probability of detection by microscopy of infection if asymptomatic and also reduces the infectiousness of humans to mosquitoes since blood-stage immunity results in lower parasitaemia.

Severe disease is calculated by taking a proportion of individuals with clinical disease and then scaling this by the level of treatment coverage ^31^.

## Vector model

The mosquito component of the model simulates varying levels of human infectivity to mosquitoes, with a lower chance of a mosquito contracting malaria from a subpatent compared to patent human infection. Juvenile mosquitoes pass through early and late larval states, during which there are state-specific mortality rates depending on the carrying capacity ($K$), the pupal stage, and then become adult mosquitoes of which 50% are assumed to be female and all are assumed to be susceptible upon emergence from the pupal stage. Adult female mosquitoes can be susceptible, incubating, or infectious.

## Seasonality

Seasonality is modelled by changing the carrying capacity
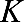
 for mosquito larvae of the environment over time $t$


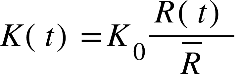


where $K_{0}$ is the carrying capacity at time 0,
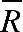
 is the mean annual rainfall, and $R\left( t \right)$is a seasonal curve that changes over time. $R\left( t \right)$is estimated using the first three frequencies of a Fourier transform fitted to estimates of rainfall from CHIRPS ^32^.


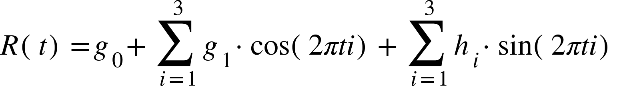


Where $g_{0}, g_{1}$, $g_{2}$, $g_{3}$, $h_{1}$, $h_{2}$, and $h_{3}$ are fitted parameters. We modelled seasonal and perennial settings from rainfall data in locations in Central and West Africa, respectively, from the {umbrella} R package ^33^. The location-specific rainfall data were Fourier transformed to adjust mosquito carrying capacity in the model.

## Interventions

We assumed that baseline prevalence of *P. falciparum* in children aged 2-10 years (*Pf*PR_2-10_) at the beginning of the simulation incorporated existing usage of malaria control interventions (such as insecticide-treated bed nets, seasonal malaria chemoprevention, indoor residual spraying, etc.), and so these interventions were not modelled explicitly. However, we did model a constant level of treatment of an infected individual as well as vaccination with the R21/Matrix-M vaccine.

### Treatment

Successful treatment of an infected individual in the model returns that person to the susceptible class, while retaining a degree of partial immunity depending on the level of the drug. We assumed treatment with artemether-lumefantrine (AL), with a 95% probability of clearing the infection. This was estimated using a pharmacokinetic-pharmacodynamic model fitted to clinical trial data from 6 different sites in sub-Saharan Africa ^29^. $P_{T}\left( \mu\right)$represents the short-lived post-treatment protection from infection at time $\mu$, and the probability of re-infection relative to no treatment is multiplied by $1-P_{T}(\mu)$. The degree of protection $A_{T}$ for an individual is estimated as the area under the curve. For AL, the short-lived post-treatment protection $A_{T}$ varies by age from 7 to 16 days.


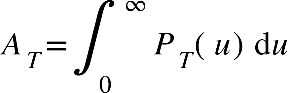


### Vaccination

We incorporated the median values of published estimates of antibody and vaccine efficacy parameters for the R21/Matrix-M vaccine into our model. A biphasic exponential decay model was fit to anti-circumsporozoite protein (anti-CSP) antibody titre data from the Phase II trial for R21/Matrix-M to estimate antibody parameters ^22,34^. We assumed that titres peak ($CSP_{peak}$) after the three primary doses of R21/Matrix-M and decline over time with short-lived $r_{s}$ and long-lived $r_{l}$ decay rates with half-lives of decay rates represented as $d_{s}$ and $d_{l}$.


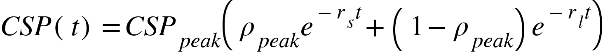


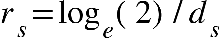


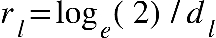


The proportion of the response that is short-lived and long-lived is represented by $\rho_{peak}$ and $1-\rho_{peak}$, respectively.

After a booster dose delivered at time $t_{boost}$, the titres will again peak and decay over time at the same rates as above. However, the proportion of the response generated by short-lived cells may change $\rho_{boost}$. The change in antibody dynamics over time is described by this equation:


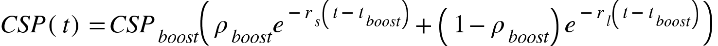


These modelled anti-CSP antibody titres were related to vaccine efficacy against infection over time $V\left( t \right)$ with a Hill function dose-response curve:


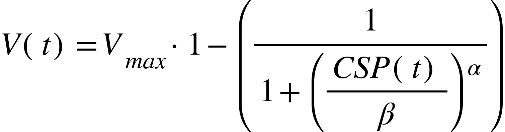


Where $V_{max}$ is the maximum efficacy, and $\alpha$ and $\beta$ are shape and scale parameters.

The vaccine efficacy parameters were estimated by fitting to data on clinical malaria incidence from the Phase II R21/Matrix-M trial ^34^ and are shown in Table S3.

## Model parameter values

**Table S2.** Default parameter values for the *malariasimulation* model

| Parameter | | Model parameter | | Definition | Estimate |
| --- | --- | --- | --- | --- | --- |
| Fixed state transitions (days) | | | | | |
| $\boldsymbol{d}_{\boldsymbol{D}}\mathbf{=}\frac{\mathbf{1}}{\boldsymbol{r}_{\boldsymbol{D}}}$ | | **dd** | | Length of untreated clinical disease | 5 days |
| $\boldsymbol{d}_{\boldsymbol{T}}\mathbf{=}\frac{\mathbf{1}}{\boldsymbol{r}_{\boldsymbol{T}}}$ | | **dt** | | Length of treated clinical disease | 5 days |
| $\boldsymbol{d}_{\boldsymbol{A}}\mathbf{=}\frac{\mathbf{1}}{\boldsymbol{r}_{\boldsymbol{A}}}$ | | **da** | | Length of patent infection | 195 days |
| $\boldsymbol{d}_{\boldsymbol{U}}\mathbf{=}\frac{\mathbf{1}}{\boldsymbol{r}_{\boldsymbol{U}}}$ | | **du** | | Sub-patent infection | 110 days |
| Mosquito model | | | | | |
| $\boldsymbol{d}_{\boldsymbol{E}}$ | | **del** | | delay of mosquito movement from state E to L (early instar larval developmental period) | 6.64 days |
| $\boldsymbol{d}_{\boldsymbol{L}}$ | | **dl** | | delay of mosquito movement from state L to P (late instar larval development period) | 3.72 days |
| $\boldsymbol{d}_{\boldsymbol{P}}$ | | **dpl** | | delay of mosquito movement from state P to S_m_ (pupal development period) | 0.643 days |
| Immunity decay (days) | | | | | |
| $\boldsymbol{d}_{\boldsymbol{M}}$ | | **rm** | | inverse of decay rate for maternal immunity to clinical disease | 67.6952 |
| $\boldsymbol{d}_{\boldsymbol{VM}}$ | | **rvm** | | inverse of decay rate for maternal immunity to severe disease | 76.8365 |
| $\boldsymbol{d}_{\boldsymbol{B}}$ | | **rb** | | inverse of decay rate for acquired pre-erythrocytic immunity | 3650 |
| $\boldsymbol{d}_{\boldsymbol{CA}}$ | | **rc** | | inverse of decay rate for acquired immunity to clinical disease | 10950 |
| $\boldsymbol{d}_{\boldsymbol{VA}}$ | | **rva** | | inverse of decay rate for acquired immunity to severe disease | 10950 |
| $\boldsymbol{d}_{\boldsymbol{ID}}$ | | **rid** | | inverse of decay rate for acquired immunity to detectability | 3650 |
| Pre-erythrocytic infection | | | | | |
| $\boldsymbol{b}_{\boldsymbol{0}}$ | | **b0** | | maximum probability due to no immunity | 0.59 |
| $\boldsymbol{b}_{\boldsymbol{1}}$ | | **b1** | | maximum reduction due to immunity | 0.5 |
| $\boldsymbol{I}_{\boldsymbol{B}\boldsymbol{0}}$ | | **ib0** | | scale parameter | 43.9 |
| $\boldsymbol{\kappa}_{\boldsymbol{B}}$ | | **kb** | | shape parameter | 2.16 |
| Clinical infection | | | | | |
| $\boldsymbol{\phi}_{\boldsymbol{0}}$ | | **phi0** | | maximum probability due to no immunity | 0.791666 |
| $\boldsymbol{\phi}_{\boldsymbol{1}}$ | | **phi1** | | maximum reduction due to immunity | 0.000737 |
| $\boldsymbol{I}_{\boldsymbol{C}\boldsymbol{0}}$ | | **ic0** | | scale parameter | 18.02366 |
| $\boldsymbol{\kappa}_{\boldsymbol{C}}$ | | **kc** | | shape parameter | 2.36949 |
| Severe disease | | | | | |
| $\boldsymbol{\theta}_{\boldsymbol{0}}$ | | **theta0** | | maximum probability due to no immunity | 0.0749886 |
| $\boldsymbol{\theta}_{\boldsymbol{1}}$ | | **theta1** | | maximum reduction due to immunity | 0.0001191 |
| $\boldsymbol{\kappa}_{\boldsymbol{V}}$ | | **kv** | | shape parameter | 2.00048 |
| $\boldsymbol{I}_{\boldsymbol{V}\boldsymbol{0}}$ | | **iv0** | | scale parameter | 1.09629 |
| $\boldsymbol{f}_{\boldsymbol{V}\boldsymbol{0}}$ | | **fv0** | | age dependent modifier | 0.141195 |
| $\boldsymbol{a}_{\boldsymbol{V}}$ | | **av** | | age dependent modifier | 2493.41 |
| $\boldsymbol{\gamma}_{\boldsymbol{V}}$ | | **gammav** | | age dependent modifier | 2.91282 |
| Immunity reducing probability of detection | | | | | |
| $\boldsymbol{f}_{\boldsymbol{D}\boldsymbol{0}}$ | | **fd0** | | time-scale at which immunity changes with age | 0.007055 |
| $\boldsymbol{a}_{\boldsymbol{D}}$ | | **ad** | | scale parameter relating age to immunity | 7993.5 |
| $\boldsymbol{\gamma}_{\boldsymbol{D}}$ | | **gammad** | | shape parameter relating age to immunity | 4.8183 |
| $\boldsymbol{d}_{\boldsymbol{1}}$ | | **d1** | | minimum probability due to immunity | 0.160527 |
| $\boldsymbol{I}_{\boldsymbol{D}\boldsymbol{0}}$ | | **id0** | | scale parameter | 1.577533 |
| $\boldsymbol{\kappa}_{\boldsymbol{D}}$ | | **kd** | | shape parameter | 0.476614 |
| Immunity boost grace periods (days) | | | | | |
| $\boldsymbol{u}_{\boldsymbol{B}}$ | | **ub** | | period in which pre-erythrocytic immunity is not boosted | 7.19919 |
| $\boldsymbol{u}_{\boldsymbol{C}}$ | | **uc** | | period in which clinical immunity is not boosted | 6.06 |
| $\boldsymbol{u}_{\boldsymbol{V}}$ | | **uv** | | period in which severe immunity is not boosted | 11.4321 |
| $\boldsymbol{u}_{\boldsymbol{D}}$ | | **ud** | | period in which immunity to detectability is not boosted | 9.44512 |
| Infectivity towards mosquitos | | | | | |
| $\boldsymbol{c}_{\boldsymbol{D}}$ | | **cd** | | infectivity of untreated clinical disease | 0.068 |
| $\boldsymbol{\gamma}_{\boldsymbol{1}}$ | | **gamma1** | | infectivity of asymptomatic infection | 1.82425 |
| $\boldsymbol{c}_{\boldsymbol{U}}$ | | **cu** | | infectivity of sub-patent infection | 0.0062 |
| $\boldsymbol{c}_{\boldsymbol{T}}$ | | **ct** | | infectivity of treated clinical disease | 0.021896 |
| Biting rate | | | | | |
| $\boldsymbol{\sigma}^{\boldsymbol{2}}$ | | **sigma_squared** | | Variance of the log heterogeneity in biting rates | 1.67 |
|  | | **n_heterogeneity_groups** | | number of discrete groups for heterogeneity in exposure to mosquito bites | 5 |
| $\boldsymbol{a}_{\boldsymbol{0}}$ | | **a0** | | age dependent biting parameter | 2920 days |
| $\boldsymbol{\rho}$ | | **rho** | | age dependent biting parameter | 0.85 |
| Maternal immunity parameters | | | | | |
| $\boldsymbol{P}_{\boldsymbol{CM}}$ | | **pcm** | | newborn clinical immunity relative to mother's | 0.774368 |
| $\boldsymbol{P}_{\boldsymbol{VM}}$ | | **pvm** | | newborn severe immunity relative to mother's | 0.195768 |
| Mosquito mortality parameters | | | | | |
| $\boldsymbol{\mu}_{\boldsymbol{E}}$ | | **me** | | early-stage larval mortality rate | 0.0338 |
| $\boldsymbol{\mu}_{\boldsymbol{L}}$ | | **ml** | | late-stage larval mortality rate | 0.0348 |
| $\boldsymbol{\mu}_{\boldsymbol{P}}$ | | **mup** | | the rate at which pupal mosquitos die | 0.249 |
| $\boldsymbol{\mu}_{\boldsymbol{M}}$ | | **mum** | | the rate at which developed mosquitos die | 0.1253333 |
| Seasonality | | | | | |
| $\boldsymbol{g}_{\boldsymbol{0}}$ | | **g0** | | rainfall Fourier parameter | seasonal: 0.2855050  perennial: 0.2852770 |
| $\boldsymbol{g}$ | | **g** | | rainfall Fourier parameter | seasonal: -0.325352,  -0.0109352, 0.0779865  perennial: -0.0248801, -0.0529426,  -0.0168910 |
| $\boldsymbol{h}$ | | **h** | | rainfall Fourier parameters | seasonal: -0.132815, 0.104675, -0.013919  perennial: -0.0216681, -0.0242904, -0.0073646 |
| $\boldsymbol{\gamma}$ | | **gamma** | | effect of density dependence on late instars relative to early instars | 13.25 |
|  | | **rainfall_floor** | | the minimum rainfall value | 0.001 |
| Incubation periods (days) | | | | | |
| $\boldsymbol{d}_{\boldsymbol{E}}$ | | **de** | | duration of the human latent period of infection | 12 |
| $\boldsymbol{d}_{\boldsymbol{g}}$ | | **delay_gam** | | lag from parasites to infectious gametocytes | 12.5 |
| $\boldsymbol{d}_{\boldsymbol{EM}}$ | | **dem** | | extrinsic incubation period in mosquito population model | 10 |
| Vector biology | | | | | |
| $\boldsymbol{\beta}$ | | **beta** | | average number of eggs laid per female mosquito per day | 21.2/day |
| 1 / $\boldsymbol{\delta}$ | | **blood_meal_rates** | | Inverse of mean time between blood meal feeds | 3 days |
| $\boldsymbol{Q}_{\boldsymbol{0}}$ | | **Q0** | | proportion of blood meals taken on humans | *A. arabiensis*: 0.71  *A. funestus*: 0.94  *A. gambiae*: 0.92 |
|  | | **foraging_time** | | time spent taking blood meals | 0.69 |
|  | | **species_proportions** | | Proportion of each mosquito species | *A. arabiensis*: 0.25  *A. funestus*: 0.25  *A. gambiae*: 0.5 |
| Treatment | | | | | |
| $\boldsymbol{P}_{\boldsymbol{T}}$ | | **drug_efficacy** | | a vector of efficacies for available drugs | AL: 0.95 |
|  | | **drug_rel_c** | | a vector of relative onwards infectiousness values for drugs | AL: 0.05094 |
|  | | **drug_prophylaxis_shape** | | a vector of shape parameters for Weibull curves to model prophylaxis for each drug | AL: 11.3 |
|  | | **drug_prophylaxis_scale** | | a vector of scale parameters for Weibull curves to model prophylaxis for each drug | AL: 10.6 |
| $\boldsymbol{f}_{\boldsymbol{T}}$ | | **clinical_treatment_coverages** | | a vector of coverage values for each drug | AL: 0.45 |
| R21 primary series | | | | | |
| $\boldsymbol{V}_{\boldsymbol{max}}$ | | **vmax** | | the maximum efficacy of the vaccine | 0.87 |
| $\boldsymbol{\alpha}$ | | **alpha** | | shape parameter for the vaccine efficacy model | 0.91 |
| $\boldsymbol{\beta}$ | | **beta** | | scale parameter for the vaccine efficacy model | 471 |
| $\boldsymbol{CSP}_{\boldsymbol{peak}}$ | | **cs** | | peak parameters for the antibody model (mean, std. dev) | 9.320, 0.839 |
| $\boldsymbol{\rho}_{\boldsymbol{peak}}$ | | **rho** | | delay parameters for the antibody model (mean, std. dev) | 0.807, 0.601 |
| $\boldsymbol{d}_{\boldsymbol{s}}$ | | **ds** | | delay parameters for the antibody model, short-term weaning (mean, std. dev) | 3.800, 0.162 |
| $\boldsymbol{d}_{\boldsymbol{l}}$ | | **dl** | | delay parameters for the antibody model, long-term weaning (mean, std. dev) | 6.282, 0.455 |
| R21 first booster dose | | | | | |
| $\boldsymbol{V}_{\boldsymbol{max}}$ | | **vmax** | | the maximum efficacy of the vaccine | 0.87 |
| $\boldsymbol{\alpha}$ | | **alpha** | | shape parameter for the vaccine efficacy model | 0.91 |
| $\boldsymbol{\beta}$ | | **beta** | | scale parameter for the vaccine efficacy model | 471 |
| $\boldsymbol{CSP}_{\boldsymbol{peak}}$ | | **cs** | | peak parameters for the antibody model (mean, std. dev) | 9.237, 0.719 |
| $\boldsymbol{\rho}_{\boldsymbol{peak}}$ | | **rho** | | delay parameters for the antibody model (mean, std. dev) | 0.0714, 0.5418 |
| $\boldsymbol{d}_{\boldsymbol{s}}$ | | **ds** | | delay parameters for the antibody model, short-term weaning (mean, std. dev) | 3.800, 0.162 |
| $\boldsymbol{d}_{\boldsymbol{l}}$ | | **dl** | | delay parameters for the antibody model, long-term weaning (mean, std. dev) | 6.282, 0.455 |
| R21 second and third booster doses | | | | | |
| $\boldsymbol{V}_{\boldsymbol{max}}$ | | **vmax** | | the maximum efficacy of the vaccine | 0.87 |
| $\boldsymbol{\alpha}$ | | **alpha** | | shape parameter for the vaccine efficacy model | 0.91 |
| $\boldsymbol{\beta}$ | | **beta** | | scale parameter for the vaccine efficacy model | 471 |
| $\boldsymbol{CSP}_{\boldsymbol{peak}}$ | | **cs** | | peak parameters for the antibody model (mean, std. dev) | 9.02, 0.845 |
| $\boldsymbol{\rho}_{\boldsymbol{peak}}$ | | **rho** | | delay parameters for the antibody model (mean, std. dev) | 0.807, 0.601 |
| $\boldsymbol{d}_{\boldsymbol{s}}$ | | **ds** | | delay parameters for the antibody model, short-term weaning (mean, std. dev) | 3.800, 0.162 |
| $\boldsymbol{d}_{\boldsymbol{l}}$ | | **dl** | | delay parameters for the antibody model, long-term weaning (mean, std. dev) | 6.282, 0.455 |
| Demography | | | | | |
|  | **deathrate_agegroups** | | vector of age groups (days) | | See <https://github.com/kellymccain28/catchup_extraboosters/blob/main/src/1_create_parameter_list/ssa_demography_2021.csv> |
|  | **deathrates** | | deathrates per age group | |  |

**Table S3**. Model assumptions and references

| **Assumption** | **Reference** |
| --- | --- |
| **Demographic age structure**  UNWPP Africa region demography and African neonatal mortality from UNICEF | ^30^ |
| **Vaccine coverage**  1^st^, 2^nd^, 3^rd^ doses: 80%  1^st^ booster dose: 64%  Extra booster doses: 64% | RTS,S/AS01 MVIP ^3^ |
| **Seasonality settings**  Perennial and seasonal archetypal profiles from rainfall data in locations in Central and West Africa, respectively, that were Fourier transformed to adjust mosquito carrying capacity in the model | ^33^ |
| **Coverage of effective treatment of clinical malaria cases**  45% | ^21^ |
| **Vaccine efficacy parameters**  Estimated from fitting a biphasic exponential antibody decay model of antibody titres over time, relating subsequently to vaccine efficacy over time using a Hill function dose-response curve | ^22,34,35^ |

# Supplementary Results

***
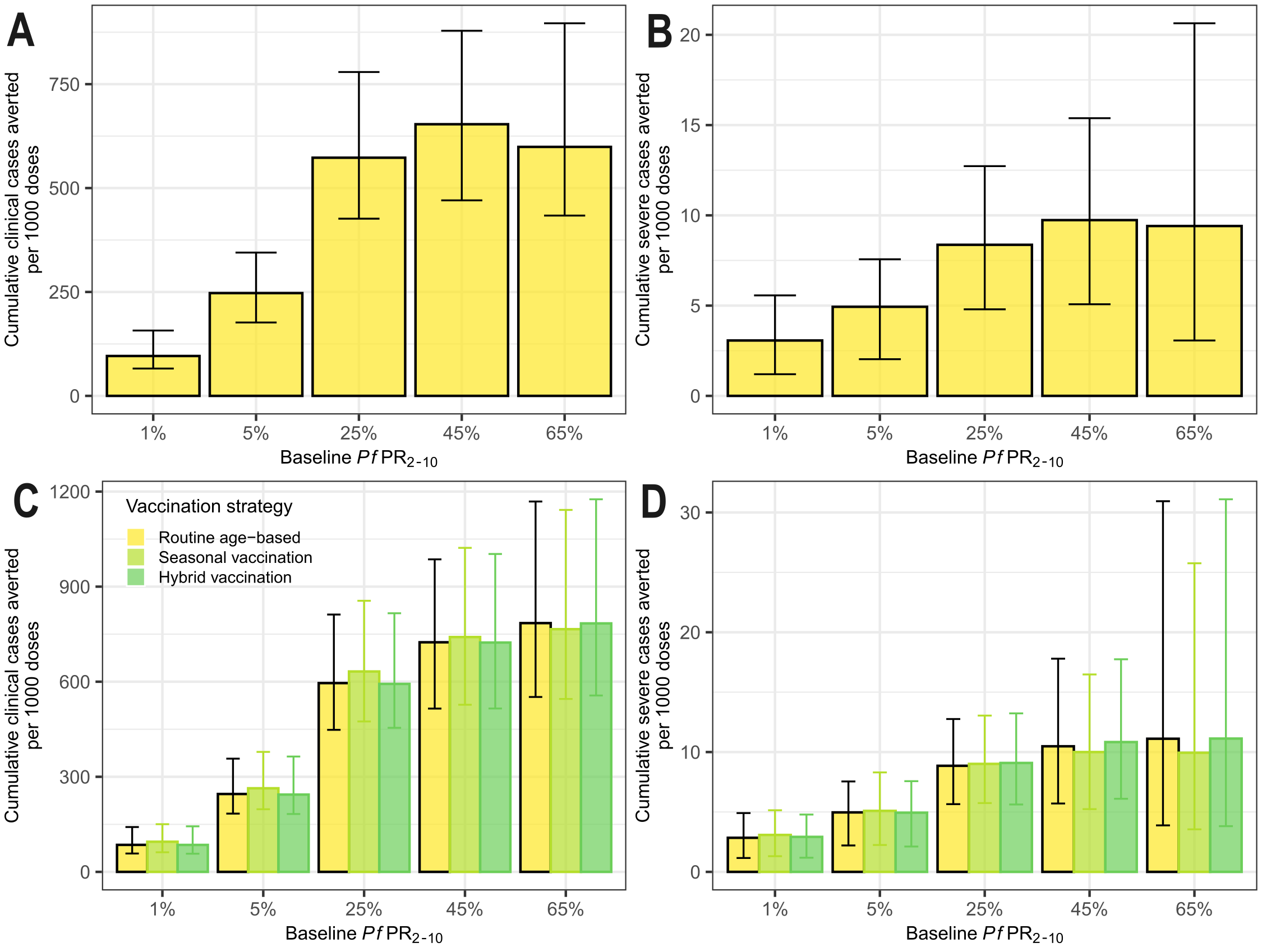
Figure S2. Cumulative clinical and severe cases averted per 1000 doses over the 30-year simulation with age-based, seasonal, and hybrid implementation in perennial settings (A and B) and seasonal settings (C and D).*** *Age-based vaccination is delivered with three primary doses at the beginning of the simulation and a booster dose 1 year later. In seasonal implementation, the three primary doses are delivered at 5.5, 4.5, and 3.5 months before the seasonal peak in clinical incidence. The hybrid strategy times the three primary doses according to the age-based schedule, with the booster dose timed to be 3.5 months before the next seasonal peak in clinical incidence about a year later. The bars show median values, and the error bars show 95% credible intervals of 50 stochastic parameter draws. Note that the y-axes in plots A and B are different.*

***
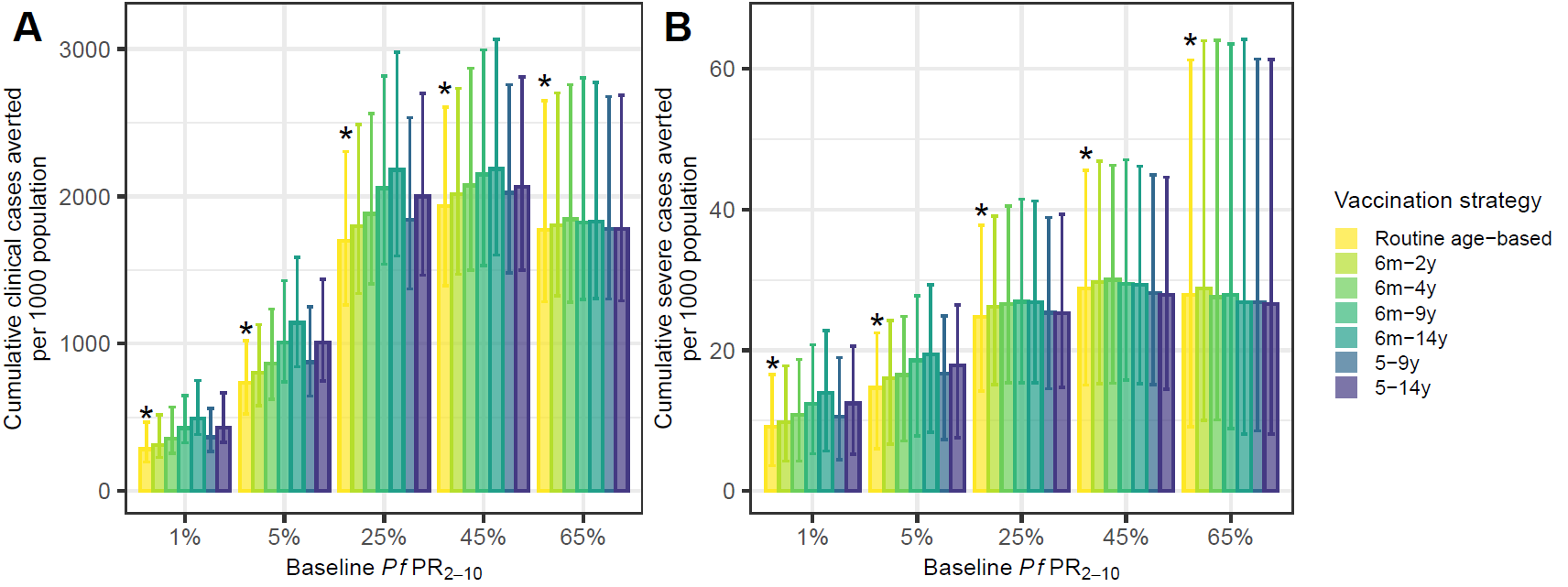
Figure S3. Catch-up campaign impact per 1000 people, perennial setting: cumulative clinical (A) and severe (B) cases averted per 1000 population in perennial settings under different vaccination strategies over a 30-year simulation.*** *Age-based routine vaccination in 6-month-olds with supplementary catch-up vaccination in older children is assumed in all catch-up scenarios. The number of clinical or severe cases is compared to a no-vaccination baseline. The bars show median values, and the error bars show 95% credible intervals of 50 stochastic model runs.*


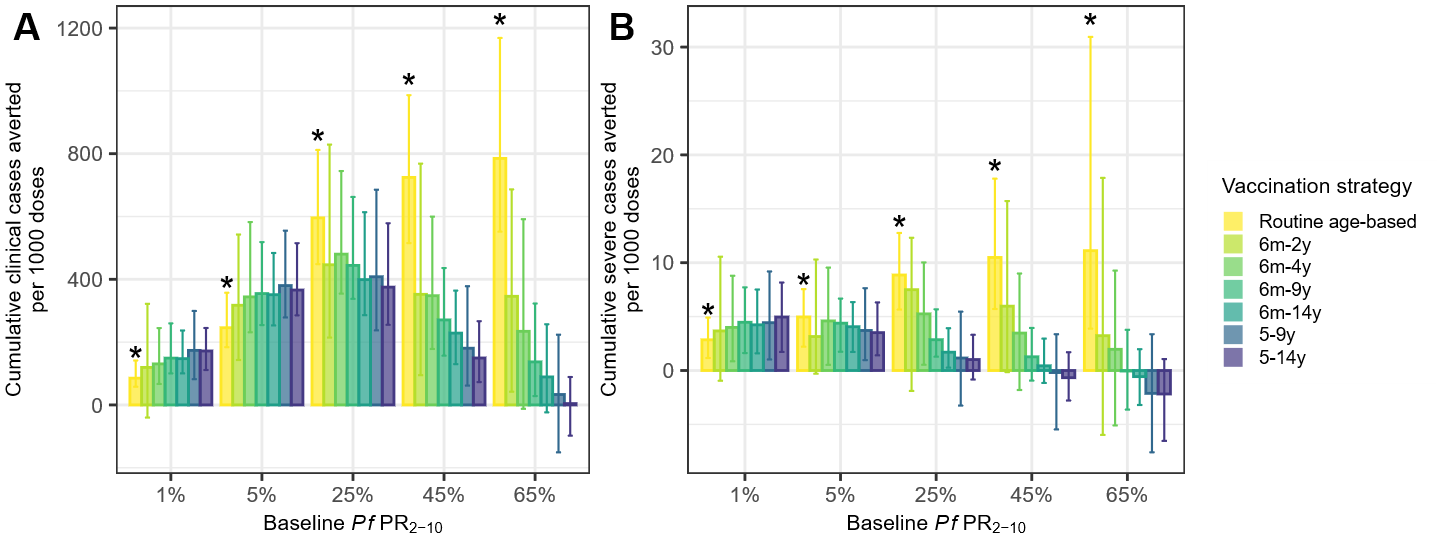


***Figure S4. Catch-up campaign impact per 1000 doses, seasonal setting: cumulative clinical (A) and severe (B) cases averted per 1000 doses in a 30-year simulation post-vaccination in seasonal settings.*** *Values for routine age-based vaccination (bars with * above) show the outcome per 1000 doses delivered relative to no vaccination, while all other plotted strategies show additional outcomes averted per additional doses delivered relative to routine age-based vaccination. Age-based routine vaccination in 6-month-olds with supplementary catch-up vaccination in older children is assumed in all catch-up scenarios. The number of clinical or severe cases is compared to the routine age-based scenario baseline. The number of doses is calculated as the total number of doses under the specified vaccination strategy compared to a baseline scenario of routine age-based vaccination. The bars show median values, and the error bars show 95% credible intervals from 50 stochastic model runs. Note that the y-axes in plots A and B are different.*

***
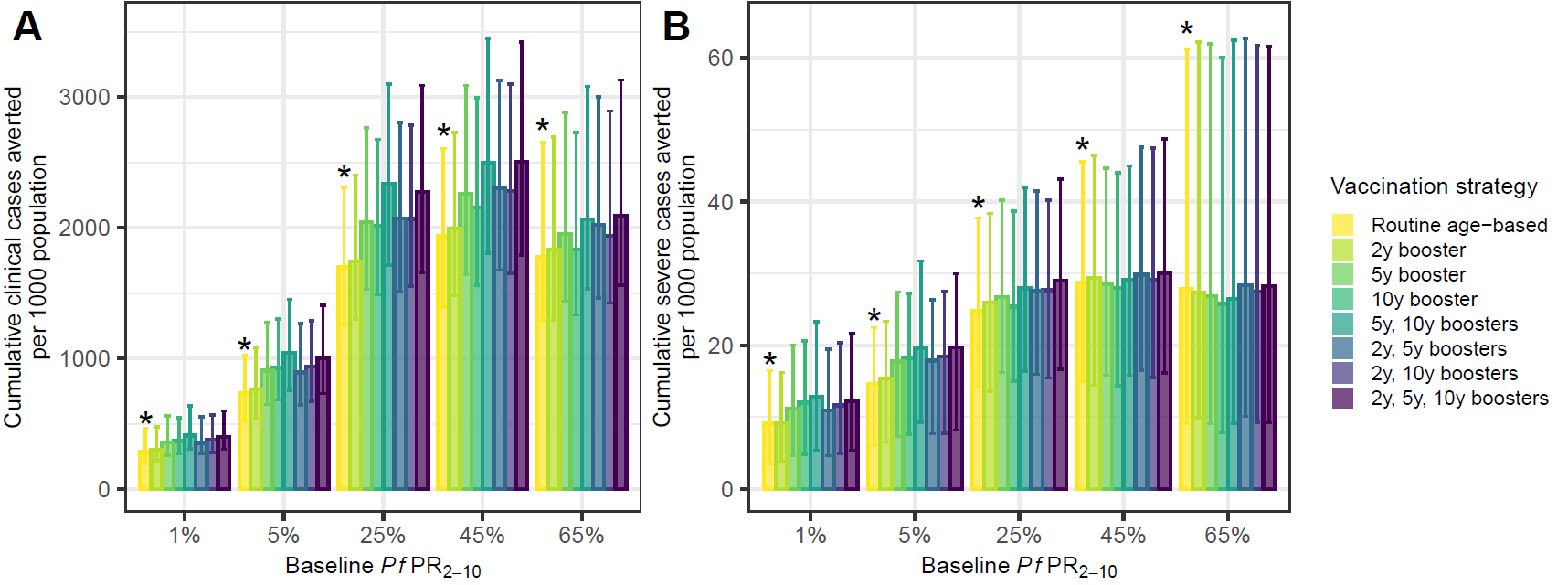
Figure S5. Extra booster impact per 1000 people, perennial setting: Cumulative clinical (A) and severe (B) cases averted per 1000 people over the last 15 years of the simulation in perennial settings.*** *Age-based routine vaccination in 6-month-olds is assumed in all scenarios; extra booster doses were delivered to previously vaccinated children. The number of clinical or severe cases is compared to a no-vaccination baseline. The bars show median values, and the error bars show 95% credible intervals of 50 stochastic model runs. Note that the y-axes in plots A and B are different.*

***
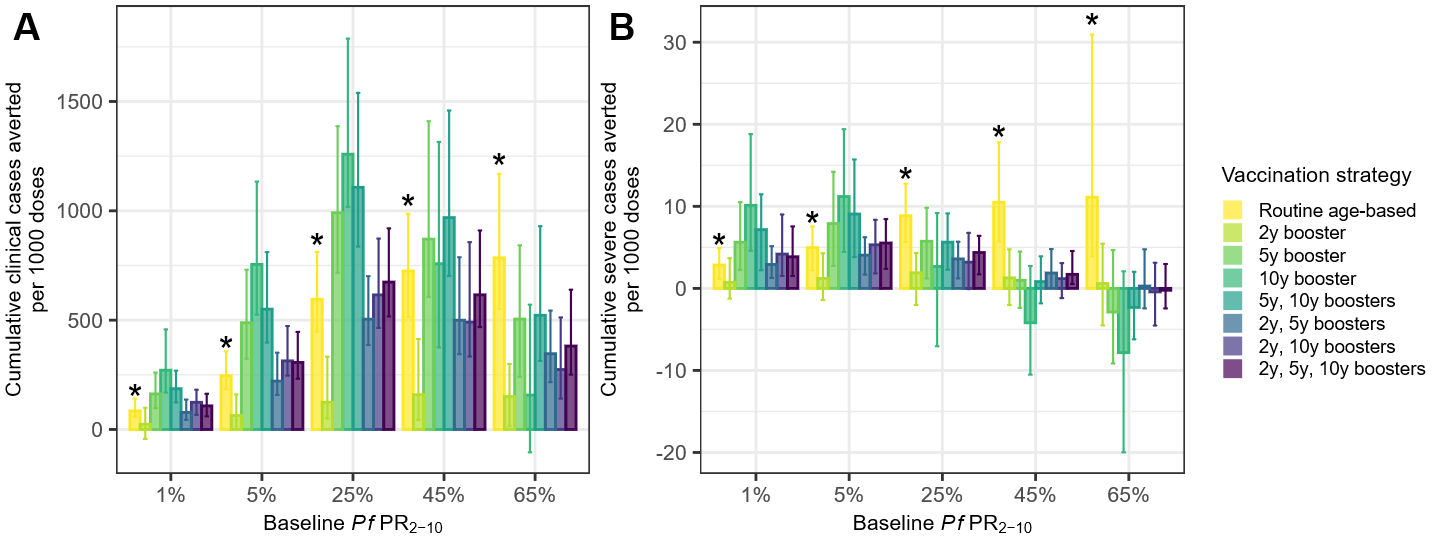
Figure S6. Extra booster impact per 1000 doses, seasonal setting: Cumulative clinical cases (A) and severe cases (B) averted per 1000 additional doses in perennial settings.*** *Values for routine age-based vaccination (bars with * above) show the outcome per 1000 doses delivered relative to no vaccination, while all other plotted strategies show additional outcomes averted per additional doses delivered relative to routine age-based vaccination. Outcomes are compared to a routine age-based vaccination baseline and are summarised over the final 15 years of the simulation so that each scenario had 15 years of continuous vaccination to young children before calculating cases averted. This allows for a fairer comparison between strategies with different booster dose timing. The bars show median values, and the error bars show 95% credible intervals of 50 stochastic model runs. Note that the y-axes in plots A and B are different.*

***
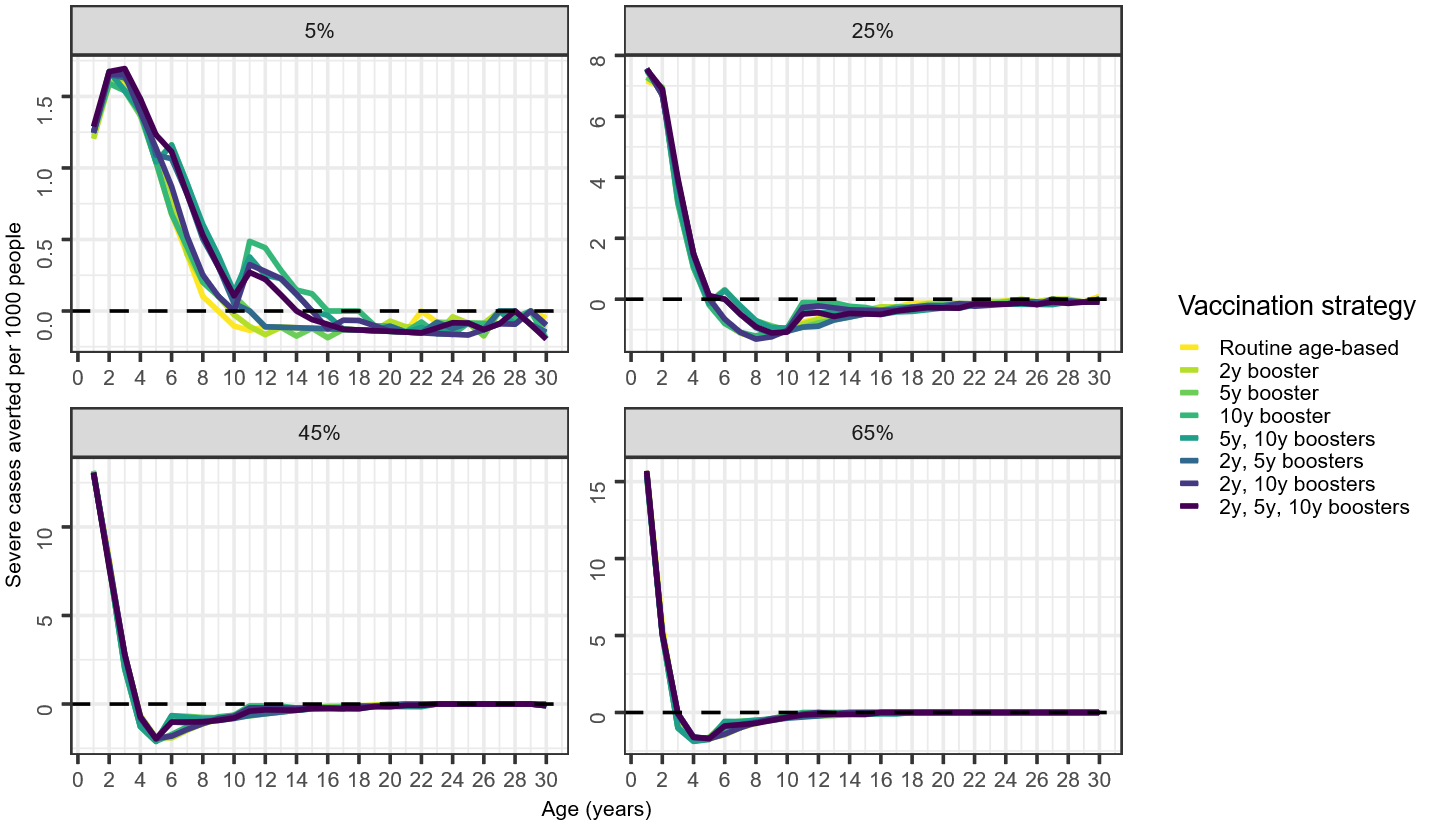
Figure S7. Extra booster impact: Severe cases averted per 1000 people in each age group compared to a baseline scenario with no vaccination in a perennial setting.*** *All individuals were vaccinated with routine age-based vaccination at 6 months of age with a booster 1 year later and were followed over 30 years. Depending on the modelled strategy, individuals were vaccinated with up to 3 extra booster doses at combinations of 2, 5, and/or 10 years post primary series. Severe cases averted were calculated by dividing the severe cases averted for each age group (relative to a scenario with no vaccination) by the total number of people in that age group. The coloured lines are the median values, and the shaded regions are 95% credible intervals over 50 stochastic draws.*

***
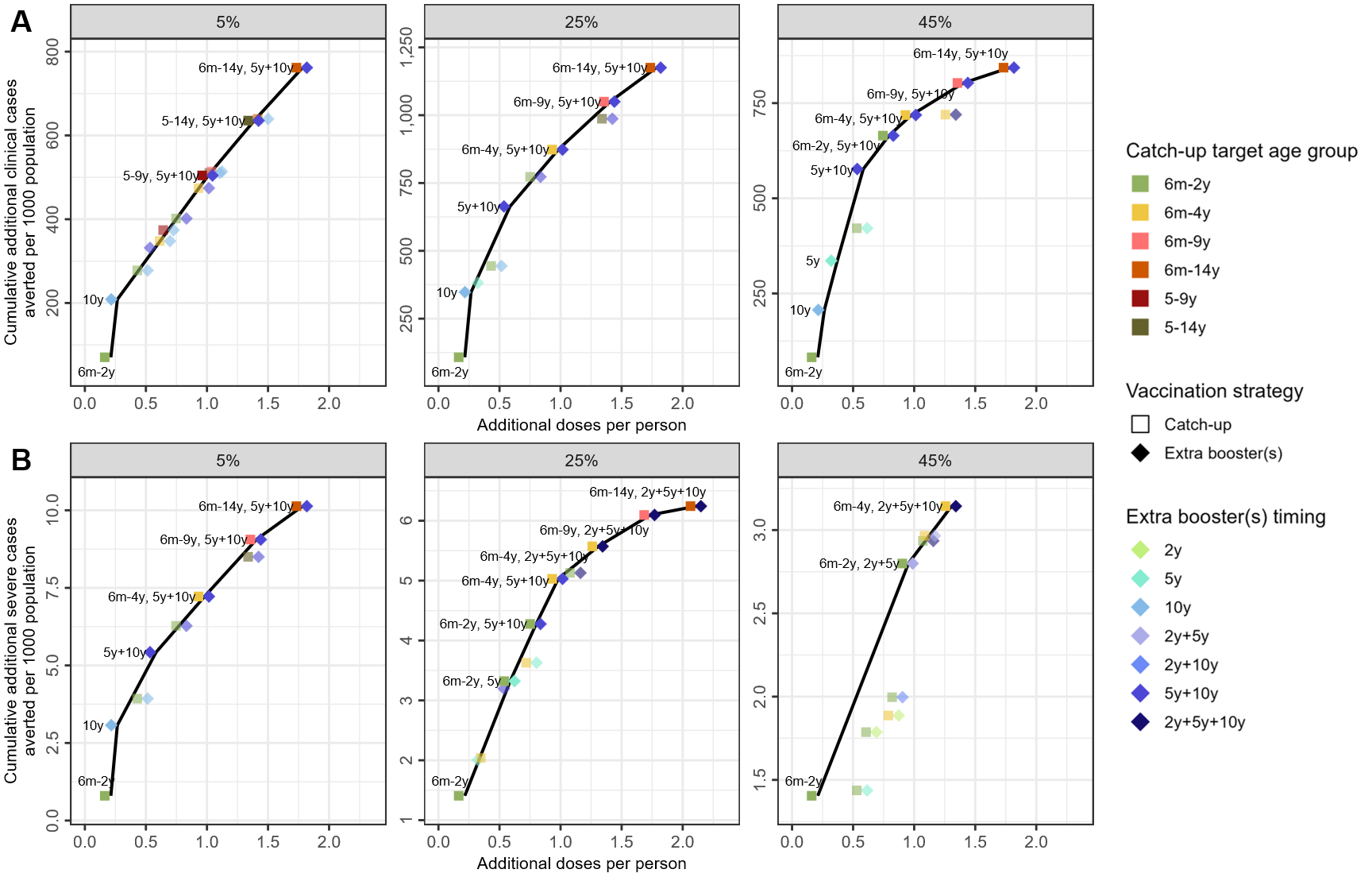
Figure S8. Efficiency frontier in seasonal settings.*** *Square points show vaccination strategies with a catch-up campaign with colours indicating the age group, and diamond-shaped points show extra booster strategies with colours indicating the booster timing. Both catch-up campaigns and extra boosters are supplementary to routine age-based vaccination. Labels indicate the number of booster doses (up to three additional boosters at 2, 5, and/or 10 years after the third dose in the routine age-based primary series) followed by the age group vaccinated in a catch-up campaign. The black line connects the points on the efficiency frontier; all strongly dominated strategies (higher cost, lower benefit) were removed. Extended dominated strategies are semi-transparent. Panel A shows efficiency frontiers for clinical cases, and Panel B shows efficiency frontiers for severe cases in perennial settings.*

***Table S4. Percent of clinical cases across the 30-year simulation in children under 5 years of age or in children 5-15 years of age****. Baseline scenarios are listed first for each transmission intensity, then scenarios by seasonality and baseline prevalence are arranged in descending order by the percent of cases in children under 5 years of age.*

| **Vaccination strategy** | **Seasonality** | **Percent of clinical cases in children under 5 years** | **Percent of clinical cases in children 5-15 years** |
| --- | --- | --- | --- |
| **Low transmission: *Pf*PR_2-10_ = 5%** | | | |
| None | seasonal | 10 (7-17) | 15 (10-24) |
| None | perennial | 10 (6-17) | 14 (10-25) |
| 6m-4y | seasonal | 6 (4-10) | 15 (10-25) |
| 6m-9y | seasonal | 6 (4-9) | 14 (10-23) |
| 6m-2y | seasonal | 6 (4-10) | 15 (10-25) |
| 6m-14y | seasonal | 6 (4-10) | 14 (9-23) |
| Routine age-based | seasonal | 6 (4-10) | 15 (10-25) |
| 5-9y | seasonal | 6 (4-10) | 14 (10-24) |
| 5-14y | seasonal | 6 (4-10) | 14 (9-23) |
| 6m-4y | perennial | 5 (4-10) | 15 (10-25) |
| 6m-9y | perennial | 5 (4-10) | 14 (10-24) |
| 6m-2y | perennial | 6 (4-10) | 15 (10-25) |
| 6m-14y | perennial | 6 (4-10) | 14 (9-24) |
| Routine age-based | perennial | 6 (4-10) | 15 (10-25) |
| 5-9y | perennial | 6 (4-10) | 14 (10-24) |
| 5-14y | perennial | 6 (4-10) | 14 (9-24) |
| **Moderate transmission: *Pf*PR_2-10_ = 25%** | | | |
| None | seasonal | 24 (17-40) | 16 (10-24) |
| None | perennial | 24 (16-39) | 16 (10-24) |
| 6m-4y | seasonal | 15 (11-25) | 20 (12-29) |
| 6m-9y | seasonal | 15 (11-25) | 19 (12-28) |
| 6m-14y | seasonal | 15 (11-25) | 19 (12-27) |
| 6m-2y | seasonal | 15 (11-25) | 19 (12-29) |
| Routine age-based | seasonal | 16 (11-26) | 19 (12-28) |
| 5-9y | seasonal | 16 (12-26) | 19 (12-28) |
| 5-14y | seasonal | 16 (11-26) | 18 (11-27) |
| 6m-4y | perennial | 14 (10-24) | 19 (12-29) |
| 6m-9y | perennial | 14 (10-24) | 19 (12-28) |
| 6m-14y | perennial | 15 (10-24) | 18 (11-27) |
| 6m-2y | perennial | 15 (10-24) | 19 (12-29) |
| Routine age-based | perennial | 15 (11-25) | 19 (12-28) |
| 5-9y | perennial | 15 (11-26) | 18 (11-27) |
| 5-14y | perennial | 15 (11-26) | 18 (11-27) |
| **High transmission: *Pf*PR_2-10_ = 45%** | | | |
| None | seasonal | 45 (29-69) | 12 (6-19) |
| None | perennial | 41 (27-66) | 13 (7-20) |
| 6m-4y | seasonal | 31 (20-49) | 18 (9-27) |
| 6m-9y | seasonal | 31 (20-49) | 17 (9-26) |
| 6m-14y | seasonal | 31 (20-49) | 17 (9-26) |
| 6m-2y | seasonal | 31 (20-49) | 17 (9-26) |
| Routine age-based | seasonal | 32 (21-51) | 17 (9-26) |
| 5-9y | seasonal | 32 (21-51) | 17 (9-25) |
| 5-14y | seasonal | 32 (21-51) | 16 (9-25) |
| 6m-4y | perennial | 28 (18-44) | 18 (10-28) |
| 6m-9y | perennial | 28 (18-44) | 18 (10-27) |
| 6m-2y | perennial | 28 (19-45) | 18 (10-27) |
| 6m-14y | perennial | 28 (19-44) | 18 (10-27) |
| Routine age-based | perennial | 29 (19-46) | 17 (9-27) |
| 5-9y | perennial | 29 (19-46) | 17 (9-26) |
| 5-14y | perennial | 29 (19-46) | 17 (9-26) |

***Table S5. Outcomes averted by combination vaccination strategies (i.e. catch-up vaccination plus extra boosters) per 1000 people, per 1000 additional doses, and per 1000 total doses over the 30-year simulation in a perennial setting****. Each of the strategies is supplementary to routine age-based vaccination that includes a single booster dose at 12 months post-primary series. The age groups listed are those targeted for a catch-up vaccination, and booster dose timing is noted as the timing and number of additional booster doses (e.g. 6m-14y; 10y booster refers to a catch-up vaccination campaign to children aged 6 months to 14 years at the beginning of the simulation plus routine age-based vaccination with a single additional booster at 10 years post primary series). Clinical and severe cases averted per 1000 population and per 1000 doses are in comparison to a baseline of no vaccination, whilst additional clinical and severe cases averted per 1000 additional doses are in comparison to a baseline of routine age-based vaccination. The table is grouped by transmission intensity. Median values with 95% credible intervals are presented.*

| ***Pf*PR_2-10_** | **Strategy** | **Clinical cases averted per 1000 population** | **Severe cases averted per 1000 population** | **Additional clinical cases averted per 1000 additional doses (relative to routine age-based)** | **Additional severe cases averted per 1000 additional doses (relative to routine age-based)** | **Clinical cases averted per 1000 doses**  **(relative to no vaccination)** | **Severe cases averted per 1000 doses (relative to no vaccination)** |
| --- | --- | --- | --- | --- | --- | --- | --- |
| **Low transmission: *Pf*PR_2-10_ = 1%** | | | | | | | |
| 1% | 6m-2y; 2y booster | 324 (261, 525) | 10 (4, 18) | 68 (12, 113) | 2 (0, 4) | 89 (72, 144) | 3 (1, 5) |
| 1% | 6m-2y; 5y booster | 395 (279, 605) | 12 (5, 20) | 173 (117, 261) | 5 (2, 8) | 110 (78, 169) | 3 (1, 6) |
| 1% | 6m-2y; 10y booster | 410 (304, 600) | 12 (5, 22) | 246 (170, 325) | 7 (3, 12) | 118 (88, 173) | 3 (2, 6) |
| 1% | 6m-2y; 5y, 10y boosters | 445 (326, 682) | 14 (6, 24) | 198 (144, 282) | 6 (2, 11) | 117 (86, 180) | 4 (1, 6) |
| 1% | 6m-2y; 2y, 5y boosters | 382 (285, 609) | 12 (5, 21) | 101 (61, 149) | 3 (1, 5) | 97 (72, 154) | 3 (1, 5) |
| 1% | 6m-2y; 2y, 10y boosters | 403 (319, 609) | 13 (5, 22) | 133 (101, 180) | 4 (2, 7) | 104 (83, 158) | 3 (1, 6) |
| 1% | 6m-2y; 2y, 5y, 10y boosters | 431 (333, 670) | 13 (6, 24) | 127 (102, 182) | 4 (2, 7) | 105 (81, 162) | 3 (1, 6) |
| 1% | 6m-4y; 2y booster | 361 (271, 580) | 11 (5, 19) | 88 (52, 141) | 2 (1, 4) | 94 (71, 151) | 3 (1, 5) |
| 1% | 6m-4y; 5y booster | 412 (329, 639) | 13 (6, 21) | 167 (129, 248) | 5 (2, 8) | 109 (87, 170) | 3 (1, 6) |
| 1% | 6m-4y; 10y booster | 432 (340, 672) | 14 (6, 24) | 218 (164, 316) | 7 (3, 11) | 118 (93, 183) | 4 (2, 6) |
| 1% | 6m-4y; 5y, 10y boosters | 478 (371, 735) | 15 (6, 26) | 194 (146, 268) | 6 (3, 10) | 120 (93, 185) | 4 (2, 6) |
| 1% | 6m-4y; 2y, 5y boosters | 413 (308, 650) | 13 (5, 22) | 111 (81, 180) | 3 (1, 6) | 100 (75, 157) | 3 (1, 5) |
| 1% | 6m-4y; 2y, 10y boosters | 444 (333, 649) | 14 (6, 23) | 142 (98, 188) | 4 (2, 7) | 110 (82, 160) | 3 (1, 6) |
| 1% | 6m-4y; 2y, 5y, 10y boosters | 460 (348, 717) | 14 (6, 24) | 132 (99, 195) | 4 (2, 7) | 107 (81, 166) | 3 (1, 6) |
| 1% | 6m-9y; 2y booster | 439 (333, 684) | 12 (5, 21) | 116 (79, 172) | 3 (1, 5) | 103 (78, 160) | 3 (1, 5) |
| 1% | 6m-9y; 5y booster | 493 (364, 761) | 15 (6, 25) | 174 (120, 252) | 5 (2, 8) | 118 (87, 182) | 3 (1, 6) |
| 1% | 6m-9y; 10y booster | 511 (389, 752) | 15 (6, 25) | 209 (153, 298) | 5 (2, 10) | 125 (95, 184) | 4 (2, 6) |
| 1% | 6m-9y; 5y, 10y boosters | 544 (421, 823) | 17 (7, 28) | 185 (140, 254) | 5 (2, 9) | 124 (96, 187) | 4 (2, 6) |
| 1% | 6m-9y; 2y, 5y boosters | 491 (380, 771) | 14 (6, 25) | 129 (94, 195) | 4 (2, 6) | 108 (83, 169) | 3 (1, 5) |
| 1% | 6m-9y; 2y, 10y boosters | 518 (395, 773) | 16 (6, 26) | 155 (114, 208) | 4 (2, 7) | 116 (88, 173) | 3 (1, 6) |
| 1% | 6m-9y; 2y, 5y, 10y boosters | 535 (415, 823) | 16 (7, 27) | 143 (104, 210) | 4 (2, 7) | 113 (88, 174) | 3 (1, 6) |
| 1% | 6m-14y; 2y booster | 501 (380, 755) | 15 (6, 25) | 131 (82, 177) | 3 (1, 6) | 108 (82, 163) | 3 (1, 5) |
| 1% | 6m-14y; 5y booster | 555 (430, 823) | 16 (7, 28) | 173 (126, 248) | 5 (2, 8) | 122 (94, 180) | 4 (1, 6) |
| 1% | 6m-14y; 10y booster | 580 (451, 848) | 17 (7, 28) | 198 (147, 278) | 5 (2, 9) | 130 (101, 190) | 4 (2, 6) |
| 1% | 6m-14y; 5y, 10y boosters | 611 (480, 897) | 18 (7, 30) | 183 (140, 248) | 5 (2, 9) | 128 (101, 187) | 4 (2, 6) |
| 1% | 6m-14y; 2y, 5y boosters | 553 (426, 831) | 16 (7, 27) | 136 (99, 194) | 4 (2, 6) | 112 (86, 168) | 3 (1, 5) |
| 1% | 6m-14y; 2y, 10y boosters | 574 (455, 840) | 17 (7, 28) | 154 (116, 217) | 4 (2, 7) | 118 (94, 173) | 3 (1, 6) |
| 1% | 6m-14y; 2y, 5y, 10y boosters | 596 (472, 880) | 18 (7, 29) | 150 (107, 208) | 4 (2, 7) | 117 (92, 172) | 4 (1, 6) |
| 1% | 5-9y; 2y booster | 384 (288, 573) | 11 (5, 19) | 111 (67, 162) | 3 (1, 5) | 100 (75, 149) | 3 (1, 5) |
| 1% | 5-9y; 5y booster | 442 (333, 644) | 13 (5, 23) | 186 (131, 291) | 5 (2, 9) | 117 (89, 171) | 3 (1, 6) |
| 1% | 5-9y; 10y booster | 452 (348, 660) | 13 (6, 23) | 236 (172, 341) | 7 (3, 12) | 123 (95, 181) | 4 (2, 6) |
| 1% | 5-9y; 5y, 10y boosters | 492 (382, 723) | 15 (6, 26) | 211 (156, 275) | 6 (2, 11) | 124 (96, 182) | 4 (2, 6) |
| 1% | 5-9y; 2y, 5y boosters | 432 (336, 669) | 13 (5, 22) | 126 (97, 179) | 3 (1, 6) | 105 (81, 162) | 3 (1, 5) |
| 1% | 5-9y; 2y, 10y boosters | 459 (351, 671) | 14 (6, 24) | 157 (122, 212) | 5 (2, 7) | 113 (87, 166) | 3 (1, 6) |
| 1% | 5-9y; 2y, 5y, 10y boosters | 483 (376, 718) | 15 (6, 25) | 147 (113, 201) | 4 (2, 8) | 112 (87, 167) | 3 (1, 6) |
| 1% | 5-14y; 2y booster | 438 (335, 684) | 13 (5, 21) | 131 (88, 192) | 3 (1, 5) | 104 (80, 162) | 3 (1, 5) |
| 1% | 5-14y; 5y booster | 503 (388, 751) | 15 (6, 25) | 180 (136, 271) | 5 (2, 8) | 122 (94, 182) | 4 (1, 6) |
| 1% | 5-14y; 10y booster | 523 (407, 753) | 15 (6, 26) | 225 (167, 290) | 6 (3, 11) | 129 (101, 187) | 4 (2, 7) |
| 1% | 5-14y; 5y, 10y boosters | 559 (432, 818) | 17 (7, 29) | 200 (151, 270) | 6 (2, 10) | 128 (99, 188) | 4 (2, 7) |
| 1% | 5-14y; 2y, 5y boosters | 491 (387, 744) | 15 (6, 24) | 138 (100, 187) | 4 (2, 6) | 109 (86, 165) | 3 (1, 5) |
| 1% | 5-14y; 2y, 10y boosters | 515 (400, 765) | 16 (6, 25) | 160 (117, 225) | 5 (2, 7) | 116 (90, 173) | 4 (1, 6) |
| 1% | 5-14y; 2y, 5y, 10y boosters | 544 (427, 784) | 17 (7, 27) | 153 (111, 205) | 4 (2, 7) | 116 (91, 168) | 4 (1, 6) |
| **Low transmission: *Pf*PR_2-10_ = 5%** | | | | | | | |
| 5% | 6m-2y; 2y booster | 828 (596, 1202) | 16 (7, 25) | 158 (94, 249) | 2 (0, 5) | 226 (163, 329) | 4 (2, 7) |
| 5% | 6m-2y; 5y booster | 976 (711, 1394) | 19 (8, 28) | 411 (298, 602) | 7 (3, 11) | 272 (199, 390) | 5 (2, 8) |
| 5% | 6m-2y; 10y booster | 999 (744, 1414) | 19 (8, 28) | 538 (387, 722) | 8 (3, 11) | 287 (214, 406) | 5 (2, 8) |
| 5% | 6m-2y; 5y, 10y boosters | 1128 (822, 1567) | 21 (9, 33) | 480 (353, 637) | 8 (4, 12) | 298 (217, 413) | 6 (2, 9) |
| 5% | 6m-2y; 2y, 5y boosters | 976 (710, 1372) | 19 (8, 29) | 253 (171, 351) | 4 (2, 7) | 246 (179, 347) | 5 (2, 7) |
| 5% | 6m-2y; 2y, 10y boosters | 1002 (742, 1407) | 19 (8, 29) | 323 (238, 441) | 5 (2, 8) | 259 (192, 363) | 5 (2, 8) |
| 5% | 6m-2y; 2y, 5y, 10y boosters | 1078 (786, 1500) | 21 (9, 32) | 306 (219, 429) | 5 (2, 8) | 262 (191, 364) | 5 (2, 8) |
| 5% | 6m-4y; 2y booster | 882 (645, 1284) | 18 (7, 26) | 193 (135, 290) | 3 (1, 5) | 230 (168, 335) | 5 (2, 7) |
| 5% | 6m-4y; 5y booster | 1030 (746, 1467) | 20 (8, 30) | 391 (279, 568) | 7 (3, 10) | 274 (199, 390) | 5 (2, 8) |
| 5% | 6m-4y; 10y booster | 1059 (777, 1484) | 20 (8, 30) | 489 (361, 683) | 7 (3, 12) | 290 (212, 405) | 6 (2, 8) |
| 5% | 6m-4y; 5y, 10y boosters | 1172 (858, 1660) | 22 (10, 33) | 445 (337, 617) | 8 (3, 11) | 295 (216, 417) | 6 (3, 8) |
| 5% | 6m-4y; 2y, 5y boosters | 1027 (748, 1471) | 20 (8, 30) | 260 (193, 387) | 4 (2, 7) | 248 (181, 356) | 5 (2, 7) |
| 5% | 6m-4y; 2y, 10y boosters | 1076 (788, 1507) | 20 (9, 31) | 320 (247, 453) | 5 (2, 8) | 266 (194, 372) | 5 (2, 8) |
| 5% | 6m-4y; 2y, 5y, 10y boosters | 1138 (844, 1597) | 22 (9, 33) | 306 (231, 419) | 5 (2, 8) | 265 (196, 371) | 5 (2, 8) |
| 5% | 6m-9y; 2y booster | 1034 (758, 1498) | 19 (8, 29) | 246 (186, 343) | 3 (1, 5) | 243 (178, 351) | 5 (2, 7) |
| 5% | 6m-9y; 5y booster | 1182 (879, 1667) | 22 (9, 32) | 383 (290, 528) | 6 (3, 8) | 282 (210, 398) | 5 (2, 8) |
| 5% | 6m-9y; 10y booster | 1218 (900, 1690) | 22 (9, 32) | 441 (326, 587) | 6 (3, 9) | 298 (220, 414) | 5 (2, 8) |
| 5% | 6m-9y; 5y, 10y boosters | 1327 (987, 1831) | 24 (10, 36) | 419 (319, 577) | 7 (3, 9) | 302 (224, 416) | 6 (2, 8) |
| 5% | 6m-9y; 2y, 5y boosters | 1184 (868, 1662) | 22 (9, 32) | 290 (208, 399) | 4 (2, 6) | 260 (190, 365) | 5 (2, 7) |
| 5% | 6m-9y; 2y, 10y boosters | 1222 (912, 1724) | 22 (9, 33) | 327 (257, 463) | 5 (2, 7) | 273 (203, 385) | 5 (2, 7) |
| 5% | 6m-9y; 2y, 5y, 10y boosters | 1300 (950, 1787) | 23 (10, 35) | 324 (237, 443) | 5 (2, 7) | 275 (201, 378) | 5 (2, 7) |
| 5% | 6m-14y; 2y booster | 1160 (866, 1612) | 20 (8, 29) | 261 (198, 354) | 3 (1, 5) | 250 (186, 348) | 4 (2, 6) |
| 5% | 6m-14y; 5y booster | 1306 (967, 1814) | 23 (9, 33) | 365 (273, 499) | 5 (2, 7) | 286 (212, 397) | 5 (2, 7) |
| 5% | 6m-14y; 10y booster | 1321 (1004, 1825) | 23 (10, 34) | 409 (310, 550) | 5 (2, 8) | 296 (225, 409) | 5 (2, 8) |
| 5% | 6m-14y; 5y, 10y boosters | 1444 (1088, 1988) | 25 (11, 38) | 397 (305, 538) | 6 (3, 9) | 302 (228, 416) | 5 (2, 8) |
| 5% | 6m-14y; 2y, 5y boosters | 1285 (966, 1800) | 23 (10, 34) | 291 (221, 395) | 4 (2, 6) | 260 (196, 365) | 5 (2, 7) |
| 5% | 6m-14y; 2y, 10y boosters | 1352 (1007, 1872) | 23 (10, 35) | 331 (250, 458) | 5 (2, 6) | 278 (207, 386) | 5 (2, 7) |
| 5% | 6m-14y; 2y, 5y, 10y boosters | 1404 (1054, 1943) | 25 (11, 37) | 316 (247, 434) | 5 (2, 7) | 275 (207, 380) | 5 (2, 7) |
| 5% | 5-9y; 2y booster | 914 (657, 1299) | 17 (7, 26) | 220 (150, 314) | 3 (1, 5) | 238 (171, 339) | 4 (2, 7) |
| 5% | 5-9y; 5y booster | 1063 (779, 1471) | 20 (8, 30) | 424 (315, 579) | 6 (3, 9) | 283 (207, 391) | 5 (2, 8) |
| 5% | 5-9y; 10y booster | 1087 (798, 1488) | 20 (8, 29) | 522 (393, 698) | 7 (3, 11) | 297 (218, 406) | 5 (2, 8) |
| 5% | 5-9y; 5y, 10y boosters | 1195 (894, 1656) | 22 (9, 33) | 468 (359, 642) | 7 (3, 11) | 301 (225, 417) | 5 (2, 8) |
| 5% | 5-9y; 2y, 5y boosters | 1049 (763, 1482) | 19 (8, 30) | 274 (211, 385) | 4 (2, 6) | 253 (185, 359) | 5 (2, 7) |
| 5% | 5-9y; 2y, 10y boosters | 1085 (799, 1525) | 20 (9, 31) | 346 (257, 471) | 5 (2, 8) | 268 (198, 376) | 5 (2, 8) |
| 5% | 5-9y; 2y, 5y, 10y boosters | 1160 (845, 1622) | 22 (9, 33) | 328 (247, 457) | 5 (2, 8) | 269 (197, 377) | 5 (2, 8) |
| 5% | 5-14y; 2y booster | 1027 (772, 1461) | 19 (8, 27) | 251 (189, 334) | 3 (1, 4) | 244 (183, 347) | 4 (2, 6) |
| 5% | 5-14y; 5y booster | 1182 (876, 1629) | 21 (9, 31) | 386 (300, 524) | 5 (2, 8) | 285 (212, 393) | 5 (2, 8) |
| 5% | 5-14y; 10y booster | 1211 (904, 1659) | 21 (9, 31) | 455 (341, 623) | 6 (3, 9) | 300 (224, 411) | 5 (2, 8) |
| 5% | 5-14y; 5y, 10y boosters | 1314 (986, 1842) | 23 (10, 35) | 426 (338, 592) | 6 (3, 9) | 302 (226, 423) | 5 (2, 8) |
| 5% | 5-14y; 2y, 5y boosters | 1164 (874, 1642) | 21 (9, 31) | 287 (221, 392) | 4 (2, 6) | 258 (194, 364) | 5 (2, 7) |
| 5% | 5-14y; 2y, 10y boosters | 1213 (904, 1692) | 21 (9, 32) | 341 (250, 446) | 5 (2, 7) | 274 (204, 382) | 5 (2, 7) |
| 5% | 5-14y; 2y, 5y, 10y boosters | 1290 (955, 1782) | 23 (10, 35) | 327 (252, 442) | 5 (2, 7) | 276 (204, 381) | 5 (2, 7) |
| **Moderate transmission: *Pf*PR_2-10_ = 25%** | | | | | | | |
| 25% | 6m-2y; 2y booster | 1857 (1383, 2535) | 27 (16, 40) | 250 (152, 411) | 3 (1, 6) | 509 (379, 695) | 7 (4, 11) |
| 25% | 6m-2y; 5y booster | 2147 (1580, 2937) | 28 (17, 42) | 760 (538, 1028) | 5 (2, 9) | 600 (442, 822) | 8 (5, 12) |
| 25% | 6m-2y; 10y booster | 2123 (1582, 2846) | 27 (16, 40) | 862 (610, 1159) | 3 (1, 7) | 611 (455, 820) | 8 (5, 12) |
| 25% | 6m-2y; 5y, 10y boosters | 2432 (1808, 3260) | 29 (17, 45) | 896 (657, 1169) | 5 (2, 9) | 641 (477, 860) | 8 (5, 12) |
| 25% | 6m-2y; 2y, 5y boosters | 2169 (1602, 2964) | 29 (17, 42) | 482 (334, 680) | 4 (2, 7) | 549 (407, 752) | 7 (4, 11) |
| 25% | 6m-2y; 2y, 10y boosters | 2169 (1631, 2923) | 28 (17, 42) | 566 (403, 771) | 4 (2, 6) | 562 (422, 757) | 7 (4, 11) |
| 25% | 6m-2y; 2y, 5y, 10y boosters | 2383 (1775, 3208) | 30 (18, 44) | 611 (443, 820) | 4 (2, 6) | 580 (431, 780) | 7 (4, 11) |
| 25% | 6m-4y; 2y booster | 1931 (1442, 2693) | 28 (16, 41) | 291 (209, 443) | 3 (1, 6) | 504 (377, 702) | 7 (4, 11) |
| 25% | 6m-4y; 5y booster | 2220 (1671, 3038) | 29 (17, 43) | 696 (500, 956) | 4 (2, 8) | 590 (444, 807) | 8 (4, 11) |
| 25% | 6m-4y; 10y booster | 2230 (1632, 2950) | 27 (17, 43) | 757 (539, 1045) | 3 (1, 8) | 610 (447, 807) | 7 (5, 12) |
| 25% | 6m-4y; 5y, 10y boosters | 2509 (1873, 3398) | 30 (17, 45) | 831 (607, 1130) | 5 (3, 8) | 632 (472, 856) | 7 (4, 11) |
| 25% | 6m-4y; 2y, 5y boosters | 2239 (1667, 3081) | 30 (17, 45) | 471 (344, 666) | 4 (2, 6) | 542 (404, 746) | 7 (4, 11) |
| 25% | 6m-4y; 2y, 10y boosters | 2293 (1698, 3081) | 29 (17, 43) | 559 (406, 750) | 4 (2, 6) | 567 (420, 761) | 7 (4, 11) |
| 25% | 6m-4y; 2y, 5y, 10y boosters | 2448 (1837, 3359) | 30 (18, 45) | 581 (428, 809) | 4 (2, 6) | 570 (428, 782) | 7 (4, 10) |
| 25% | 6m-9y; 2y booster | 2127 (1568, 2940) | 28 (17, 42) | 335 (233, 495) | 2 (1, 4) | 499 (368, 690) | 7 (4, 10) |
| 25% | 6m-9y; 5y booster | 2407 (1773, 3280) | 29 (17, 43) | 590 (418, 825) | 3 (2, 6) | 575 (424, 784) | 7 (4, 10) |
| 25% | 6m-9y; 10y booster | 2392 (1770, 3218) | 28 (16, 42) | 627 (449, 871) | 3 (1, 4) | 587 (434, 790) | 7 (4, 10) |
| 25% | 6m-9y; 5y, 10y boosters | 2705 (1983, 3605) | 31 (18, 47) | 704 (504, 942) | 4 (2, 6) | 615 (451, 820) | 7 (4, 11) |
| 25% | 6m-9y; 2y, 5y boosters | 2421 (1791, 3289) | 30 (17, 44) | 467 (330, 620) | 3 (2, 5) | 532 (393, 723) | 6 (4, 10) |
| 25% | 6m-9y; 2y, 10y boosters | 2458 (1824, 3335) | 29 (17, 44) | 516 (364, 685) | 2 (1, 4) | 549 (409, 745) | 6 (4, 10) |
| 25% | 6m-9y; 2y, 5y, 10y boosters | 2648 (1957, 3546) | 30 (17, 46) | 543 (393, 714) | 3 (2, 5) | 561 (414, 750) | 6 (4, 10) |
| 25% | 6m-14y; 2y booster | 2237 (1653, 3074) | 28 (17, 42) | 330 (230, 475) | 2 (1, 3) | 483 (357, 663) | 6 (4, 9) |
| 25% | 6m-14y; 5y booster | 2531 (1863, 3482) | 29 (17, 44) | 526 (372, 755) | 3 (1, 5) | 555 (409, 764) | 6 (4, 10) |
| 25% | 6m-14y; 10y booster | 2527 (1867, 3393) | 28 (17, 42) | 552 (394, 770) | 2 (1, 4) | 567 (419, 762) | 6 (4, 10) |
| 25% | 6m-14y; 5y, 10y boosters | 2820 (2083, 3803) | 31 (18, 44) | 621 (453, 852) | 3 (2, 5) | 591 (437, 796) | 6 (4, 9) |
| 25% | 6m-14y; 2y, 5y boosters | 2539 (1894, 3486) | 30 (18, 45) | 434 (318, 599) | 3 (2, 4) | 515 (384, 707) | 6 (4, 9) |
| 25% | 6m-14y; 2y, 10y boosters | 2602 (1900, 3514) | 29 (18, 44) | 474 (341, 674) | 2 (1, 4) | 537 (392, 725) | 6 (4, 9) |
| 25% | 6m-14y; 2y, 5y, 10y boosters | 2757 (2052, 3752) | 31 (18, 47) | 500 (367, 694) | 3 (1, 5) | 541 (403, 735) | 6 (4, 9) |
| 25% | 5-9y; 2y booster | 1939 (1425, 2636) | 26 (15, 39) | 283 (180, 409) | 1 (-1, 3) | 506 (372, 688) | 7 (4, 10) |
| 25% | 5-9y; 5y booster | 2214 (1650, 3002) | 27 (16, 41) | 679 (476, 943) | 3 (0, 6) | 588 (438, 799) | 7 (4, 11) |
| 25% | 5-9y; 10y booster | 2186 (1640, 2939) | 26 (15, 39) | 740 (538, 1008) | 1 (-1, 4) | 599 (450, 805) | 7 (4, 11) |
| 25% | 5-9y; 5y, 10y boosters | 2501 (1859, 3319) | 29 (16, 43) | 807 (592, 1047) | 3 (1, 6) | 630 (468, 836) | 7 (4, 11) |
| 25% | 5-9y; 2y, 5y boosters | 2223 (1652, 3000) | 28 (16, 42) | 459 (331, 619) | 3 (1, 4) | 538 (400, 725) | 7 (4, 10) |
| 25% | 5-9y; 2y, 10y boosters | 2261 (1681, 3057) | 27 (16, 41) | 538 (390, 742) | 2 (1, 4) | 559 (416, 755) | 7 (4, 10) |
| 25% | 5-9y; 2y, 5y, 10y boosters | 2457 (1807, 3273) | 29 (17, 45) | 568 (409, 761) | 3 (1, 5) | 572 (420, 762) | 7 (4, 10) |
| 25% | 5-14y; 2y booster | 2094 (1514, 2799) | 27 (16, 40) | 295 (205, 434) | 1 (0, 3) | 498 (360, 665) | 6 (4, 10) |
| 25% | 5-14y; 5y booster | 2353 (1758, 3208) | 27 (16, 42) | 560 (406, 780) | 2 (1, 4) | 570 (424, 775) | 7 (4, 10) |
| 25% | 5-14y; 10y booster | 2330 (1725, 3107) | 25 (15, 40) | 584 (407, 818) | 1 (0, 3) | 578 (427, 770) | 6 (4, 10) |
| 25% | 5-14y; 5y, 10y boosters | 2637 (1936, 3534) | 29 (17, 44) | 671 (487, 913) | 3 (2, 5) | 607 (446, 813) | 7 (4, 10) |
| 25% | 5-14y; 2y, 5y boosters | 2343 (1753, 3169) | 28 (17, 43) | 435 (314, 587) | 2 (1, 4) | 520 (389, 703) | 6 (4, 9) |
| 25% | 5-14y; 2y, 10y boosters | 2420 (1788, 3247) | 27 (16, 42) | 483 (349, 673) | 2 (1, 3) | 547 (404, 735) | 6 (4, 10) |
| 25% | 5-14y; 2y, 5y, 10y boosters | 2566 (1914, 3466) | 29 (18, 44) | 516 (375, 706) | 3 (1, 4) | 549 (409, 742) | 6 (4, 9) |
| **Moderately high transmission: *Pf*PR_2-10_ = 45%** | | | | | | | |
| 45% | 6m-2y; 2y booster | 2096 (1512, 2860) | 30 (16, 48) | 221 (124, 358) | 3 (0, 6) | 575 (415, 785) | 8 (4, 13) |
| 45% | 6m-2y; 5y booster | 2342 (1717, 3230) | 30 (16, 46) | 659 (488, 984) | 3 (-1, 5) | 656 (481, 904) | 8 (4, 13) |
| 45% | 6m-2y; 10y booster | 2239 (1605, 3055) | 28 (15, 44) | 609 (402, 905) | 1 (-4, 4) | 647 (463, 882) | 8 (4, 13) |
| 45% | 6m-2y; 5y, 10y boosters | 2586 (1857, 3536) | 30 (16, 47) | 772 (557, 1091) | 2 (0, 5) | 684 (491, 933) | 8 (4, 12) |
| 45% | 6m-2y; 2y, 5y boosters | 2385 (1695, 3260) | 31 (16, 48) | 443 (295, 656) | 3 (1, 6) | 605 (430, 826) | 8 (4, 12) |
| 45% | 6m-2y; 2y, 10y boosters | 2384 (1684, 3206) | 30 (16, 47) | 467 (305, 666) | 2 (0, 4) | 619 (437, 832) | 8 (4, 12) |
| 45% | 6m-2y; 2y, 5y, 10y boosters | 2586 (1867, 3554) | 31 (16, 49) | 543 (404, 746) | 2 (1, 5) | 629 (454, 862) | 8 (4, 12) |
| 45% | 6m-4y; 2y booster | 2147 (1540, 2919) | 30 (16, 47) | 232 (142, 362) | 2 (-1, 5) | 561 (401, 761) | 8 (4, 12) |
| 45% | 6m-4y; 5y booster | 2406 (1720, 3319) | 31 (16, 47) | 571 (410, 835) | 3 (-1, 6) | 640 (458, 884) | 8 (4, 13) |
| 45% | 6m-4y; 10y booster | 2293 (1636, 3175) | 28 (15, 46) | 498 (320, 818) | 1 (-3, 4) | 628 (448, 868) | 8 (4, 12) |
| 45% | 6m-4y; 5y, 10y boosters | 2650 (1890, 3682) | 31 (16, 48) | 679 (491, 1017) | 2 (-1, 4) | 666 (477, 929) | 8 (4, 12) |
| 45% | 6m-4y; 2y, 5y boosters | 2456 (1755, 3293) | 32 (17, 49) | 413 (308, 593) | 2 (0, 5) | 595 (425, 798) | 8 (4, 12) |
| 45% | 6m-4y; 2y, 10y boosters | 2401 (1703, 3337) | 30 (16, 48) | 411 (280, 645) | 2 (0, 4) | 594 (421, 825) | 8 (4, 12) |
| 45% | 6m-4y; 2y, 5y, 10y boosters | 2644 (1912, 3604) | 31 (17, 51) | 520 (369, 735) | 2 (0, 5) | 615 (444, 839) | 7 (4, 12) |
| 45% | 6m-9y; 2y booster | 2219 (1593, 3090) | 29 (16, 47) | 203 (120, 350) | 1 (-1, 3) | 521 (374, 727) | 7 (4, 11) |
| 45% | 6m-9y; 5y booster | 2480 (1791, 3398) | 30 (16, 47) | 431 (296, 640) | 1 (-1, 3) | 593 (428, 813) | 7 (4, 11) |
| 45% | 6m-9y; 10y booster | 2360 (1687, 3279) | 28 (15, 46) | 369 (255, 588) | 0 (-2, 2) | 579 (413, 805) | 7 (4, 11) |
| 45% | 6m-9y; 5y, 10y boosters | 2703 (1938, 3754) | 30 (16, 48) | 523 (370, 797) | 1 (-1, 3) | 614 (441, 856) | 7 (4, 11) |
| 45% | 6m-9y; 2y, 5y boosters | 2531 (1803, 3488) | 31 (17, 49) | 353 (245, 536) | 2 (0, 3) | 556 (397, 766) | 7 (4, 11) |
| 45% | 6m-9y; 2y, 10y boosters | 2471 (1778, 3423) | 30 (16, 48) | 354 (241, 537) | 1 (-1, 3) | 553 (398, 765) | 7 (4, 11) |
| 45% | 6m-9y; 2y, 5y, 10y boosters | 2727 (1930, 3788) | 31 (17, 49) | 419 (297, 644) | 2 (0, 3) | 577 (408, 802) | 7 (3, 10) |
| 45% | 6m-14y; 2y booster | 2279 (1634, 3138) | 29 (15, 48) | 192 (117, 286) | 1 (-1, 2) | 492 (353, 677) | 6 (3, 10) |
| 45% | 6m-14y; 5y booster | 2527 (1807, 3467) | 29 (15, 46) | 357 (254, 530) | 1 (-1, 2) | 555 (396, 761) | 6 (3, 10) |
| 45% | 6m-14y; 10y booster | 2412 (1721, 3347) | 28 (15, 45) | 303 (210, 477) | 0 (-2, 1) | 541 (386, 751) | 6 (3, 10) |
| 45% | 6m-14y; 5y, 10y boosters | 2775 (1969, 3838) | 30 (16, 46) | 439 (316, 652) | 1 (-1, 2) | 581 (413, 804) | 6 (3, 10) |
| 45% | 6m-14y; 2y, 5y boosters | 2585 (1840, 3518) | 31 (16, 49) | 312 (226, 445) | 1 (0, 3) | 524 (373, 714) | 6 (3, 10) |
| 45% | 6m-14y; 2y, 10y boosters | 2520 (1821, 3494) | 30 (16, 48) | 302 (211, 446) | 1 (-1, 2) | 520 (376, 722) | 6 (3, 10) |
| 45% | 6m-14y; 2y, 5y, 10y boosters | 2758 (1980, 3792) | 30 (17, 48) | 369 (263, 548) | 1 (0, 2) | 540 (388, 743) | 6 (3, 9) |
| 45% | 5-9y; 2y booster | 2086 (1519, 2846) | 29 (15, 45) | 169 (90, 285) | 0 (-3, 3) | 545 (396, 744) | 8 (4, 12) |
| 45% | 5-9y; 5y booster | 2363 (1711, 3270) | 29 (15, 45) | 514 (354, 793) | 0 (-3, 2) | 629 (457, 871) | 8 (4, 12) |
| 45% | 5-9y; 10y booster | 2242 (1596, 3091) | 27 (15, 43) | 422 (258, 676) | -2 (-5, 1) | 614 (437, 848) | 7 (4, 12) |
| 45% | 5-9y; 5y, 10y boosters | 2602 (1873, 3597) | 28 (16, 45) | 637 (427, 941) | 0 (-3, 2) | 656 (472, 905) | 7 (4, 11) |
| 45% | 5-9y; 2y, 5y boosters | 2361 (1734, 3286) | 30 (17, 46) | 372 (271, 551) | 1 (0, 3) | 573 (420, 795) | 7 (4, 11) |
| 45% | 5-9y; 2y, 10y boosters | 2354 (1696, 3290) | 29 (15, 47) | 380 (258, 588) | 1 (-1, 2) | 581 (419, 815) | 7 (4, 12) |
| 45% | 5-9y; 2y, 5y, 10y boosters | 2586 (1861, 3534) | 30 (16, 47) | 454 (335, 662) | 1 (-1, 2) | 603 (433, 824) | 7 (4, 11) |
| 45% | 5-14y; 2y booster | 2147 (1560, 2913) | 28 (15, 45) | 156 (97, 258) | 0 (-2, 2) | 510 (371, 692) | 7 (4, 11) |
| 45% | 5-14y; 5y booster | 2398 (1723, 3316) | 28 (16, 45) | 384 (254, 559) | 0 (-2, 1) | 580 (417, 802) | 7 (4, 11) |
| 45% | 5-14y; 10y booster | 2265 (1644, 3131) | 27 (14, 43) | 304 (181, 493) | -1 (-4, 1) | 562 (408, 778) | 7 (3, 11) |
| 45% | 5-14y; 5y, 10y boosters | 2643 (1891, 3650) | 28 (16, 45) | 484 (354, 730) | 0 (-2, 1) | 607 (435, 840) | 6 (4, 10) |
| 45% | 5-14y; 2y, 5y boosters | 2432 (1757, 3341) | 29 (16, 47) | 316 (206, 466) | 1 (-1, 2) | 540 (390, 743) | 6 (3, 10) |
| 45% | 5-14y; 2y, 10y boosters | 2423 (1746, 3319) | 28 (16, 45) | 305 (202, 487) | 0 (-1, 1) | 547 (395, 751) | 6 (4, 10) |
| 45% | 5-14y; 2y, 5y, 10y boosters | 2612 (1887, 3634) | 29 (16, 47) | 389 (276, 568) | 1 (0, 2) | 559 (403, 778) | 6 (3, 10) |
| **High transmission: *Pf*PR_2-10_ = 65%** | | | | | | | |
| 65% | 6m-2y; 2y booster | 1852 (1367, 2774) | 28 (10, 63) | 144 (68, 334) | 2 (-1, 5) | 508 (375, 761) | 8 (3, 17) |
| 65% | 6m-2y; 5y booster | 1982 (1455, 2992) | 28 (10, 64) | 400 (248, 658) | 1 (-3, 4) | 555 (407, 837) | 8 (3, 18) |
| 65% | 6m-2y; 10y booster | 1874 (1346, 2807) | 27 (9, 63) | 217 (104, 446) | -1 (-5, 2) | 540 (388, 809) | 8 (3, 18) |
| 65% | 6m-2y; 5y, 10y boosters | 2104 (1545, 3198) | 27 (8, 64) | 417 (264, 724) | 0 (-3, 3) | 555 (408, 844) | 7 (2, 17) |
| 65% | 6m-2y; 2y, 5y boosters | 2060 (1484, 3058) | 29 (10, 64) | 298 (192, 511) | 1 (-1, 4) | 523 (376, 774) | 7 (2, 16) |
| 65% | 6m-2y; 2y, 10y boosters | 1997 (1443, 2991) | 28 (10, 63) | 267 (154, 485) | 1 (-1, 3) | 518 (373, 775) | 7 (3, 16) |
| 65% | 6m-2y; 2y, 5y, 10y boosters | 2149 (1570, 3228) | 29 (9, 65) | 333 (221, 528) | 1 (-1, 3) | 522 (382, 784) | 7 (2, 16) |
| 65% | 6m-4y; 2y booster | 1882 (1373, 2830) | 29 (8, 65) | 140 (51, 294) | 1 (-2, 5) | 492 (359, 740) | 8 (2, 17) |
| 65% | 6m-4y; 5y booster | 2021 (1481, 3041) | 28 (8, 63) | 322 (195, 591) | 0 (-2, 3) | 540 (395, 809) | 7 (2, 17) |
| 65% | 6m-4y; 10y booster | 1888 (1376, 2870) | 26 (8, 63) | 183 (82, 429) | -1 (-4, 3) | 518 (378, 786) | 7 (2, 17) |
| 65% | 6m-4y; 5y, 10y boosters | 2116 (1568, 3209) | 27 (9, 65) | 366 (230, 657) | 0 (-2, 3) | 533 (396, 809) | 7 (2, 16) |
| 65% | 6m-4y; 2y, 5y boosters | 2065 (1526, 3142) | 29 (9, 66) | 279 (173, 452) | 1 (-1, 4) | 500 (370, 763) | 7 (2, 16) |
| 65% | 6m-4y; 2y, 10y boosters | 2012 (1449, 3046) | 28 (8, 65) | 231 (144, 434) | 1 (-2, 3) | 499 (358, 754) | 7 (2, 16) |
| 65% | 6m-4y; 2y, 5y, 10y boosters | 2171 (1579, 3292) | 28 (9, 65) | 314 (198, 516) | 1 (-1, 4) | 506 (368, 767) | 7 (2, 15) |
| 65% | 6m-9y; 2y booster | 1883 (1377, 2826) | 28 (8, 64) | 100 (44, 198) | 0 (-2, 2) | 443 (323, 665) | 7 (2, 15) |
| 65% | 6m-9y; 5y booster | 2008 (1455, 3034) | 28 (7, 63) | 219 (122, 378) | 0 (-2, 2) | 482 (348, 726) | 7 (2, 15) |
| 65% | 6m-9y; 10y booster | 1892 (1378, 2897) | 26 (7, 61) | 135 (43, 292) | -1 (-4, 0) | 465 (338, 711) | 6 (2, 15) |
| 65% | 6m-9y; 5y, 10y boosters | 2122 (1525, 3240) | 26 (7, 63) | 275 (160, 481) | -1 (-2, 1) | 484 (347, 738) | 6 (2, 14) |
| 65% | 6m-9y; 2y, 5y boosters | 2066 (1510, 3128) | 28 (8, 64) | 205 (129, 352) | 0 (-1, 3) | 455 (332, 688) | 6 (2, 14) |
| 65% | 6m-9y; 2y, 10y boosters | 2009 (1455, 3042) | 28 (9, 64) | 168 (104, 303) | 0 (-2, 1) | 450 (326, 681) | 6 (2, 14) |
| 65% | 6m-9y; 2y, 5y, 10y boosters | 2180 (1583, 3259) | 29 (8, 65) | 251 (161, 380) | 0 (-1, 2) | 463 (336, 690) | 6 (2, 14) |
| 65% | 6m-14y; 2y booster | 1872 (1338, 2859) | 28 (7, 64) | 74 (25, 178) | 0 (-2, 2) | 404 (288, 617) | 6 (1, 14) |
| 65% | 6m-14y; 5y booster | 1988 (1436, 3032) | 26 (8, 62) | 164 (80, 309) | -1 (-2, 1) | 436 (315, 666) | 6 (2, 14) |
| 65% | 6m-14y; 10y booster | 1884 (1327, 2871) | 25 (8, 62) | 90 (25, 218) | -2 (-3, 1) | 423 (298, 645) | 6 (2, 14) |
| 65% | 6m-14y; 5y, 10y boosters | 2134 (1516, 3271) | 26 (7, 63) | 212 (116, 393) | -1 (-2, 1) | 448 (318, 687) | 6 (2, 13) |
| 65% | 6m-14y; 2y, 5y boosters | 2062 (1494, 3137) | 28 (8, 63) | 161 (97, 300) | 0 (-1, 1) | 419 (303, 638) | 6 (2, 13) |
| 65% | 6m-14y; 2y, 10y boosters | 1997 (1416, 3046) | 27 (7, 63) | 133 (66, 267) | 0 (-2, 1) | 412 (292, 629) | 6 (1, 13) |
| 65% | 6m-14y; 2y, 5y, 10y boosters | 2159 (1574, 3337) | 28 (7, 63) | 196 (123, 366) | 0 (-2, 1) | 424 (308, 655) | 6 (1, 12) |
| 65% | 5-9y; 2y booster | 1830 (1346, 2729) | 27 (8, 60) | 77 (15, 157) | 0 (-2, 1) | 479 (352, 712) | 7 (2, 16) |
| 65% | 5-9y; 5y booster | 1985 (1425, 2965) | 27 (8, 62) | 255 (122, 458) | -1 (-4, 1) | 529 (380, 790) | 7 (2, 16) |
| 65% | 5-9y; 10y booster | 1842 (1331, 2783) | 25 (7, 61) | 123 (9, 299) | -3 (-6, 0) | 504 (364, 764) | 7 (2, 17) |
| 65% | 5-9y; 5y, 10y boosters | 2090 (1510, 3111) | 26 (8, 61) | 324 (180, 540) | -2 (-4, 1) | 526 (381, 784) | 7 (2, 15) |
| 65% | 5-9y; 2y, 5y boosters | 2003 (1472, 3029) | 28 (8, 61) | 224 (141, 403) | 0 (-2, 2) | 485 (357, 735) | 7 (2, 15) |
| 65% | 5-9y; 2y, 10y boosters | 1953 (1432, 2933) | 27 (8, 61) | 184 (106, 359) | -1 (-3, 1) | 483 (355, 726) | 7 (2, 15) |
| 65% | 5-9y; 2y, 5y, 10y boosters | 2124 (1569, 3219) | 28 (8, 61) | 275 (164, 458) | 0 (-2, 2) | 495 (365, 751) | 6 (2, 14) |
| 65% | 5-14y; 2y booster | 1817 (1328, 2750) | 26 (8, 60) | 45 (1, 118) | -1 (-3, 1) | 431 (316, 654) | 6 (2, 14) |
| 65% | 5-14y; 5y booster | 1950 (1421, 2919) | 25 (8, 62) | 174 (86, 312) | -2 (-3, 0) | 472 (344, 705) | 6 (2, 15) |
| 65% | 5-14y; 10y booster | 1837 (1315, 2792) | 24 (7, 60) | 76 (-38, 220) | -3 (-5, 0) | 457 (326, 694) | 6 (2, 15) |
| 65% | 5-14y; 5y, 10y boosters | 2054 (1518, 3139) | 25 (7, 62) | 250 (135, 408) | -2 (-4, 0) | 472 (349, 721) | 6 (2, 14) |
| 65% | 5-14y; 2y, 5y boosters | 2009 (1477, 3042) | 28 (7, 61) | 172 (89, 294) | 0 (-2, 0) | 446 (328, 675) | 6 (2, 14) |
| 65% | 5-14y; 2y, 10y boosters | 1952 (1406, 2970) | 26 (7, 61) | 138 (71, 254) | -1 (-3, 1) | 442 (318, 672) | 6 (2, 14) |
| 65% | 5-14y; 2y, 5y, 10y boosters | 2104 (1567, 3188) | 27 (8, 63) | 215 (122, 378) | -1 (-2, 1) | 450 (336, 683) | 6 (2, 14) |

***Table S6. Percent of clinical and severe cases averted in whole population and in children under 5 years of age by scenario over a 30-year simulation****. Only combined strategies (catch-up plus additional boosters) and routine age-based vaccination are shown. Table is grouped by transmission intensity and sorted in descending order by percent of cases averted in the whole population within each transmission and seasonality setting.*

| **Vaccination strategy** | ***Pf*PR_2-10_** | **Seasonality** | **Percent of cases averted in whole population** | **Percent of severe cases averted in whole population** | **Percent of cases averted in U5s** | **Percent of severe cases averted in U5s** |
| --- | --- | --- | --- | --- | --- | --- |
| **Perennial low transmission: *Pf*PR_2-10_ = 5%** | | | | | | |
| 6m-14y; 5y, 10y boosters | 5% | perennial | 34 (30, 38) | 30 (26, 33) | 60 (57, 63) | 56 (52, 59) |
| 6m-14y; 2y, 5y, 10y boosters | 5% | perennial | 33 (30, 37) | 29 (26, 32) | 61 (59, 64) | 57 (53, 60) |
| 6m-14y; 2y, 10y boosters | 5% | perennial | 32 (28, 35) | 28 (24, 31) | 60 (58, 63) | 56 (53, 60) |
| 6m-9y; 5y, 10y boosters | 5% | perennial | 32 (28, 35) | 28 (25, 32) | 59 (56, 61) | 55 (50, 57) |
| 6m-14y; 10y booster | 5% | perennial | 31 (28, 35) | 27 (23, 30) | 59 (56, 61) | 55 (51, 58) |
| 6m-14y; 5y booster | 5% | perennial | 31 (27, 35) | 27 (24, 29) | 59 (56, 61) | 54 (51, 57) |
| 6m-9y; 2y, 5y, 10y boosters | 5% | perennial | 31 (27, 34) | 28 (24, 31) | 60 (58, 63) | 56 (52, 60) |
| 5-14y; 5y, 10y boosters | 5% | perennial | 31 (28, 34) | 28 (24, 31) | 55 (53, 58) | 52 (48, 54) |
| 6m-14y; 2y, 5y boosters | 5% | perennial | 31 (27, 35) | 27 (23, 30) | 60 (58, 63) | 56 (52, 60) |
| 5-14y; 2y, 5y, 10y boosters | 5% | perennial | 30 (27, 34) | 27 (24, 30) | 57 (54, 59) | 53 (49, 55) |
| 6m-9y; 2y, 10y boosters | 5% | perennial | 29 (26, 32) | 26 (23, 30) | 59 (57, 62) | 55 (51, 58) |
| 6m-9y; 10y booster | 5% | perennial | 29 (25, 32) | 25 (23, 29) | 58 (55, 60) | 53 (50, 56) |
| 5-14y; 2y, 10y boosters | 5% | perennial | 28 (26, 32) | 26 (22, 28) | 56 (53, 58) | 52 (49, 55) |
| 5-14y; 10y booster | 5% | perennial | 28 (26, 31) | 25 (22, 28) | 54 (52, 56) | 50 (47, 53) |
| 5-9y; 5y, 10y boosters | 5% | perennial | 28 (25, 32) | 26 (24, 29) | 54 (51, 56) | 50 (46, 52) |
| 6m-9y; 5y booster | 5% | perennial | 28 (25, 31) | 25 (23, 28) | 57 (55, 60) | 53 (49, 56) |
| 5-14y; 5y booster | 5% | perennial | 28 (25, 31) | 25 (22, 27) | 54 (52, 57) | 50 (46, 53) |
| 6m-9y; 2y, 5y boosters | 5% | perennial | 28 (25, 31) | 25 (23, 28) | 59 (57, 62) | 55 (51, 59) |
| 6m-4y; 5y, 10y boosters | 5% | perennial | 28 (24, 31) | 27 (23, 29) | 57 (55, 59) | 53 (50, 56) |
| 5-14y; 2y, 5y boosters | 5% | perennial | 28 (25, 31) | 25 (22, 28) | 56 (53, 58) | 52 (48, 55) |
| 6m-14y; 2y booster | 5% | perennial | 27 (24, 30) | 24 (20, 27) | 59 (57, 61) | 55 (52, 58) |
| 5-9y; 2y, 5y, 10y boosters | 5% | perennial | 27 (24, 31) | 26 (23, 29) | 55 (53, 57) | 51 (48, 54) |
| 6m-4y; 2y, 5y, 10y boosters | 5% | perennial | 27 (24, 31) | 26 (23, 28) | 59 (56, 61) | 55 (52, 58) |
| 6m-2y; 5y, 10y boosters | 5% | perennial | 26 (23, 30) | 25 (23, 28) | 56 (53, 59) | 52 (49, 55) |
| 5-9y; 2y, 10y boosters | 5% | perennial | 26 (23, 29) | 24 (21, 27) | 55 (52, 57) | 51 (48, 53) |
| 5-9y; 10y booster | 5% | perennial | 25 (22, 29) | 23 (21, 26) | 53 (50, 55) | 49 (46, 52) |
| 6m-2y; 2y, 5y, 10y boosters | 5% | perennial | 25 (22, 29) | 25 (22, 28) | 57 (55, 60) | 53 (50, 56) |
| 6m-4y; 2y, 10y boosters | 5% | perennial | 25 (22, 29) | 24 (21, 27) | 58 (56, 60) | 54 (51, 57) |
| 5-9y; 5y booster | 5% | perennial | 25 (22, 28) | 23 (21, 26) | 52 (50, 55) | 49 (45, 51) |
| 6m-4y; 10y booster | 5% | perennial | 25 (22, 28) | 24 (21, 26) | 56 (53, 58) | 52 (48, 54) |
| 5-9y; 2y, 5y boosters | 5% | perennial | 25 (21, 29) | 23 (20, 26) | 54 (52, 57) | 50 (47, 53) |
| 5-14y; 2y booster | 5% | perennial | 25 (22, 27) | 22 (19, 24) | 54 (52, 56) | 50 (48, 53) |
| 6m-9y; 2y booster | 5% | perennial | 25 (22, 28) | 23 (20, 25) | 58 (56, 60) | 54 (51, 57) |
| 6m-4y; 5y booster | 5% | perennial | 24 (21, 28) | 23 (20, 27) | 56 (53, 58) | 52 (48, 55) |
| 6m-4y; 2y, 5y boosters | 5% | perennial | 24 (21, 29) | 23 (21, 26) | 58 (55, 60) | 54 (50, 57) |
| 6m-2y; 2y, 10y boosters | 5% | perennial | 24 (21, 27) | 23 (20, 26) | 56 (54, 58) | 52 (49, 54) |
| 6m-2y; 10y booster | 5% | perennial | 23 (21, 26) | 22 (20, 25) | 54 (52, 56) | 51 (47, 53) |
| 6m-2y; 5y booster | 5% | perennial | 23 (20, 26) | 22 (20, 26) | 54 (52, 57) | 51 (47, 54) |
| 6m-2y; 2y, 5y boosters | 5% | perennial | 23 (20, 26) | 23 (20, 26) | 56 (54, 58) | 52 (49, 55) |
| 5-9y; 2y booster | 5% | perennial | 21 (19, 25) | 20 (18, 23) | 53 (51, 55) | 49 (46, 52) |
| 6m-4y; 2y booster | 5% | perennial | 21 (18, 24) | 20 (17, 23) | 56 (54, 58) | 52 (49, 55) |
| 6m-2y; 2y booster | 5% | perennial | 19 (17, 23) | 19 (17, 23) | 55 (53, 57) | 51 (48, 54) |
| Routine age-based | 5% | perennial | 17 (15, 20) | 17 (15, 19) | 50 (48, 52) | 47 (43, 48) |
| **Seasonal low transmission: *Pf*PR_2-10_ = 5%** | | | | | | |
| 6m-14y; 5y, 10y boosters | 5% | seasonal | 34 (31, 37) | 30 (27, 33) | 60 (57, 62) | 56 (52, 60) |
| 6m-14y; 2y, 5y, 10y boosters | 5% | seasonal | 34 (30, 37) | 29 (26, 33) | 61 (59, 63) | 57 (53, 61) |
| 6m-14y; 2y, 10y boosters | 5% | seasonal | 32 (28, 35) | 28 (24, 31) | 61 (58, 63) | 57 (53, 59) |
| 6m-14y; 10y booster | 5% | seasonal | 32 (28, 34) | 27 (24, 31) | 59 (57, 61) | 55 (51, 58) |
| 5-14y; 5y, 10y boosters | 5% | seasonal | 32 (28, 34) | 28 (24, 31) | 56 (53, 57) | 52 (47, 54) |
| 6m-9y; 5y, 10y boosters | 5% | seasonal | 32 (28, 35) | 29 (25, 32) | 59 (56, 61) | 55 (51, 59) |
| 6m-14y; 5y booster | 5% | seasonal | 31 (27, 34) | 27 (24, 31) | 59 (56, 61) | 55 (51, 58) |
| 6m-14y; 2y, 5y boosters | 5% | seasonal | 31 (27, 34) | 27 (23, 32) | 60 (58, 63) | 56 (52, 60) |
| 6m-9y; 2y, 5y, 10y boosters | 5% | seasonal | 31 (27, 34) | 28 (24, 33) | 60 (58, 63) | 56 (52, 59) |
| 5-14y; 2y, 5y, 10y boosters | 5% | seasonal | 31 (27, 34) | 27 (24, 30) | 57 (54, 59) | 53 (49, 56) |
| 6m-9y; 2y, 10y boosters | 5% | seasonal | 29 (26, 32) | 26 (23, 29) | 59 (57, 61) | 55 (52, 59) |
| 5-14y; 2y, 10y boosters | 5% | seasonal | 29 (26, 32) | 25 (22, 28) | 56 (54, 58) | 52 (48, 55) |
| 6m-9y; 10y booster | 5% | seasonal | 29 (25, 32) | 26 (23, 29) | 57 (55, 60) | 53 (50, 57) |
| 5-14y; 10y booster | 5% | seasonal | 29 (26, 31) | 25 (22, 29) | 54 (52, 56) | 51 (46, 54) |
| 5-9y; 5y, 10y boosters | 5% | seasonal | 28 (25, 32) | 26 (23, 29) | 54 (51, 57) | 50 (46, 54) |
| 6m-9y; 5y booster | 5% | seasonal | 28 (25, 31) | 25 (23, 29) | 57 (55, 60) | 53 (50, 57) |
| 5-14y; 5y booster | 5% | seasonal | 28 (25, 31) | 25 (22, 28) | 54 (52, 56) | 50 (46, 53) |
| 6m-9y; 2y, 5y boosters | 5% | seasonal | 28 (24, 31) | 25 (22, 29) | 59 (57, 61) | 55 (51, 59) |
| 6m-4y; 5y, 10y boosters | 5% | seasonal | 28 (24, 31) | 26 (23, 30) | 57 (55, 59) | 53 (49, 56) |
| 5-14y; 2y, 5y boosters | 5% | seasonal | 28 (25, 31) | 25 (22, 28) | 56 (54, 58) | 52 (48, 55) |
| 6m-14y; 2y booster | 5% | seasonal | 28 (24, 31) | 24 (21, 28) | 59 (57, 61) | 55 (51, 58) |
| 5-9y; 2y, 5y, 10y boosters | 5% | seasonal | 28 (24, 31) | 26 (22, 28) | 55 (53, 58) | 51 (48, 56) |
| 6m-4y; 2y, 5y, 10y boosters | 5% | seasonal | 27 (23, 30) | 26 (22, 30) | 58 (56, 61) | 55 (51, 58) |
| 6m-2y; 5y, 10y boosters | 5% | seasonal | 26 (23, 29) | 25 (22, 29) | 56 (53, 58) | 52 (48, 54) |
| 5-9y; 2y, 10y boosters | 5% | seasonal | 26 (23, 29) | 24 (21, 28) | 54 (52, 57) | 51 (47, 54) |
| 5-9y; 10y booster | 5% | seasonal | 26 (23, 28) | 23 (21, 26) | 52 (50, 55) | 49 (45, 51) |
| 6m-2y; 2y, 5y, 10y boosters | 5% | seasonal | 26 (22, 29) | 25 (21, 28) | 57 (55, 59) | 53 (50, 56) |
| 5-9y; 5y booster | 5% | seasonal | 25 (22, 28) | 23 (20, 27) | 52 (50, 55) | 49 (45, 52) |
| 6m-4y; 2y, 10y boosters | 5% | seasonal | 25 (22, 28) | 24 (21, 28) | 58 (56, 60) | 54 (50, 58) |
| 6m-4y; 10y booster | 5% | seasonal | 25 (22, 28) | 23 (20, 27) | 56 (53, 58) | 52 (48, 55) |
| 5-9y; 2y, 5y boosters | 5% | seasonal | 25 (22, 28) | 23 (20, 26) | 54 (52, 57) | 50 (47, 54) |
| 6m-9y; 2y booster | 5% | seasonal | 25 (22, 28) | 22 (20, 26) | 58 (56, 60) | 54 (50, 57) |
| 6m-4y; 5y booster | 5% | seasonal | 25 (21, 27) | 23 (20, 26) | 56 (54, 58) | 52 (47, 55) |
| 5-14y; 2y booster | 5% | seasonal | 25 (22, 28) | 22 (19, 25) | 54 (53, 57) | 51 (47, 54) |
| 6m-4y; 2y, 5y boosters | 5% | seasonal | 24 (21, 27) | 23 (20, 27) | 57 (55, 60) | 53 (50, 57) |
| 6m-2y; 2y, 10y boosters | 5% | seasonal | 24 (21, 27) | 23 (20, 27) | 56 (54, 58) | 52 (49, 55) |
| 6m-2y; 10y booster | 5% | seasonal | 23 (21, 26) | 22 (20, 27) | 54 (52, 56) | 50 (47, 54) |
| 6m-2y; 5y booster | 5% | seasonal | 23 (20, 26) | 23 (19, 26) | 54 (52, 56) | 50 (46, 53) |
| 6m-2y; 2y, 5y boosters | 5% | seasonal | 23 (20, 26) | 22 (19, 26) | 56 (54, 58) | 52 (48, 55) |
| 5-9y; 2y booster | 5% | seasonal | 22 (19, 25) | 20 (18, 23) | 53 (51, 55) | 49 (46, 52) |
| 6m-4y; 2y booster | 5% | seasonal | 21 (18, 25) | 21 (17, 25) | 56 (54, 59) | 52 (49, 56) |
| 6m-2y; 2y booster | 5% | seasonal | 19 (17, 22) | 19 (17, 23) | 54 (53, 56) | 50 (47, 54) |
| Routine age-based | 5% | seasonal | 17 (15, 20) | 18 (15, 22) | 50 (48, 52) | 46 (44, 50) |
| **Perennial moderate transmission: *Pf*PR_2-10_ = 25%** | | | | | | |
| 6m-14y; 5y, 10y boosters | 25% | perennial | 20 (19, 22) | 20 (15, 27) | 49 (47, 51) | 37 (32, 42) |
| 6m-14y; 2y, 5y, 10y boosters | 25% | perennial | 20 (18, 22) | 20 (15, 27) | 51 (48, 52) | 38 (34, 43) |
| 6m-9y; 5y, 10y boosters | 25% | perennial | 20 (18, 21) | 20 (15, 26) | 48 (46, 50) | 37 (32, 41) |
| 6m-9y; 2y, 5y, 10y boosters | 25% | perennial | 19 (17, 21) | 20 (15, 27) | 50 (48, 52) | 38 (34, 43) |
| 5-14y; 5y, 10y boosters | 25% | perennial | 19 (17, 21) | 19 (14, 25) | 45 (42, 46) | 34 (30, 38) |
| 6m-14y; 2y, 10y boosters | 25% | perennial | 19 (17, 20) | 19 (15, 26) | 50 (48, 52) | 38 (34, 44) |
| 5-14y; 2y, 5y, 10y boosters | 25% | perennial | 19 (17, 20) | 19 (14, 25) | 47 (45, 48) | 36 (32, 41) |
| 6m-14y; 2y, 5y boosters | 25% | perennial | 18 (17, 20) | 20 (15, 26) | 50 (48, 52) | 38 (34, 44) |
| 6m-14y; 5y booster | 25% | perennial | 18 (16, 20) | 19 (14, 26) | 48 (46, 50) | 36 (32, 41) |
| 6m-4y; 5y, 10y boosters | 25% | perennial | 18 (17, 20) | 20 (15, 26) | 48 (46, 50) | 36 (32, 41) |
| 6m-14y; 10y booster | 25% | perennial | 18 (17, 20) | 18 (13, 25) | 48 (46, 50) | 36 (32, 42) |
| 5-9y; 5y, 10y boosters | 25% | perennial | 18 (16, 20) | 19 (14, 24) | 44 (42, 46) | 34 (30, 39) |
| 6m-4y; 2y, 5y, 10y boosters | 25% | perennial | 18 (16, 20) | 20 (15, 26) | 50 (48, 52) | 38 (35, 43) |
| 6m-9y; 2y, 10y boosters | 25% | perennial | 18 (16, 19) | 20 (14, 25) | 50 (48, 51) | 38 (33, 43) |
| 5-9y; 2y, 5y, 10y boosters | 25% | perennial | 18 (16, 19) | 19 (14, 25) | 46 (44, 48) | 35 (32, 41) |
| 6m-2y; 5y, 10y boosters | 25% | perennial | 18 (16, 19) | 19 (15, 25) | 47 (45, 49) | 35 (32, 41) |
| 6m-9y; 5y booster | 25% | perennial | 18 (16, 19) | 19 (14, 25) | 48 (46, 50) | 36 (32, 41) |
| 6m-9y; 2y, 5y boosters | 25% | perennial | 18 (16, 19) | 20 (15, 26) | 50 (48, 52) | 38 (34, 43) |
| 6m-2y; 2y, 5y, 10y boosters | 25% | perennial | 17 (16, 19) | 20 (15, 25) | 49 (47, 51) | 37 (33, 42) |
| 6m-9y; 10y booster | 25% | perennial | 17 (16, 19) | 18 (13, 24) | 48 (45, 49) | 36 (32, 41) |
| 5-14y; 2y, 10y boosters | 25% | perennial | 17 (16, 19) | 18 (14, 24) | 46 (44, 48) | 36 (32, 40) |
| 5-14y; 2y, 5y boosters | 25% | perennial | 17 (15, 19) | 19 (14, 25) | 46 (44, 48) | 36 (32, 40) |
| 5-14y; 5y booster | 25% | perennial | 17 (16, 19) | 18 (13, 24) | 44 (42, 46) | 33 (30, 38) |
| 5-14y; 10y booster | 25% | perennial | 17 (15, 18) | 17 (12, 23) | 44 (42, 46) | 33 (30, 38) |
| 6m-4y; 2y, 10y boosters | 25% | perennial | 17 (15, 18) | 19 (14, 25) | 49 (47, 51) | 38 (34, 43) |
| 5-9y; 2y, 10y boosters | 25% | perennial | 16 (15, 18) | 18 (14, 24) | 46 (44, 47) | 35 (31, 40) |
| 6m-4y; 2y, 5y boosters | 25% | perennial | 16 (15, 18) | 20 (15, 25) | 49 (48, 51) | 38 (34, 43) |
| 6m-4y; 5y booster | 25% | perennial | 16 (15, 18) | 19 (14, 25) | 47 (45, 49) | 36 (32, 40) |
| 6m-14y; 2y booster | 25% | perennial | 16 (15, 18) | 19 (13, 25) | 50 (48, 51) | 38 (33, 43) |
| 5-9y; 2y, 5y boosters | 25% | perennial | 16 (15, 18) | 19 (14, 24) | 46 (44, 48) | 35 (32, 41) |
| 5-9y; 5y booster | 25% | perennial | 16 (15, 18) | 18 (13, 23) | 44 (42, 45) | 33 (30, 39) |
| 6m-4y; 10y booster | 25% | perennial | 16 (15, 18) | 18 (13, 24) | 47 (45, 49) | 36 (32, 41) |
| 6m-2y; 2y, 10y boosters | 25% | perennial | 16 (14, 17) | 19 (14, 24) | 48 (46, 50) | 37 (33, 42) |
| 5-9y; 10y booster | 25% | perennial | 16 (14, 17) | 17 (13, 23) | 44 (42, 45) | 33 (30, 38) |
| 6m-2y; 2y, 5y boosters | 25% | perennial | 16 (14, 17) | 19 (14, 25) | 48 (47, 50) | 37 (33, 42) |
| 6m-2y; 5y booster | 25% | perennial | 16 (14, 17) | 18 (14, 25) | 46 (44, 48) | 35 (32, 40) |
| 6m-2y; 10y booster | 25% | perennial | 15 (14, 17) | 17 (13, 23) | 46 (44, 48) | 35 (31, 40) |
| 6m-9y; 2y booster | 25% | perennial | 15 (14, 17) | 19 (14, 24) | 49 (47, 51) | 37 (34, 43) |
| 5-14y; 2y booster | 25% | perennial | 15 (13, 16) | 18 (13, 24) | 46 (44, 48) | 35 (31, 39) |
| 6m-4y; 2y booster | 25% | perennial | 14 (13, 16) | 18 (13, 24) | 49 (47, 51) | 37 (34, 43) |
| 5-9y; 2y booster | 25% | perennial | 14 (13, 15) | 17 (12, 23) | 45 (43, 47) | 35 (31, 39) |
| 6m-2y; 2y booster | 25% | perennial | 14 (12, 15) | 18 (13, 23) | 48 (46, 50) | 37 (33, 41) |
| Routine age-based | 25% | perennial | 12 (11, 14) | 16 (12, 22) | 44 (42, 46) | 34 (31, 38) |
| **Seasonal moderate transmission: *Pf*PR_2-10_ = 25%** | | | | | | |
| 6m-14y; 5y, 10y boosters | 25% | seasonal | 21 (19, 22) | 21 (15, 27) | 48 (46, 50) | 36 (31, 42) |
| 6m-14y; 2y, 5y, 10y boosters | 25% | seasonal | 20 (18, 21) | 21 (15, 27) | 50 (48, 52) | 38 (34, 43) |
| 6m-9y; 5y, 10y boosters | 25% | seasonal | 20 (18, 21) | 21 (15, 26) | 48 (45, 50) | 36 (31, 41) |
| 6m-9y; 2y, 5y, 10y boosters | 25% | seasonal | 19 (17, 21) | 21 (15, 27) | 50 (47, 51) | 38 (33, 43) |
| 5-14y; 5y, 10y boosters | 25% | seasonal | 19 (17, 20) | 20 (14, 25) | 44 (42, 46) | 34 (29, 38) |
| 5-14y; 2y, 5y, 10y boosters | 25% | seasonal | 19 (17, 20) | 20 (15, 25) | 46 (44, 48) | 35 (31, 40) |
| 6m-14y; 2y, 10y boosters | 25% | seasonal | 19 (17, 20) | 20 (14, 26) | 50 (48, 51) | 38 (33, 43) |
| 6m-14y; 2y, 5y boosters | 25% | seasonal | 19 (17, 20) | 21 (15, 27) | 50 (48, 51) | 38 (33, 44) |
| 6m-14y; 5y booster | 25% | seasonal | 18 (17, 20) | 20 (14, 26) | 48 (45, 49) | 36 (31, 41) |
| 6m-4y; 5y, 10y boosters | 25% | seasonal | 18 (17, 20) | 20 (15, 26) | 47 (45, 49) | 36 (31, 41) |
| 5-9y; 5y, 10y boosters | 25% | seasonal | 18 (17, 20) | 20 (14, 25) | 44 (42, 46) | 33 (29, 38) |
| 6m-14y; 10y booster | 25% | seasonal | 18 (17, 20) | 19 (13, 25) | 47 (45, 49) | 35 (31, 41) |
| 6m-9y; 2y, 10y boosters | 25% | seasonal | 18 (16, 19) | 20 (14, 26) | 49 (47, 51) | 38 (33, 43) |
| 5-9y; 2y, 5y, 10y boosters | 25% | seasonal | 18 (16, 19) | 20 (14, 25) | 46 (44, 48) | 35 (31, 40) |
| 6m-4y; 2y, 5y, 10y boosters | 25% | seasonal | 18 (16, 19) | 21 (15, 26) | 49 (47, 51) | 37 (33, 43) |
| 6m-9y; 2y, 5y boosters | 25% | seasonal | 18 (16, 19) | 20 (15, 26) | 49 (47, 51) | 38 (33, 43) |
| 6m-9y; 5y booster | 25% | seasonal | 18 (16, 19) | 20 (14, 26) | 47 (45, 49) | 36 (31, 41) |
| 6m-2y; 5y, 10y boosters | 25% | seasonal | 18 (16, 19) | 20 (14, 25) | 46 (44, 48) | 35 (31, 40) |
| 5-14y; 2y, 10y boosters | 25% | seasonal | 18 (16, 19) | 19 (13, 24) | 46 (44, 47) | 35 (32, 40) |
| 6m-9y; 10y booster | 25% | seasonal | 17 (16, 19) | 19 (13, 25) | 47 (45, 49) | 36 (31, 40) |
| 6m-2y; 2y, 5y, 10y boosters | 25% | seasonal | 17 (16, 19) | 20 (15, 26) | 48 (46, 50) | 37 (33, 42) |
| 5-14y; 2y, 5y boosters | 25% | seasonal | 17 (16, 18) | 19 (14, 25) | 46 (44, 48) | 35 (31, 40) |
| 5-14y; 5y booster | 25% | seasonal | 17 (16, 18) | 19 (13, 24) | 44 (42, 46) | 33 (29, 38) |
| 5-14y; 10y booster | 25% | seasonal | 17 (15, 18) | 18 (12, 23) | 44 (42, 45) | 33 (29, 38) |
| 5-9y; 2y, 10y boosters | 25% | seasonal | 17 (15, 18) | 19 (14, 25) | 45 (44, 47) | 35 (31, 40) |
| 6m-4y; 2y, 10y boosters | 25% | seasonal | 17 (15, 18) | 20 (14, 25) | 49 (47, 50) | 37 (33, 42) |
| 6m-4y; 2y, 5y boosters | 25% | seasonal | 16 (15, 18) | 20 (14, 25) | 49 (47, 50) | 37 (33, 43) |
| 6m-4y; 5y booster | 25% | seasonal | 16 (15, 18) | 19 (14, 25) | 47 (45, 49) | 36 (31, 40) |
| 6m-14y; 2y booster | 25% | seasonal | 16 (15, 18) | 19 (13, 25) | 49 (47, 51) | 37 (33, 42) |
| 5-9y; 2y, 5y boosters | 25% | seasonal | 16 (15, 18) | 19 (14, 25) | 45 (43, 47) | 35 (30, 40) |
| 5-9y; 5y booster | 25% | seasonal | 16 (15, 18) | 19 (13, 24) | 43 (41, 45) | 33 (29, 38) |
| 5-9y; 10y booster | 25% | seasonal | 16 (15, 17) | 18 (12, 23) | 43 (41, 45) | 33 (29, 38) |
| 6m-4y; 10y booster | 25% | seasonal | 16 (15, 17) | 18 (13, 24) | 47 (45, 48) | 35 (31, 40) |
| 6m-2y; 2y, 10y boosters | 25% | seasonal | 16 (14, 17) | 19 (13, 25) | 48 (46, 49) | 37 (32, 42) |
| 6m-2y; 2y, 5y boosters | 25% | seasonal | 16 (14, 17) | 20 (14, 26) | 48 (46, 49) | 37 (32, 41) |
| 6m-2y; 5y booster | 25% | seasonal | 16 (14, 17) | 19 (14, 24) | 46 (44, 47) | 35 (30, 40) |
| 6m-9y; 2y booster | 25% | seasonal | 15 (14, 17) | 19 (14, 25) | 49 (47, 50) | 37 (33, 42) |
| 6m-2y; 10y booster | 25% | seasonal | 15 (14, 16) | 18 (12, 24) | 46 (44, 47) | 35 (30, 40) |
| 5-14y; 2y booster | 25% | seasonal | 15 (14, 16) | 18 (13, 24) | 45 (43, 47) | 35 (31, 39) |
| 5-9y; 2y booster | 25% | seasonal | 14 (13, 15) | 18 (12, 23) | 45 (43, 47) | 35 (30, 40) |
| 6m-4y; 2y booster | 25% | seasonal | 14 (13, 15) | 19 (13, 24) | 48 (46, 50) | 37 (32, 42) |
| 6m-2y; 2y booster | 25% | seasonal | 14 (12, 15) | 18 (12, 24) | 47 (46, 49) | 37 (32, 41) |
| Routine age-based | 25% | seasonal | 12 (11, 13) | 17 (12, 22) | 44 (42, 45) | 33 (30, 37) |
| **Perennial moderately high transmission: *Pf*PR_2-10_ = 45%** | | | | | | |
| 6m-14y; 5y, 10y boosters | 45% | perennial | 16 (14, 16) | 18 (9, 25) | 40 (37, 42) | 27 (21, 33) |
| 6m-14y; 2y, 5y, 10y boosters | 45% | perennial | 16 (14, 16) | 18 (10, 26) | 42 (39, 44) | 28 (22, 35) |
| 6m-9y; 5y, 10y boosters | 45% | perennial | 15 (14, 16) | 18 (10, 25) | 40 (37, 42) | 27 (22, 33) |
| 6m-9y; 2y, 5y, 10y boosters | 45% | perennial | 15 (14, 16) | 18 (11, 26) | 42 (39, 44) | 29 (23, 35) |
| 6m-4y; 5y, 10y boosters | 45% | perennial | 15 (13, 16) | 18 (10, 25) | 40 (37, 42) | 27 (21, 33) |
| 6m-4y; 2y, 5y, 10y boosters | 45% | perennial | 15 (13, 16) | 18 (11, 26) | 42 (40, 44) | 29 (23, 35) |
| 5-14y; 2y, 5y, 10y boosters | 45% | perennial | 15 (13, 16) | 18 (10, 25) | 39 (36, 41) | 27 (22, 33) |
| 5-14y; 5y, 10y boosters | 45% | perennial | 15 (13, 16) | 17 (10, 24) | 37 (34, 39) | 25 (19, 32) |
| 6m-2y; 5y, 10y boosters | 45% | perennial | 15 (13, 15) | 18 (10, 25) | 39 (37, 41) | 27 (21, 33) |
| 5-9y; 5y, 10y boosters | 45% | perennial | 14 (13, 15) | 17 (10, 24) | 37 (34, 38) | 25 (20, 32) |
| 5-9y; 2y, 5y, 10y boosters | 45% | perennial | 14 (13, 15) | 18 (10, 25) | 39 (37, 41) | 27 (21, 33) |
| 6m-2y; 2y, 5y, 10y boosters | 45% | perennial | 14 (13, 15) | 18 (11, 26) | 41 (39, 43) | 29 (23, 35) |
| 6m-14y; 2y, 5y boosters | 45% | perennial | 14 (13, 15) | 18 (10, 26) | 42 (40, 44) | 28 (23, 35) |
| 6m-14y; 5y booster | 45% | perennial | 14 (13, 15) | 17 (9, 25) | 40 (37, 42) | 27 (21, 33) |
| 6m-14y; 2y, 10y boosters | 45% | perennial | 14 (13, 15) | 18 (10, 25) | 42 (39, 44) | 28 (23, 35) |
| 6m-9y; 2y, 5y boosters | 45% | perennial | 14 (13, 15) | 18 (11, 26) | 42 (39, 44) | 29 (23, 35) |
| 6m-9y; 2y, 10y boosters | 45% | perennial | 14 (13, 15) | 18 (10, 25) | 42 (39, 44) | 28 (23, 35) |
| 6m-9y; 5y booster | 45% | perennial | 14 (12, 15) | 17 (10, 24) | 40 (37, 42) | 27 (21, 33) |
| 5-14y; 2y, 5y boosters | 45% | perennial | 14 (12, 14) | 17 (10, 24) | 39 (37, 41) | 27 (21, 33) |
| 6m-4y; 2y, 5y boosters | 45% | perennial | 14 (12, 14) | 18 (11, 26) | 42 (40, 44) | 29 (23, 35) |
| 6m-4y; 2y, 10y boosters | 45% | perennial | 14 (12, 14) | 18 (11, 25) | 42 (39, 43) | 29 (24, 35) |
| 6m-4y; 5y booster | 45% | perennial | 14 (12, 14) | 18 (10, 25) | 40 (37, 41) | 27 (22, 33) |
| 6m-14y; 10y booster | 45% | perennial | 13 (12, 14) | 17 (9, 24) | 39 (37, 41) | 26 (21, 33) |
| 5-14y; 5y booster | 45% | perennial | 13 (12, 14) | 17 (9, 23) | 37 (34, 39) | 26 (20, 31) |
| 5-14y; 2y, 10y boosters | 45% | perennial | 13 (12, 14) | 17 (10, 24) | 39 (36, 41) | 27 (21, 33) |
| 5-9y; 2y, 5y boosters | 45% | perennial | 13 (12, 14) | 18 (11, 24) | 39 (37, 41) | 27 (22, 33) |
| 6m-2y; 2y, 5y boosters | 45% | perennial | 13 (12, 14) | 18 (11, 25) | 41 (39, 43) | 29 (23, 35) |
| 6m-2y; 2y, 10y boosters | 45% | perennial | 13 (12, 14) | 18 (11, 25) | 41 (39, 43) | 29 (23, 35) |
| 6m-9y; 10y booster | 45% | perennial | 13 (12, 14) | 17 (10, 23) | 39 (37, 42) | 27 (21, 33) |
| 5-9y; 5y booster | 45% | perennial | 13 (12, 14) | 17 (10, 24) | 37 (34, 38) | 25 (20, 31) |
| 5-9y; 2y, 10y boosters | 45% | perennial | 13 (12, 14) | 17 (10, 24) | 39 (36, 41) | 27 (22, 33) |
| 6m-2y; 5y booster | 45% | perennial | 13 (12, 14) | 18 (10, 25) | 39 (37, 41) | 27 (22, 33) |
| 6m-4y; 10y booster | 45% | perennial | 13 (11, 14) | 17 (10, 24) | 39 (37, 41) | 27 (22, 33) |
| 5-14y; 10y booster | 45% | perennial | 13 (11, 14) | 16 (9, 23) | 36 (34, 38) | 25 (19, 31) |
| 6m-14y; 2y booster | 45% | perennial | 13 (11, 13) | 17 (10, 24) | 42 (39, 44) | 28 (23, 35) |
| 6m-2y; 10y booster | 45% | perennial | 13 (11, 13) | 17 (10, 24) | 39 (37, 41) | 27 (22, 33) |
| 5-9y; 10y booster | 45% | perennial | 12 (11, 13) | 16 (10, 23) | 36 (34, 38) | 25 (20, 31) |
| 6m-9y; 2y booster | 45% | perennial | 12 (11, 13) | 17 (10, 25) | 42 (39, 44) | 29 (23, 35) |
| 6m-4y; 2y booster | 45% | perennial | 12 (11, 13) | 18 (10, 25) | 42 (39, 44) | 29 (23, 35) |
| 5-14y; 2y booster | 45% | perennial | 12 (11, 13) | 17 (10, 24) | 39 (37, 40) | 27 (22, 33) |
| 6m-2y; 2y booster | 45% | perennial | 12 (10, 12) | 18 (11, 25) | 41 (39, 43) | 29 (24, 35) |
| 5-9y; 2y booster | 45% | perennial | 12 (11, 12) | 17 (10, 23) | 39 (36, 40) | 27 (22, 33) |
| Routine age-based | 45% | perennial | 11 (10, 12) | 17 (10, 23) | 38 (36, 39) | 27 (22, 32) |
| **Seasonal moderately high transmission: *Pf*PR_2-10_ = 45%** | | | | | | |
| 6m-14y; 2y, 5y, 10y boosters | 45% | seasonal | 15 (14, 17) | 18 (10, 27) | 41 (38, 43) | 27 (20, 34) |
| 6m-14y; 5y, 10y boosters | 45% | seasonal | 15 (14, 17) | 18 (9, 26) | 39 (37, 40) | 26 (18, 32) |
| 6m-9y; 5y, 10y boosters | 45% | seasonal | 15 (14, 17) | 18 (10, 26) | 39 (36, 40) | 26 (19, 32) |
| 6m-9y; 2y, 5y, 10y boosters | 45% | seasonal | 15 (14, 17) | 19 (10, 28) | 41 (39, 43) | 27 (21, 34) |
| 6m-4y; 2y, 5y, 10y boosters | 45% | seasonal | 15 (13, 16) | 19 (10, 27) | 41 (39, 42) | 27 (20, 34) |
| 6m-4y; 5y, 10y boosters | 45% | seasonal | 15 (13, 16) | 18 (10, 27) | 39 (36, 40) | 26 (19, 33) |
| 5-14y; 2y, 5y, 10y boosters | 45% | seasonal | 15 (13, 16) | 18 (9, 26) | 38 (36, 40) | 26 (19, 32) |
| 5-14y; 5y, 10y boosters | 45% | seasonal | 15 (13, 16) | 17 (9, 25) | 36 (34, 37) | 24 (17, 31) |
| 6m-2y; 5y, 10y boosters | 45% | seasonal | 15 (13, 16) | 18 (10, 26) | 38 (36, 40) | 26 (19, 31) |
| 6m-2y; 2y, 5y, 10y boosters | 45% | seasonal | 15 (13, 16) | 19 (10, 27) | 40 (38, 42) | 28 (21, 33) |
| 5-9y; 2y, 5y, 10y boosters | 45% | seasonal | 14 (13, 15) | 18 (10, 26) | 38 (36, 39) | 26 (19, 33) |
| 5-9y; 5y, 10y boosters | 45% | seasonal | 14 (13, 16) | 17 (9, 25) | 36 (34, 37) | 24 (17, 30) |
| 6m-14y; 2y, 5y boosters | 45% | seasonal | 14 (13, 16) | 18 (9, 27) | 41 (39, 43) | 27 (20, 34) |
| 6m-9y; 2y, 5y boosters | 45% | seasonal | 14 (13, 15) | 19 (9, 27) | 41 (39, 42) | 27 (20, 35) |
| 6m-14y; 2y, 10y boosters | 45% | seasonal | 14 (13, 16) | 18 (10, 27) | 41 (39, 43) | 27 (20, 34) |
| 6m-14y; 5y booster | 45% | seasonal | 14 (13, 16) | 18 (9, 26) | 39 (36, 40) | 25 (19, 32) |
| 6m-9y; 5y booster | 45% | seasonal | 14 (13, 15) | 18 (9, 26) | 39 (36, 40) | 26 (19, 32) |
| 6m-9y; 2y, 10y boosters | 45% | seasonal | 14 (13, 15) | 19 (9, 27) | 41 (38, 42) | 27 (20, 34) |
| 6m-4y; 2y, 5y boosters | 45% | seasonal | 14 (12, 15) | 19 (10, 27) | 41 (39, 42) | 28 (21, 34) |
| 5-14y; 2y, 5y boosters | 45% | seasonal | 14 (12, 15) | 18 (9, 25) | 38 (36, 39) | 25 (19, 32) |
| 6m-4y; 2y, 10y boosters | 45% | seasonal | 14 (12, 15) | 18 (9, 27) | 41 (39, 42) | 27 (21, 34) |
| 6m-4y; 5y booster | 45% | seasonal | 14 (12, 15) | 18 (10, 27) | 39 (36, 40) | 26 (19, 32) |
| 6m-2y; 2y, 5y boosters | 45% | seasonal | 14 (12, 15) | 19 (10, 27) | 40 (38, 42) | 27 (21, 34) |
| 5-14y; 2y, 10y boosters | 45% | seasonal | 13 (12, 15) | 17 (9, 26) | 38 (36, 40) | 26 (19, 33) |
| 6m-14y; 10y booster | 45% | seasonal | 13 (12, 15) | 17 (8, 26) | 38 (36, 40) | 25 (18, 32) |
| 5-9y; 2y, 5y boosters | 45% | seasonal | 13 (12, 15) | 18 (10, 26) | 38 (36, 40) | 26 (19, 32) |
| 5-14y; 5y booster | 45% | seasonal | 13 (12, 15) | 17 (9, 25) | 36 (34, 37) | 24 (17, 31) |
| 6m-2y; 2y, 10y boosters | 45% | seasonal | 13 (12, 14) | 18 (10, 26) | 40 (38, 41) | 27 (21, 34) |
| 5-9y; 2y, 10y boosters | 45% | seasonal | 13 (12, 14) | 18 (9, 25) | 38 (36, 39) | 26 (19, 32) |
| 5-9y; 5y booster | 45% | seasonal | 13 (12, 14) | 17 (9, 25) | 36 (34, 37) | 24 (18, 30) |
| 6m-2y; 5y booster | 45% | seasonal | 13 (12, 14) | 18 (10, 26) | 38 (36, 40) | 26 (19, 32) |
| 6m-9y; 10y booster | 45% | seasonal | 13 (12, 15) | 17 (9, 26) | 38 (36, 40) | 26 (19, 33) |
| 6m-4y; 10y booster | 45% | seasonal | 13 (12, 14) | 17 (9, 26) | 38 (36, 40) | 26 (19, 32) |
| 6m-14y; 2y booster | 45% | seasonal | 13 (11, 14) | 18 (9, 26) | 41 (38, 42) | 27 (20, 33) |
| 5-14y; 10y booster | 45% | seasonal | 13 (12, 14) | 16 (8, 24) | 36 (33, 37) | 24 (17, 31) |
| 6m-2y; 10y booster | 45% | seasonal | 13 (11, 13) | 17 (9, 25) | 38 (36, 39) | 26 (19, 32) |
| 5-9y; 10y booster | 45% | seasonal | 13 (11, 14) | 17 (8, 24) | 36 (34, 37) | 24 (17, 31) |
| 6m-9y; 2y booster | 45% | seasonal | 13 (11, 14) | 18 (9, 26) | 40 (38, 42) | 27 (21, 34) |
| 6m-4y; 2y booster | 45% | seasonal | 12 (11, 13) | 18 (10, 26) | 41 (38, 42) | 27 (21, 34) |
| 5-14y; 2y booster | 45% | seasonal | 12 (11, 13) | 17 (9, 25) | 38 (36, 39) | 26 (19, 32) |
| 6m-2y; 2y booster | 45% | seasonal | 12 (10, 13) | 18 (10, 26) | 40 (38, 41) | 27 (21, 34) |
| 5-9y; 2y booster | 45% | seasonal | 12 (11, 13) | 17 (9, 25) | 38 (36, 39) | 26 (20, 32) |
| Routine age-based | 45% | seasonal | 11 (10, 12) | 17 (9, 25) | 37 (35, 38) | 26 (19, 32) |

# Sensitivity analysis of immunogenicity assumption

In the main text, we assume that the antibody titres and corresponding efficacy of R21/Matrix-M is consistent across all age groups because of a lack of direct evidence of differences by age. However, the immune response to vaccination may vary depending on age and prior exposure ^36^. While an age-dependent immune response is likely, its extent, particularly in older children, remains uncertain. Ongoing mass vaccination trials in The Gambia and Burkina Faso ^37^ and in Bangladesh ^38^ may provide some evidence for age-dependency of antibody immunogenicity, but these data are not yet available.

With the RTS,S/AS02 vaccine, which is similar to the WHO-recommended RTS,S/AS01 vaccine but uses a different adjuvant, the geometric mean ratio (GMR) of anti-CSP antibody titres after 3 doses was 0.64 in children aged 6-11 years at vaccination compared to those aged 1-5 years at vaccination ^39^. The R21/Matrix-M vaccine Phase III trial similarly showed a GMR of anti-CSP antibody titres after 3 doses of 0.77 (95% CI 0.69-0.87) among children aged 18-36 months compared to children aged 5-17 months, with a corresponding, but small, reduction in vaccine efficacy ^7^.

Given the limited evidence of the age-dependent immune response in school-aged children, we conducted a sensitivity analysis with two alternate immunogenicity assumptions. We tested the assumptions that the antibody response over time $CSP(t)$ to any vaccine dose, whether booster or primary series, delivered to children over age 5 years was scaled by a GMR scaling factor *s* relative to the fitted antibody titre curve from Schmit et al. ^22^,

$$CSP\left( t \right)=s*CSP_{peak}(\rho_{peak}e_{s}^{-r_{s}\left( t-t_{boost} \right)}+(1-\rho_{peak}(e_{l}^{-r_{l}\left( t-t_{boost} \right)})$$

where *s*=0.64, consistent with Bojang et al. ^39^, or *s*=0.4, a value considerably lower than either published GMR.

In the model, immunogenicity parameter values fitted to data on children aged 5-17 months were assumed for vaccination of any child under 5 years of age (see Table S2). We assume that the antibody titre dynamics over time and the relationship of antibody titre to vaccine efficacy are the same regardless of immunogenicity scaling.

The vaccine efficacy model did not incorporate antibody avidity, but this may also be an important factor in vaccine efficacy, particularly as evidence suggests that avidity, rather than antibody titre alone, may better explain protection conferred by the RTS,S/AS01 malaria vaccine, another pre-erythrocytic vaccine, and could also vary by age ^40^.

The scaled and two non-scaled estimates of antibody titres and corresponding vaccine efficacy over time are presented in Figure S9. The main analyses assume that the immunogenicity and efficacy of R21/Matrix-M are the same regardless of the age of the child at vaccination. Here, we present the sensitivity analysis results assuming that the anti-CSP antibody titres are 36% and 60% lower in children aged 5 years and older than those achieved by routine vaccination. This translates to average efficacy over one year of 71.2% with the fitted values, 65.5% with antibody titres scaled to 0.64, a reduction of 5.7 percentage points, and 58.3% with antibody titres scaled to 0.4, a reduction of 12.9 percentage points (Figure 9).

***
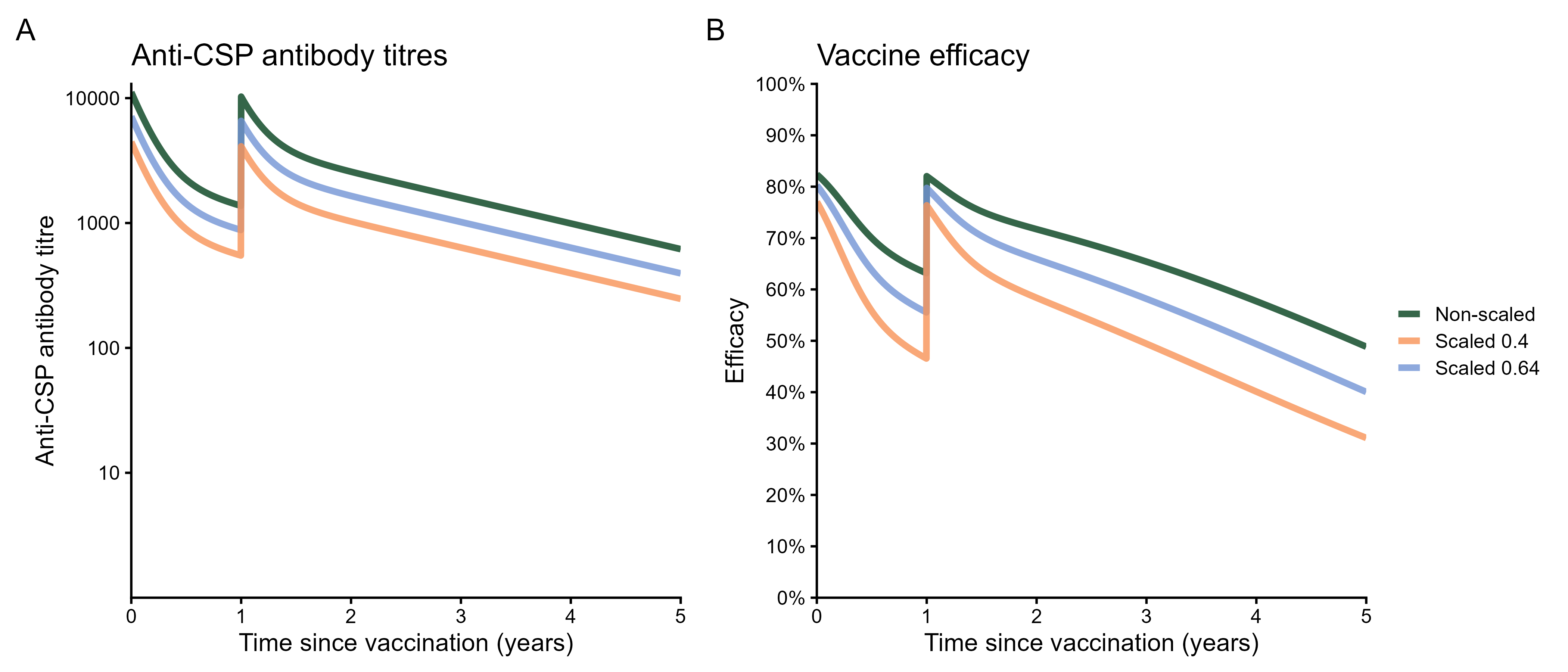
Figure S9. Comparison of scaled and non-scaled antibody titre and clinical efficacy.*** *The dark green lines refer to the antibody titres and corresponding efficacy using the fitted parameter values for the R21/Matrix-M vaccine from Schmit et al.* (11)*, while the light blue line shows antibody titres scaled to 0.64 and corresponding efficacy, and the orange line shows antibody titres scaled to 0.4 and corresponding efficacy.*

As expected, when the vaccine-induced antibody titres in older children are scaled, the impact of catch-up campaigns and extra booster doses is reduced; however, the impact is limited, and the main conclusions are largely consistent with the main analysis where we have assumed no age scaling of antibody titres (Figures S10-S14). For example, a catch-up vaccination scenario in a 5% *Pf*PR_2-10_ perennial setting targeting children aged 6 months to 14 years averted 1141 (95% CrI 843-1587) clinical cases per 1000 people over 30 years in the main analysis, 1075 (95% CrI 800-1516) clinical cases per 1000 people when assuming scaled immunogenicity of 0.64 among children 5 years or older, and 1044 (95% CrI 770-1462) clinical cases per 1000 people when assuming scaled immunogenicity of 0.4 in children 5 years or older (Tables 2, S8, S9).

Considering scenarios with extra booster doses, lower immunogenicity and thus efficacy for the booster dose at combinations of 5 and/or 10 years after the primary series had the biggest reduction of cases averted compared to no scaling (Figure S14). Over a 15-year simulation in a perennial 5% *Pf*PR_2-10_ setting, a strategy with extra booster doses at 5 and 10 years post primary series averted 1041 (95% CrI 756-1452) clinical cases per 1000 people when assuming no difference in efficacy in older children, 956 (95% CrI 704-1365) clinical cases per 1000 people when assuming scaled immunogenicity to 0.64 in older children, and 878 (95% CrI 649-1263) clinical cases per 1000 people when assuming scaled immunogenicity of 0.4 in older children (Tables 2, S8, S9).

Similar patterns are seen with clinical and severe cases averted per 1000 additional doses. Table 2, Table S8, and Table S9 show clinical and severe cases averted per 1000 people and per 1000 additional doses under the different assumptions. Although there are slight differences, the confidence intervals overlap in most comparisons between assumptions. The limited impact of reduced immunogenicity in high transmission settings is likely because most cases in these settings are among the youngest children, so a change in vaccine efficacy among older children would not meaningfully influence overall cases averted.

***
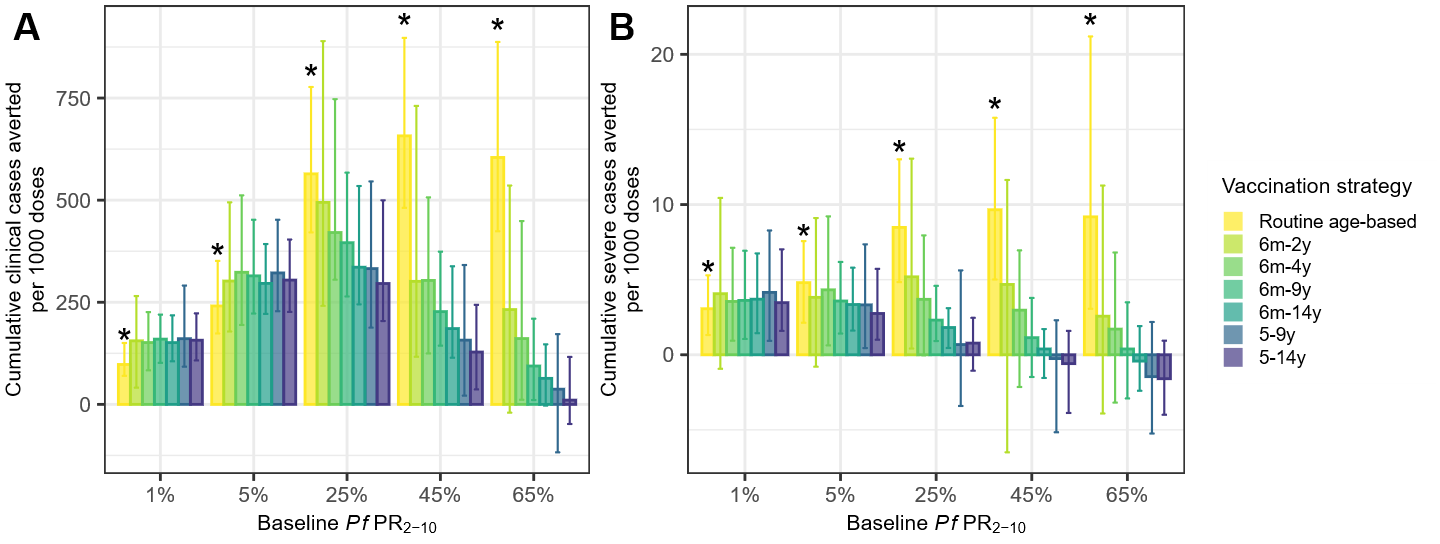
***

***
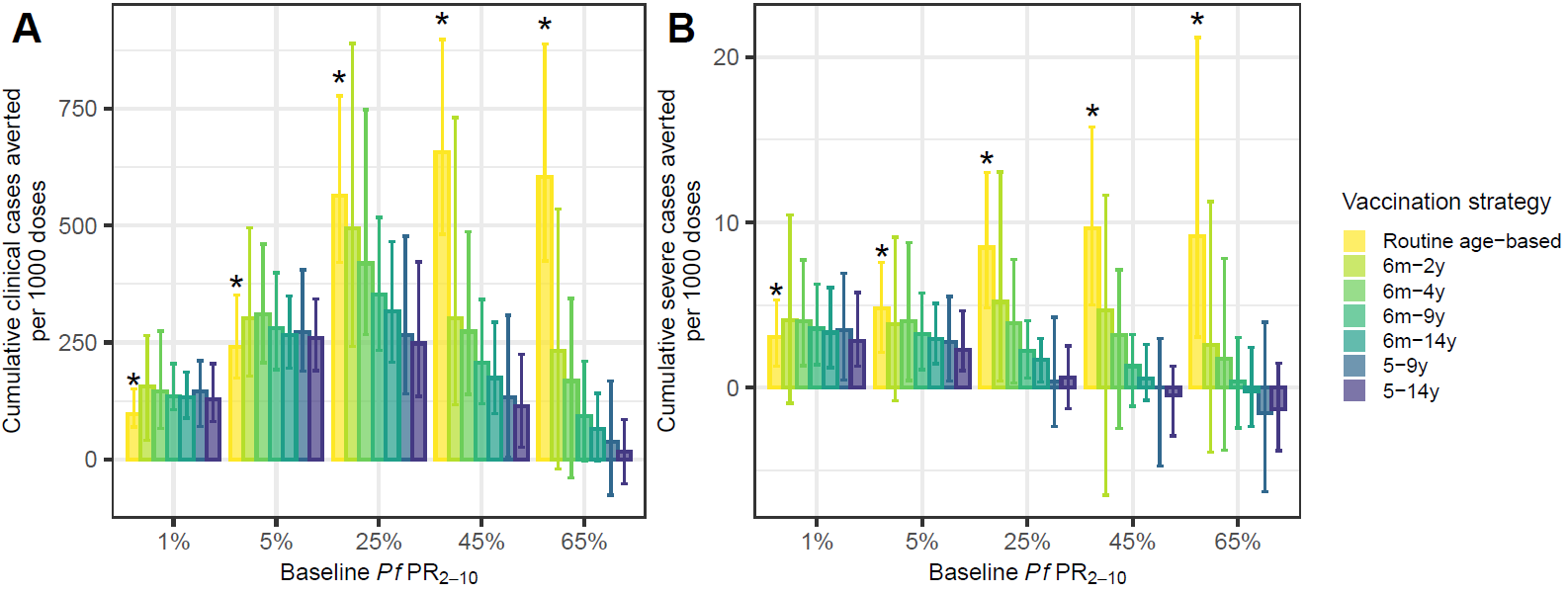
Figure S10. Catch-up campaign impact per 1000 additional doses assuming age-scaled antibody titres to 0.64 (top) and 0.4 (bottom), perennial setting: cumulative clinical (A) and severe (B) cases averted per 1000 additional doses over a 30-year simulation in perennial settings****. This figure is as per Fig 1 but assuming scaled antibody titres in children over 5 years of age. Values for routine age-based vaccination (bars with * above) show the outcome per 1000 doses delivered relative to no vaccination, while all other plotted strategies show additional outcomes averted per additional doses delivered relative to routine age-based vaccination. Routine age-based vaccination to 6-month-olds with supplementary catch-up vaccination in older children is assumed in all catch-up scenarios. The number of clinical or severe cases is compared to the routine age-based scenario baseline. The number of doses is calculated as the total number of doses under the specified vaccination strategy compared to a baseline scenario of routine age-based vaccination. The bars show median values, and the error bars show 95% credible intervals from 50 stochastic model runs. Note that the y-axes in plots A and B are different.*


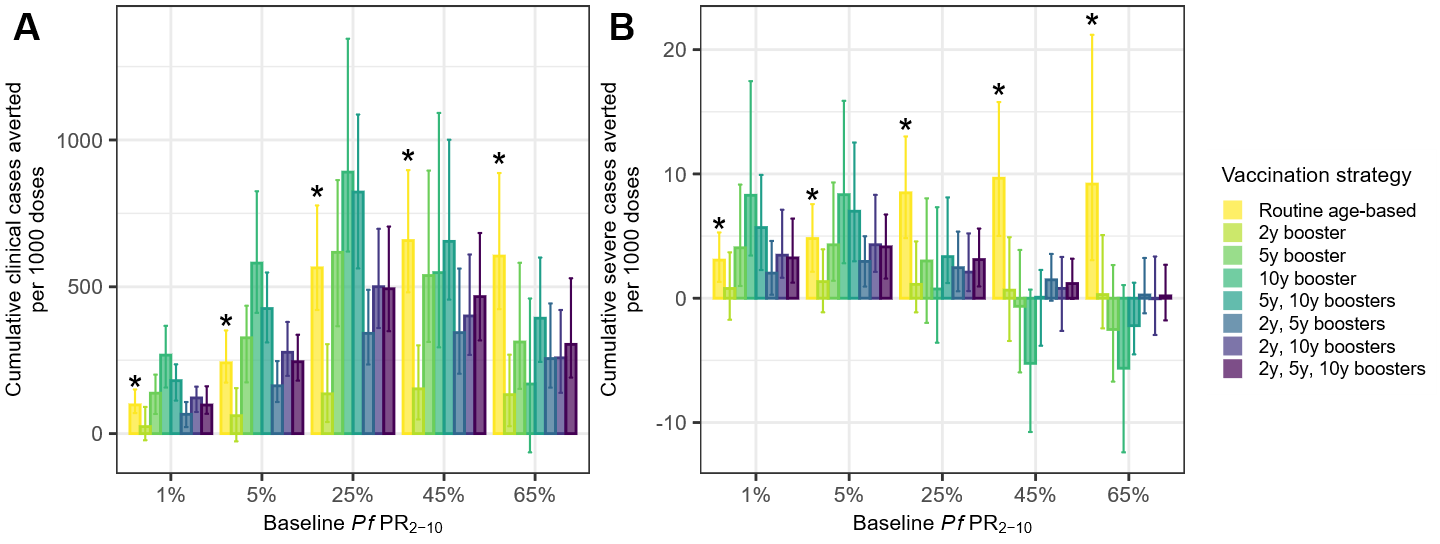


***
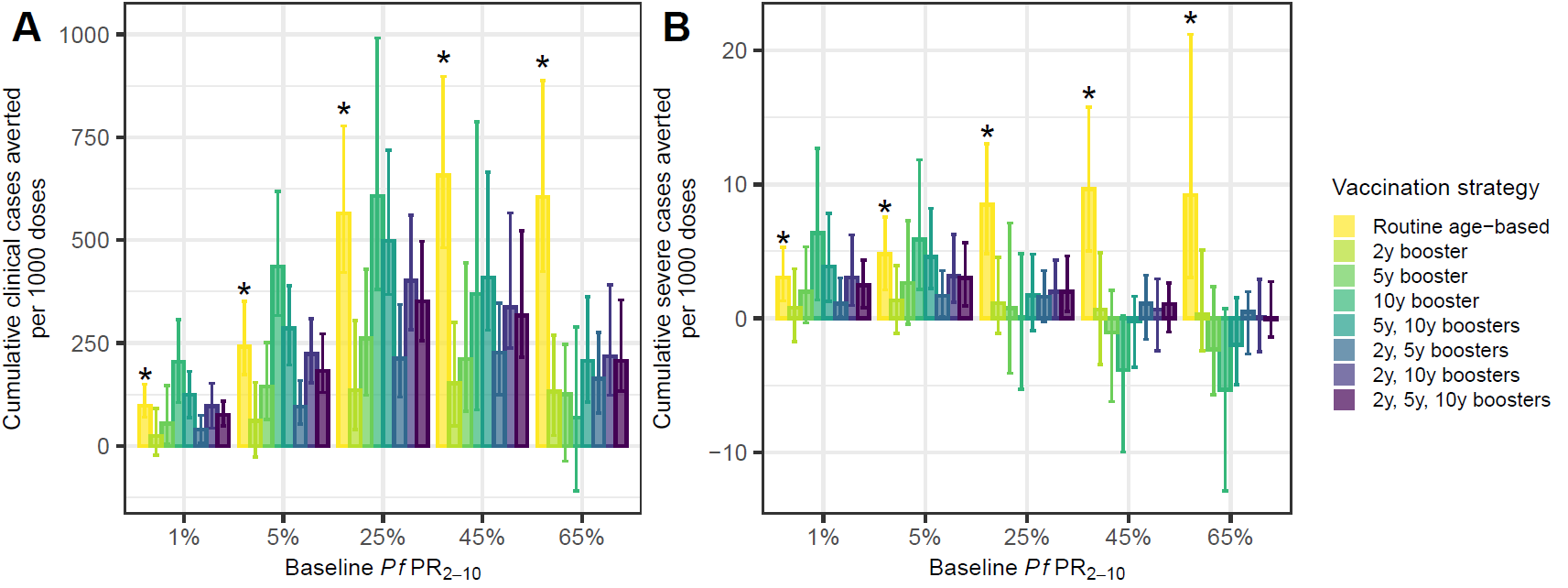
Figure S11. Extra booster impact per 1000 additional doses, assuming age-scaled antibody titres to 0.64 (top) and 0.4 (bottom), perennial setting: Cumulative clinical cases (A) and severe cases (B) averted per 1000 additional doses in perennial settings.*** *This figure is as per Fig 3, but assuming scaled antibody titres in children over 5 years of age. Values for routine age-based vaccination (bars with * above) show the outcome per 1000 doses delivered relative to no vaccination, while all other plotted strategies show additional outcomes averted per additional doses delivered relative to routine age-based vaccination. Outcomes are compared to a routine age-based vaccination baseline and are summarised over the final 15 years of the simulation so that each scenario had 15 years of continuous vaccination to young children before calculating cases averted. This allows for a fairer comparison between strategies with different booster dose timing. The bars show median values, and the error bars show 95% credible intervals of 50 stochastic model runs. Note that the y-axes in plots A and B are different.*

*Percent difference in outcomes averted by immunogenicity assumption*

Under the reduced immunogenicity assumptions, the scenarios where older children are vaccinated generally have lower impacts in terms of clinical and severe cases averted per person compared to scenarios with vaccination of younger children, but the difference is small, with median reductions of across all vaccination strategies and transmission settings of 4.3% and 2.1% for clinical and severe cases, respectively, when assuming a scaler of 0.64, and of 8.6% and 4.0% for clinical and severe cases, respectively, when assuming a scaler of 0.4. There are slightly larger median reductions for clinical and severe cases averted per additional dose of 16.1% and 15.0%, respectively, when assuming a scaler of 0.64, and 32.5% and 29.1%, respectively, when assuming a scaler of 0.4, relative to routine age-based vaccination (Figure S12).

The smallest differences between assumptions are seen in low and high transmission settings, while there is more often a significant difference between assumptions in transmission settings between 3% and 25% *Pf*PR_2-10_ (Fig S12). In high transmission settings, this is likely because of the high concentration of malaria burden in young children who are not affected by the lower immunogenicity assumption in older children. In low transmission settings, this is possibly because of wider confidence intervals due to low case numbers.

As expected, catch-up campaigns (Fig S12A) to children under 5 years and an extra booster dose at 2 years showed no difference between the scaled and non-scaled assumption because the difference in immunogenicity was only assumed for children over 5 years of age. However, even when catch-up vaccination was targeted to older children, there was no significant difference between the assumptions of no scaling and scaling to 0.64 except at 5% *Pf*PR_2-10_ when targeting children aged 6 months to 14 years. There was a significant difference between assumptions of no scaling and scaling to 0.4 when targeting children aged 6 months to 9 or to 14 years, and children aged 5-14 years in transmission settings of 3% to 25% *Pf*PR_2-10._

For extra booster doses, both assumptions of lower immunogenicity in children resulted in fewer cases averted in settings with at least 5% *Pf*PR_2-10_ and extra boosters at 5 years, 10 years, 5+10 years, and 2+5+10 years after the primary series compared to no scaling (Fig S12B). Additionally, the 2+10 years booster strategy averted significantly fewer cases in 5% to 45% *Pf*PR_2-10_ settings for the immunogenicity scaler of 0.4. Boosters at 2+10 years did not show a significant difference for the scaler of 0.64, though the median values indicated that the scaled assumption tended to avert fewer cases.

The combination strategies have a more complex pattern. Many scenarios did not show significant differences compared to the non-scaled assumption, though the median values were generally lower under the scaling assumption (Fig S12C). There was more likely to be a significant difference between scaled immunogenicity and non-scaled immunogenicity in moderate transmission settings and with more vaccination to older children, whether through catch-up campaigns or more extra booster doses.

***
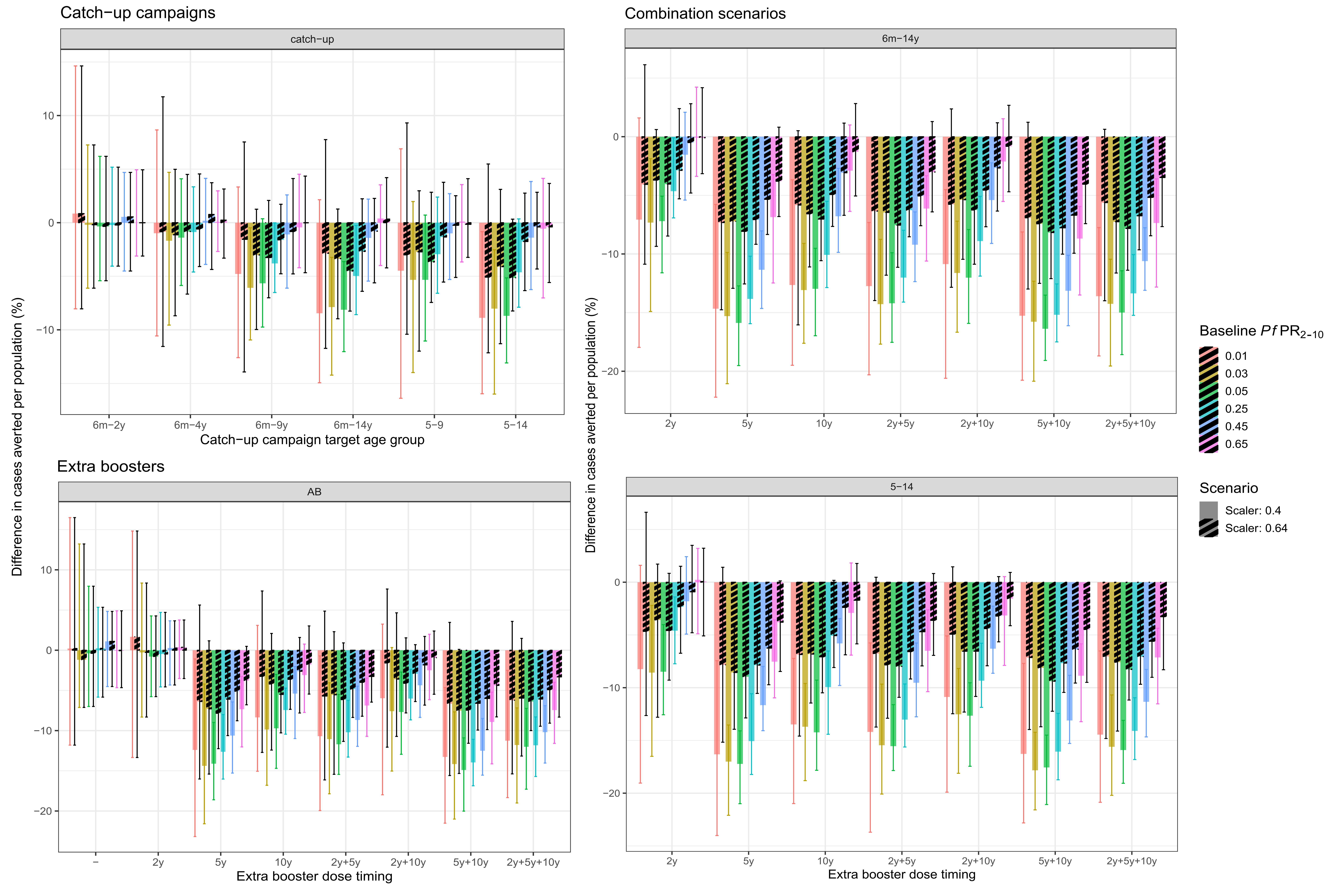
***

***Figure S12.* *Percent difference in cases averted per 1000 people between three different model assumptions****: 1) as presented in the main text, assuming that immunogenicity and efficacy of the vaccine are the same across age groups, and either 2) assuming that children 5 years of age and older have scaled immunogenicity of 0.64 compared to the fitted values (solid colours), or 3) assuming that children 5 years of age and older have scaled immunogenicity of 0.4 compared to the fitted values (black striped bars). Negative percent differences mean that model runs with assumption 1) had more cases averted. Bars represent medians, and error bars represent the 2.5% and 97.5% credible intervals across 50 stochastic parameter draws.*

*Efficiency frontiers under different immunogenicity assumptions*

The efficiency frontiers of the two different immunogenicity scaling assumptions (Figures S12-S13), show lower impact per dose overall, but the strategies falling on the efficiency frontier are broadly similar between immunogenicity assumptions, except in low transmission settings, a greater proportion of cases occur in children over 5 years old. When immunogenicity in older children is reduced, strategies including a booster dose at 2 years after the primary series are more likely to appear on the efficiency frontier (Table S7). Although reduced immunogenicity in older children decreases the number of clinical and severe cases averted per 1000 additional doses, our overall conclusion stands. Expanding vaccination to older children through catch-up campaigns or extra booster doses in high transmission settings is often more efficient than introducing routine vaccination in low transmission settings.

Although there is not a large difference in which strategies fall on the efficiency frontier in moderate to high transmission settings for clinical cases, nor for severe cases in any transmission settings, an important consideration is the relative cost-effectiveness of these vaccination strategies in comparison with other malaria control interventions.

Older children have higher cumulative exposure to malaria, especially those living in higher transmission settings, which may change their immune response to vaccination. There is some evidence that higher previous exposure to malaria reduces antibody immunogenicity and vaccine efficacy, but the relationship is not well-understood ^36^. However, prior exposure among children did not correlate to a difference in RTS,S/AS01-induced antibody immunogenicity, though evidence of maternal antibodies against malaria reduced RTS,S/AS01-induced antibody immunogenicity ^41^. The R21/Matrix-M phase III trial showed higher immunogenicity and vaccine efficacy in children aged 5-17 months compared to children aged 18-36 months ^7^. This sensitivity analysis shows the potential impact of a large decrease in vaccine-induced antibody titres in older age groups over a 30-year simulation.

***
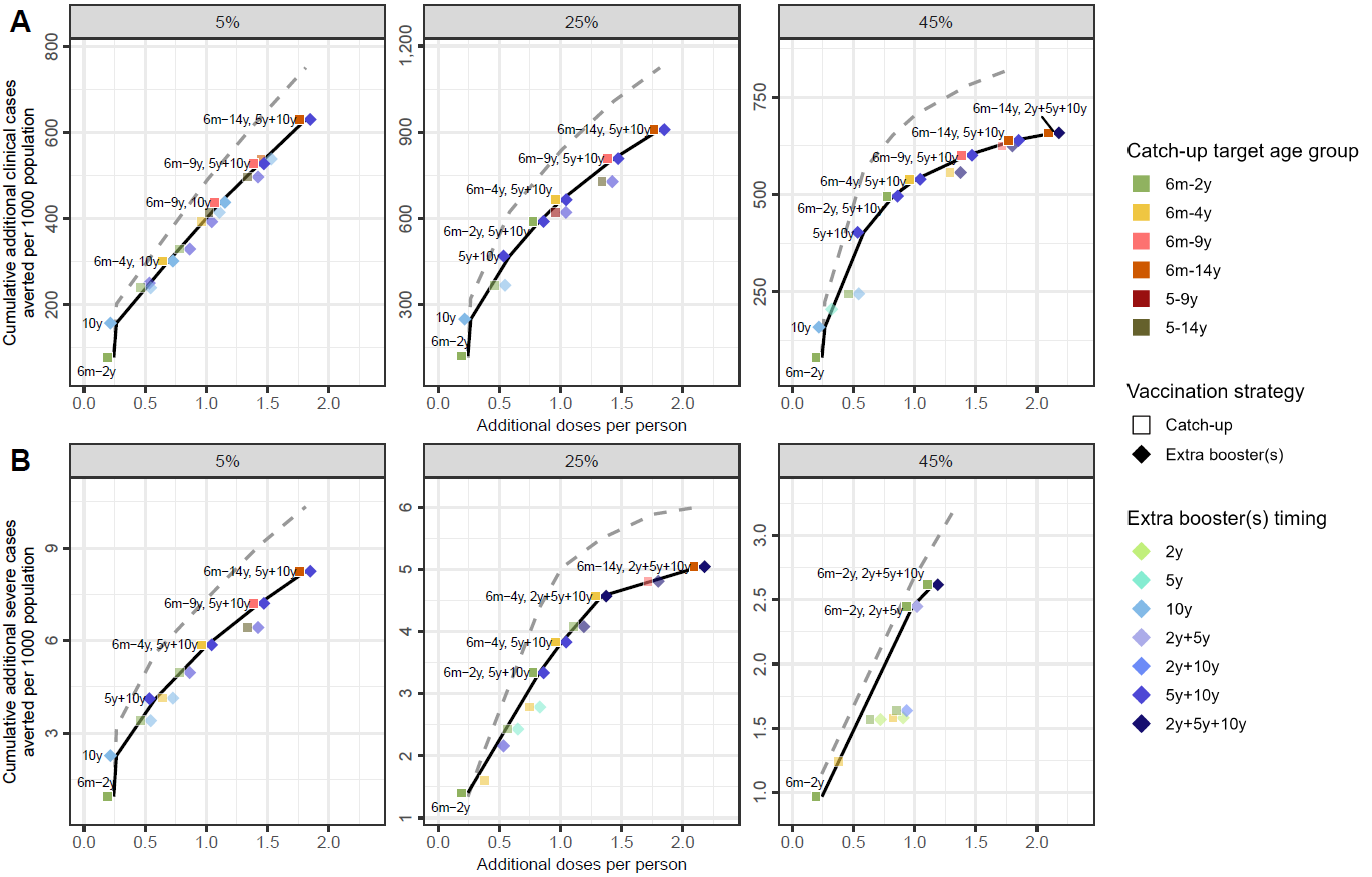
***

***Figure S13. Efficiency frontier for model runs with antibody dynamics in children 5 years of age and older, scaled by 0.64.*** *The dashed grey line is the efficiency frontier assuming no age-dependent immunogenicity or efficacy as presented in the main results. This figure is as per Fig 5, but assuming scaled antibody titres with a GMR of 0.64 relative to young children for children over 5 years of age.*

*
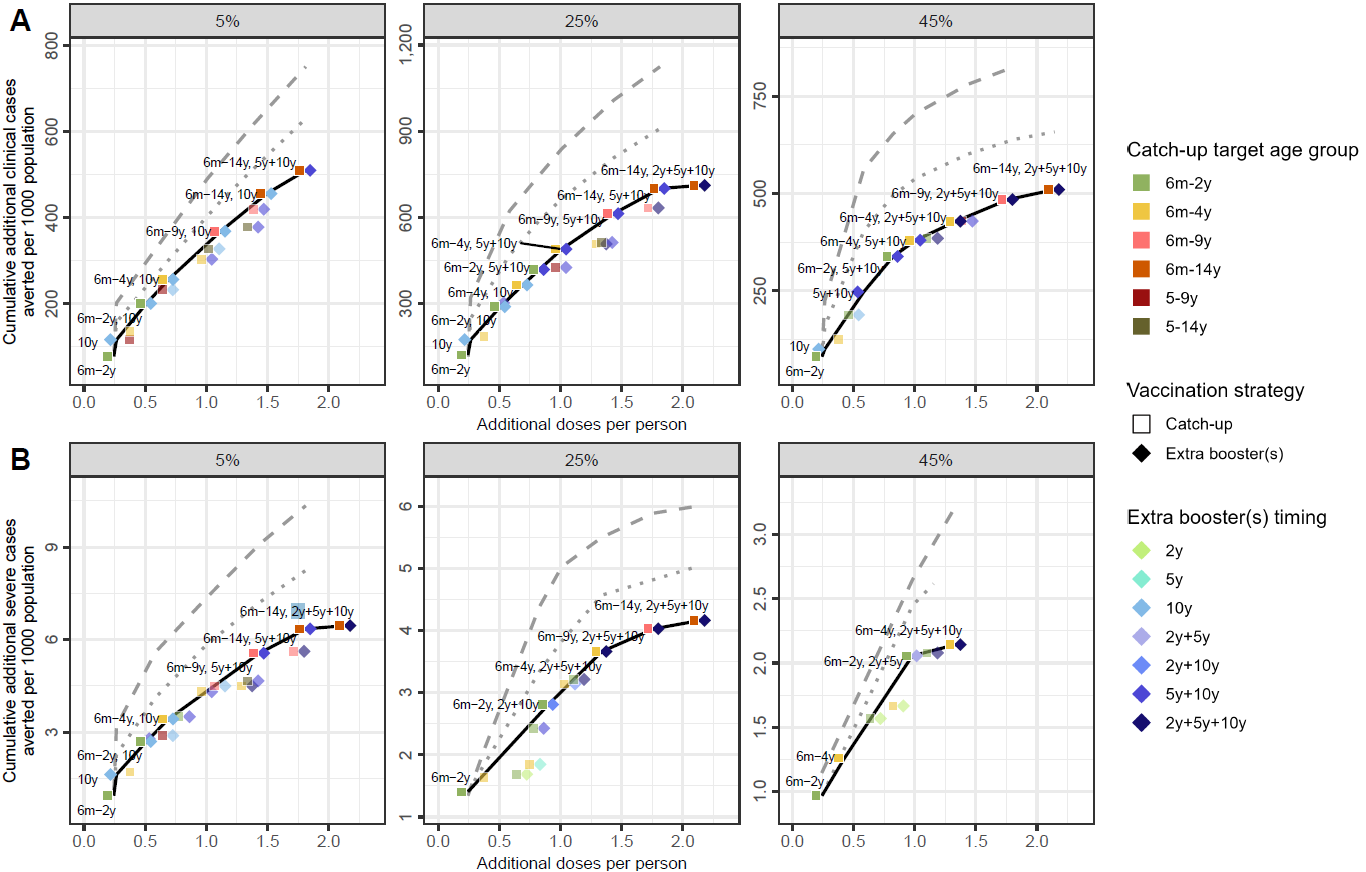
*

***Figure S14. Efficiency frontier for model runs with antibody dynamics in children 5 years of age and older scaled by 0.4.*** *The dashed grey line is the efficiency frontier assuming no age-dependent immunogenicity or efficacy as presented in the main results, and the dotted grey line is the efficiency frontier assuming age-dependent immunogenicity scaled by 0.64 as in Figure S13. This figure is as per Fig 5, but assuming scaled antibody titres with a GMR of 0.4 relative to young children over 5 years of age.*

***Table S7.*** *Comparison of strategies on the efficiency frontier under different immunogenicity assumptions, with respect to clinical and severe cases averted per population.*

| ***Pf*PR_2-10_** | **No Scaling** | **Scaled to 0.64** | **Scaled to 0.4** |
| --- | --- | --- | --- |
| **Clinical cases averted per 1000 population** | | | |
| 5% | 6m-2y, | 6m-2y, | 6m-2y, |
| 5% | 10y | 10y | 10y |
| 5% | 5-9y, 10y | 6m-4y, 10y | 6m-2y, 10y |
| 5% | 5-9y, 5y+10y | 6m-9y, 10y | 6m-4y, 10y |
| 5% | 5-14y, 5y+10y | 6m-9y, 5y+10y | 6m-9y, 10y |
| 5% | 6m-14y, 5y+10y | 6m-14y, 5y+10y | 6m-14y, 10y |
| 5% | - | - | 6m-14y, 5y+10y |
| 25% | 6m-2y, | 6m-2y, | 6m-2y, |
| 25% | 10y | 10y | 10y |
| 25% | 5y+10y | 5y+10y | 6m-2y, 10y |
| 25% | 6m-2y, 5y+10y | 6m-2y, 5y+10y | 6m-4y, 10y |
| 25% | 6m-4y, 5y+10y | 6m-4y, 5y+10y | 6m-2y, 5y+10y |
| 25% | 6m-9y, 5y+10y | 6m-9y, 5y+10y | 6m-4y, 5y+10y |
| 25% | 6m-14y, 5y+10y | 6m-14y, 5y+10y | 6m-9y, 5y+10y |
| 25% | - | - | 6m-14y, 5y+10y |
| 25% | - | - | 6m-14y, 2y+5y+10y |
| 45% | 6m-2y, | 6m-2y, | 6m-2y, |
| 45% | 10y | 10y | 10y |
| 45% | 5y+10y | 5y+10y | 5y+10y |
| 45% | 6m-2y, 5y+10y | 6m-2y, 5y+10y | 6m-2y, 5y+10y |
| 45% | 6m-4y, 5y+10y | 6m-4y, 5y+10y | 6m-4y, 5y+10y |
| 45% | 6m-9y, 5y+10y | 6m-9y, 5y+10y | 6m-4y, 2y+5y+10y |
| 45% | 6m-14y, 5y+10y | 6m-14y, 5y+10y | 6m-9y, 2y+5y+10y |
| 45% | - | 6m-14y, 2y+5y+10y | 6m-14y, 2y+5y+10y |
| **Severe cases averted per 1000 population** | | | |
| 5% | 6m-2y, | 6m-2y, | 6m-2y, |
| 5% | 10y | 10y | 10y |
| 5% | 6m-2y, 10y | 5y+10y | 5y+10y |
| 5% | 6m-4y, 10y | 6m-4y, 5y+10y | 6m-4y, 5y+10y |
| 5% | 6m-9y, 5y+10y | 6m-9y, 5y+10y | 6m-9y, 5y+10y |
| 5% | 6m-14y, 5y+10y | 6m-14y, 5y+10y | 6m-14y, 5y+10y |
| 5% | 6m-14y, 2y+5y+10y | NA | NA |
| 25% | 6m-2y, | 6m-2y, | 6m-2y, |
| 25% | 6m-2y, 2y+10y | 5y | 6m-2y, 5y+10y |
| 25% | 6m-4y, 2y+5y+10y | 6m-2y, 5y+10y | 6m-4y, 5y+10y |
| 25% | 6m-9y, 2y+5y+10y | 6m-4y, 5y+10y | 6m-4y, 2y+5y+10y |
| 25% | 6m-14y, 2y+5y+10y | 6m-4y, 2y+5y+10y | 6m-14y, 2y+5y+10y |
| 25% | - | 6m-9y, 2y+5y+10y | - |
| 25% | - | 6m-14y, 2y+5y+10y | - |
| 45% | 6m-2y, | 6m-2y, | 6m-2y, |
| 45% | 6m-4y, | 6m-2y, 2y+5y | 6m-2y, 2y+5y |
| 45% | 6m-2y, 2y+5y | 6m-4y, 2y+5y+10y | 6m-2y, 2y+5y+10y |
| 45% | 6m-4y, 2y+5y+10y | - | - |

***Table S8. Outcomes averted per 1000 people, per 1000 additional doses, and per 1000 total doses in a perennial setting, assuming antibody titres in children 5 years and over are scaled to 0.64.*** *This table is as per Table 2, but assuming scaled antibody titres with a GMR of 0.64 in children over 5 years of age. Strategies included in the table are either catch-up vaccination or additional boosters, but not a combination of catch-up and additional boosters, and are summarised over the total population and a 30-year time horizon. Clinical and severe cases averted per 1000 people and per 1000 doses are compared to a baseline of no vaccination, while additional clinical and severe cases averted per 1000 additional doses of catch-up or extra booster strategies are compared to a baseline of routine age-based vaccination. Each is supplementary to routine age-based vaccination that includes a single booster dose at 12 months post-primary series. The age groups listed are those targeted for a catch-up vaccination campaign (e.g. 6m-14y refers to a catch-up vaccination campaign to children between 6 months and 14 years at vaccine introduction that is supplementary to continuous routine age-based vaccination). Booster dose timing (e.g. 10y booster) is noted as the timing of additional booster dose(s) (e.g. 10y booster refers to routine age-based vaccination with a single additional booster at 10 years after the primary series, so this strategy has two booster doses, the standard one at 12 months plus the additional one at 10 years). The table is grouped by transmission intensity. Median values with 95% credible intervals of 50 stochastic model runs are presented.*

| **Strategy** | **Clinical cases averted per 1000 population** | **Severe cases averted per 1000 population** | **Additional clinical cases averted per 1000 additional doses (relative to routine age-based)** | **Additional severe cases averted per 1000 additional doses (relative to routine age-based)** | **Clinical cases averted per 1000 doses**  **(relative to no vaccination)** | **Severe cases averted per 1000 doses (relative to no vaccination)** |
| --- | --- | --- | --- | --- | --- | --- |
| **Low transmission: *Pf*PR_2-10_ = 5%** | | | | | | |
| Routine age-based | 714 (515, 1041) | 14 (6, 22) | Ref | Ref | 241 (174, 351) | 5 (2, 8) |
| 2y booster | 746 (537, 1092) | 15 (7, 23) | 61 (-27, 155) | 1 (-1, 4) | 219 (158, 321) | 4 (2, 7) |
| 5y booster | 833 (594, 1195) | 16 (7, 25) | 326 (174, 436) | 4 (1, 9) | 250 (178, 358) | 5 (2, 8) |
| 10y booster | 863 (645, 1218) | 17 (7, 26) | 581 (411, 825) | 8 (3, 16) | 267 (199, 377) | 5 (2, 8) |
| 5y, 10y boosters | 956 (704, 1365) | 19 (8, 29) | 426 (311, 549) | 7 (3, 13) | 269 (198, 384) | 5 (2, 8) |
| 2y, 5y boosters | 835 (603, 1233) | 17 (8, 25) | 163 (107, 247) | 3 (1, 5) | 225 (163, 332) | 4 (2, 7) |
| 2y, 10y boosters | 900 (648, 1287) | 17 (8, 27) | 277 (197, 380) | 4 (2, 8) | 248 (179, 355) | 5 (2, 8) |
| 2y, 5y, 10y boosters | 930 (684, 1346) | 18 (8, 29) | 245 (180, 337) | 4 (2, 7) | 240 (176, 347) | 5 (2, 7) |
| 6m-2y | 797 (573, 1156) | 15 (7, 24) | 302 (178, 495) | 4 (-1, 9) | 248 (178, 361) | 5 (2, 8) |
| 6m-4y | 847 (611, 1261) | 16 (8, 26) | 323 (194, 512) | 4 (1, 9) | 250 (180, 373) | 5 (2, 8) |
| 6m-9y | 963 (720, 1420) | 17 (8, 27) | 315 (222, 452) | 4 (1, 6) | 252 (189, 372) | 5 (2, 7) |
| 6m-14y | 1075 (800, 1516) | 19 (8, 28) | 296 (222, 393) | 3 (2, 6) | 256 (191, 362) | 4 (2, 7) |
| 5-9y | 854 (616, 1210) | 16 (7, 25) | 322 (228, 452) | 3 (0, 7) | 252 (182, 357) | 5 (2, 7) |
| 5-14y | 961 (703, 1350) | 17 (8, 26) | 304 (226, 404) | 3 (1, 6) | 255 (187, 358) | 4 (2, 7) |
| **Moderate transmission: *Pf*PR_2-10_ = 25%** | | | | | | |
| Routine age-based | 1669 (1248, 2301) | 25 (14, 39) | Ref | Ref | 564 (421, 777) | 8 (5, 13) |
| 2y booster | 1746 (1289, 2385) | 26 (14, 38) | 135 (39, 304) | 1 (-1, 5) | 513 (378, 702) | 8 (4, 11) |
| 5y booster | 1904 (1415, 2570) | 27 (15, 40) | 617 (366, 863) | 3 (-2, 8) | 571 (425, 771) | 8 (4, 12) |
| 10y booster | 1944 (1420, 2580) | 26 (14, 39) | 890 (620, 1345) | 1 (-4, 7) | 603 (439, 801) | 8 (4, 12) |
| 5y, 10y boosters | 2170 (1578, 2822) | 28 (16, 42) | 823 (563, 1087) | 3 (1, 8) | 612 (445, 796) | 8 (4, 12) |
| 2y, 5y boosters | 1935 (1433, 2618) | 27 (16, 41) | 341 (235, 490) | 2 (1, 5) | 523 (387, 708) | 7 (4, 11) |
| 2y, 10y boosters | 2025 (1480, 2670) | 27 (15, 39) | 500 (359, 698) | 2 (1, 5) | 560 (408, 739) | 8 (4, 11) |
| 2y, 5y, 10y boosters | 2151 (1572, 2868) | 28 (16, 41) | 493 (349, 705) | 3 (1, 6) | 555 (405, 741) | 7 (4, 11) |
| 6m-2y | 1803 (1328, 2450) | 27 (15, 40) | 494 (241, 889) | 5 (0, 13) | 564 (415, 765) | 8 (5, 13) |
| 6m-4y | 1887 (1386, 2554) | 27 (15, 40) | 421 (305, 747) | 4 (0, 8) | 558 (410, 753) | 8 (5, 12) |
| 6m-9y | 2030 (1482, 2791) | 27 (15, 42) | 396 (264, 567) | 2 (1, 5) | 533 (389, 733) | 7 (4, 11) |
| 6m-14y | 2117 (1580, 2951) | 27 (16, 41) | 336 (245, 535) | 2 (0, 3) | 505 (377, 704) | 7 (4, 10) |
| 5-9y | 1833 (1364, 2475) | 25 (14, 38) | 332 (188, 546) | 1 (-3, 6) | 541 (403, 731) | 7 (4, 11) |
| 5-14y | 1941 (1431, 2695) | 26 (15, 39) | 296 (204, 500) | 1 (-1, 2) | 516 (380, 716) | 7 (4, 10) |
| **Moderately high transmission: *Pf*PR_2-10_ = 45%** | | | | | | |
| Routine age-based | 1945 (1420, 2657) | 29 (15, 47) | Ref | Ref | 657 (481, 897) | 10 (5, 16) |
| 2y booster | 2023 (1451, 2775) | 29 (14, 46) | 152 (48, 300) | 1 (-3, 5) | 594 (426, 814) | 8 (4, 13) |
| 5y booster | 2154 (1549, 2960) | 28 (14, 45) | 538 (312, 896) | -1 (-6, 4) | 648 (465, 889) | 9 (4, 14) |
| 10y booster | 2106 (1521, 2866) | 27 (14, 45) | 548 (294, 1092) | -5 (-11, 1) | 653 (472, 889) | 8 (4, 14) |
| 5y, 10y boosters | 2356 (1691, 3179) | 29 (15, 45) | 655 (456, 1001) | 0 (-4, 2) | 664 (478, 899) | 8 (4, 13) |
| 2y, 5y boosters | 2208 (1581, 3053) | 29 (15, 47) | 344 (204, 562) | 1 (0, 4) | 596 (427, 827) | 8 (4, 13) |
| 2y, 10y boosters | 2227 (1598, 3035) | 29 (15, 48) | 401 (268, 610) | 1 (-3, 3) | 616 (442, 838) | 8 (4, 13) |
| 2y, 5y, 10y boosters | 2369 (1712, 3259) | 30 (16, 48) | 466 (317, 683) | 1 (0, 3) | 612 (443, 843) | 8 (4, 12) |
| 6m-2y | 2024 (1481, 2802) | 29 (15, 48) | 301 (117, 731) | 5 (-6, 12) | 632 (462, 877) | 9 (5, 15) |
| 6m-4y | 2077 (1509, 2831) | 29 (14, 48) | 303 (125, 507) | 3 (-2, 7) | 612 (447, 836) | 9 (4, 14) |
| 6m-9y | 2154 (1554, 2959) | 30 (15, 47) | 227 (144, 374) | 1 (-1, 4) | 565 (408, 776) | 8 (4, 12) |
| 6m-14y | 2176 (1572, 3023) | 29 (14, 46) | 186 (114, 338) | 0 (-2, 2) | 518 (375, 722) | 7 (3, 11) |
| 5-9y | 2012 (1459, 2753) | 28 (14, 46) | 157 (21, 341) | 0 (-5, 2) | 594 (431, 813) | 8 (4, 14) |
| 5-14y | 2054 (1500, 2837) | 28 (14, 45) | 128 (37, 244) | -1 (-4, 2) | 545 (399, 754) | 7 (4, 12) |
| **High transmission: *Pf*PR_2-10_ = 65%** | | | | | | |
| Routine age-based | 1789 (1255, 2626) | 27 (9, 63) | Ref | Ref | 605 (424, 887) | 9 (3, 21) |
| 2y booster | 1849 (1297, 2701) | 28 (9, 62) | 133 (25, 269) | 0 (-2, 5) | 543 (382, 795) | 8 (3, 18) |
| 5y booster | 1912 (1342, 2764) | 26 (9, 61) | 312 (152, 582) | -3 (-7, 3) | 575 (403, 829) | 8 (3, 18) |
| 10y booster | 1817 (1311, 2668) | 25 (8, 62) | 169 (-64, 460) | -6 (-12, 1) | 564 (407, 828) | 8 (2, 19) |
| 5y, 10y boosters | 1990 (1418, 2939) | 25 (8, 61) | 393 (244, 600) | -2 (-5, 1) | 562 (400, 830) | 7 (2, 17) |
| 2y, 5y boosters | 1969 (1412, 2918) | 27 (9, 64) | 256 (157, 443) | 0 (-1, 3) | 531 (382, 790) | 7 (3, 17) |
| 2y, 10y boosters | 1959 (1379, 2899) | 27 (9, 64) | 258 (139, 421) | 0 (-3, 3) | 542 (382, 803) | 8 (3, 18) |
| 2y, 5y, 10y boosters | 2055 (1437, 3044) | 27 (9, 63) | 304 (191, 529) | 0 (-2, 3) | 532 (372, 788) | 7 (2, 16) |
| 6m-2y | 1855 (1293, 2686) | 28 (9, 64) | 232 (-20, 536) | 3 (-4, 11) | 581 (404, 839) | 9 (3, 20) |
| 6m-4y | 1864 (1298, 2734) | 27 (9, 65) | 161 (11, 449) | 2 (-3, 7) | 551 (384, 810) | 8 (3, 19) |
| 6m-9y | 1852 (1302, 2770) | 27 (8, 64) | 94 (11, 210) | 0 (-3, 3) | 486 (342, 728) | 7 (2, 17) |
| 6m-14y | 1857 (1267, 2740) | 27 (7, 64) | 64 (-3, 147) | 0 (-2, 2) | 443 (302, 655) | 6 (2, 15) |
| 5-9y | 1807 (1277, 2655) | 26 (9, 62) | 37 (-117, 172) | -1 (-5, 2) | 533 (378, 785) | 8 (3, 18) |
| 5-14y | 1778 (1257, 2664) | 27 (8, 61) | 10 (-48, 116) | -2 (-4, 1) | 472 (334, 710) | 7 (2, 16) |

***Table S9. Outcomes averted per 1000 people, per 1000 additional doses, and per 1000 total doses in a perennial setting, assuming antibody titres in children 5 years and over are scaled to 0.4.*** *This table is as per Table 2, but assuming scaled antibody titres with a GMR of 0.64 in children over 5 years of age. Strategies included in the table are either catch-up vaccination or additional boosters, but not a combination of catch-up and additional boosters, and are summarised over the total population and a 30-year time horizon. Clinical and severe cases averted per 1000 people and per 1000 doses are compared to a baseline of no vaccination, while additional clinical and severe cases averted per 1000 additional doses of catch-up or extra booster strategies are compared to a baseline of routine age-based vaccination. Each is supplementary to routine age-based vaccination that includes a single booster dose at 12 months post-primary series. The age groups listed are those targeted for a catch-up vaccination campaign (e.g. 6m-14y refers to a catch-up vaccination campaign to children between 6 months and 14 years at vaccine introduction that is supplementary to continuous routine age-based vaccination). Booster dose timing (e.g. 10y booster) is noted as the timing of additional booster dose(s) (e.g. 10y booster refers to routine age-based vaccination with a single additional booster at 10 years after the primary series, so this strategy has two booster doses, the standard one at 12 months plus the additional one at 10 years). The table is grouped by transmission intensity. Median values with 95% credible intervals of 50 stochastic model runs are presented.*

| **Strategy** | **Clinical cases averted per 1000 population** | **Severe cases averted per 1000 population** | **Additional clinical cases averted per 1000 additional doses (relative to routine age-based)** | **Additional severe cases averted per 1000 additional doses (relative to routine age-based)** | **Clinical cases averted per 1000 doses**  **(relative to no vaccination)** | **Severe cases averted per 1000 doses (relative to no vaccination)** |
| --- | --- | --- | --- | --- | --- | --- |
| **Low transmission: *Pf*PR_2-10_ = 5%** | | | | | | |
| Routine age-based | 714 (515, 1041) | 14 (6, 22) | Ref | Ref | 241 (174, 351) | 5 (2, 8) |
| 2y booster | 746 (537, 1092) | 15 (7, 23) | 61 (-27, 155) | 1 (-1, 4) | 219 (158, 321) | 4 (2, 7) |
| 5y booster | 764 (559, 1128) | 15 (7, 24) | 145 (64, 251) | 3 (0, 7) | 229 (168, 338) | 5 (2, 7) |
| 10y booster | 824 (610, 1193) | 16 (7, 25) | 436 (317, 618) | 6 (2, 12) | 254 (189, 369) | 5 (2, 8) |
| 5y, 10y boosters | 878 (649, 1263) | 17 (8, 27) | 286 (197, 390) | 5 (2, 8) | 247 (183, 356) | 5 (2, 8) |
| 2y, 5y boosters | 788 (571, 1154) | 15 (7, 24) | 95 (54, 159) | 2 (0, 4) | 212 (154, 311) | 4 (2, 7) |
| 2y, 10y boosters | 864 (622, 1228) | 17 (7, 26) | 224 (153, 309) | 3 (1, 6) | 239 (172, 339) | 5 (2, 7) |
| 2y, 5y, 10y boosters | 886 (636, 1251) | 17 (7, 27) | 182 (130, 272) | 3 (1, 6) | 228 (164, 322) | 4 (2, 7) |
| 6m-2y | 797 (573, 1156) | 15 (7, 24) | 302 (178, 495) | 4 (-1, 9) | 248 (178, 361) | 5 (2, 8) |
| 6m-4y | 846 (616, 1226) | 16 (8, 25) | 310 (206, 460) | 4 (0, 9) | 249 (182, 362) | 5 (2, 7) |
| 6m-9y | 950 (701, 1371) | 17 (8, 27) | 281 (192, 399) | 3 (1, 6) | 249 (184, 360) | 5 (2, 7) |
| 6m-14y | 1044 (770, 1462) | 18 (8, 28) | 266 (195, 349) | 3 (1, 5) | 249 (184, 349) | 4 (2, 7) |
| 5-9y | 832 (601, 1189) | 16 (7, 24) | 273 (188, 405) | 3 (0, 6) | 245 (177, 352) | 5 (2, 7) |
| 5-14y | 917 (680, 1309) | 17 (7, 25) | 259 (190, 343) | 2 (1, 5) | 243 (180, 347) | 4 (2, 7) |
| **Moderate transmission: *Pf*PR_2-10_ = 25%** | | | | | | |
| Routine age-based | 1669 (1248, 2301) | 25 (14, 39) | Ref | Ref | 564 (421, 777) | 8 (5, 13) |
| 2y booster | 1746 (1289, 2385) | 26 (14, 38) | 135 (39, 304) | 1 (-1, 5) | 513 (378, 702) | 8 (4, 11) |
| 5y booster | 1793 (1324, 2395) | 25 (14, 39) | 261 (123, 429) | 1 (-4, 7) | 539 (396, 720) | 8 (4, 12) |
| 10y booster | 1865 (1364, 2476) | 25 (14, 39) | 607 (380, 990) | 0 (-5, 5) | 580 (423, 768) | 8 (4, 12) |
| 5y, 10y boosters | 1983 (1482, 2666) | 27 (15, 40) | 498 (368, 718) | 2 (-1, 5) | 560 (418, 752) | 8 (4, 11) |
| 2y, 5y boosters | 1837 (1351, 2519) | 27 (15, 39) | 213 (118, 344) | 2 (0, 4) | 495 (365, 681) | 7 (4, 11) |
| 2y, 10y boosters | 1966 (1427, 2614) | 27 (15, 39) | 402 (282, 560) | 2 (0, 4) | 544 (394, 724) | 7 (4, 11) |
| 2y, 5y, 10y boosters | 2001 (1478, 2722) | 27 (15, 41) | 351 (256, 497) | 2 (1, 5) | 515 (381, 705) | 7 (4, 11) |
| 6m-2y | 1803 (1328, 2450) | 27 (15, 40) | 494 (241, 889) | 5 (0, 13) | 564 (415, 765) | 8 (5, 13) |
| 6m-4y | 1880 (1373, 2587) | 27 (15, 41) | 419 (267, 747) | 4 (0, 8) | 556 (406, 763) | 8 (4, 12) |
| 6m-9y | 1967 (1464, 2715) | 27 (15, 41) | 352 (233, 517) | 2 (1, 4) | 516 (384, 712) | 7 (4, 11) |
| 6m-14y | 2068 (1536, 2849) | 27 (15, 42) | 317 (207, 465) | 2 (0, 3) | 494 (367, 679) | 6 (4, 10) |
| 5-9y | 1815 (1323, 2440) | 25 (14, 39) | 267 (141, 477) | 0 (-2, 4) | 536 (391, 720) | 8 (4, 12) |
| 5-14y | 1899 (1382, 2609) | 26 (15, 39) | 248 (135, 422) | 1 (-1, 3) | 505 (368, 693) | 7 (4, 10) |
| **Moderately high transmission: *Pf*PR_2-10_ = 45%** | | | | | | |
| Routine age-based | 1945 (1420, 2657) | 29 (15, 47) | Ref | Ref | 657 (481, 897) | 10 (5, 16) |
| 2y booster | 2023 (1451, 2775) | 29 (14, 46) | 152 (48, 300) | 1 (-3, 5) | 594 (426, 814) | 8 (4, 13) |
| 5y booster | 2036 (1478, 2779) | 27 (14, 45) | 211 (85, 445) | -1 (-6, 2) | 610 (444, 835) | 8 (4, 14) |
| 10y booster | 2040 (1500, 2800) | 28 (14, 45) | 369 (88, 788) | -4 (-10, 0) | 633 (465, 868) | 9 (4, 14) |
| 5y, 10y boosters | 2210 (1586, 3016) | 28 (15, 45) | 409 (282, 665) | 0 (-4, 2) | 624 (448, 852) | 8 (4, 13) |
| 2y, 5y boosters | 2121 (1534, 2891) | 29 (15, 47) | 227 (125, 347) | 1 (-2, 3) | 573 (414, 783) | 8 (4, 13) |
| 2y, 10y boosters | 2181 (1596, 3012) | 29 (15, 45) | 337 (238, 566) | 1 (-2, 3) | 603 (442, 833) | 8 (4, 13) |
| 2y, 5y, 10y boosters | 2242 (1630, 3100) | 30 (16, 47) | 317 (216, 522) | 1 (-1, 3) | 579 (421, 802) | 8 (4, 12) |
| 6m-2y | 2024 (1481, 2802) | 29 (15, 48) | 301 (117, 731) | 5 (-6, 12) | 632 (462, 877) | 9 (5, 15) |
| 6m-4y | 2076 (1497, 2840) | 30 (14, 48) | 273 (139, 487) | 3 (-2, 7) | 613 (442, 839) | 9 (4, 14) |
| 6m-9y | 2114 (1535, 2963) | 29 (14, 47) | 207 (119, 342) | 1 (-1, 3) | 554 (402, 778) | 8 (4, 12) |
| 6m-14y | 2178 (1564, 2991) | 29 (14, 48) | 174 (98, 293) | 1 (-1, 3) | 520 (374, 713) | 7 (3, 11) |
| 5-9y | 1991 (1461, 2739) | 28 (15, 47) | 133 (5, 308) | 0 (-5, 3) | 588 (431, 809) | 8 (4, 14) |
| 5-14y | 2025 (1474, 2785) | 28 (14, 45) | 114 (26, 225) | 0 (-3, 1) | 537 (392, 741) | 7 (4, 12) |
| **High transmission: *Pf*PR_2-10_ = 65%** | | | | | | |
| Routine age-based | 1789 (1255, 2626) | 27 (9, 63) | Ref | Ref | 605 (424, 887) | 9 (3, 21) |
| 2y booster | 1849 (1297, 2701) | 28 (9, 62) | 133 (25, 269) | 0 (-2, 5) | 543 (382, 795) | 8 (3, 18) |
| 5y booster | 1845 (1280, 2696) | 26 (9, 61) | 126 (-37, 247) | -2 (-6, 2) | 555 (384, 811) | 8 (3, 18) |
| 10y booster | 1833 (1255, 2672) | 25 (7, 61) | 69 (-109, 289) | -5 (-13, 1) | 568 (389, 830) | 8 (2, 19) |
| 5y, 10y boosters | 1919 (1336, 2816) | 26 (9, 62) | 207 (106, 362) | -2 (-5, 2) | 542 (377, 795) | 7 (3, 17) |
| 2y, 5y boosters | 1903 (1348, 2789) | 27 (9, 64) | 163 (80, 276) | 0 (-3, 2) | 514 (364, 755) | 7 (2, 17) |
| 2y, 10y boosters | 1934 (1346, 2839) | 27 (10, 64) | 218 (124, 392) | 0 (-2, 3) | 535 (372, 788) | 7 (3, 18) |
| 2y, 5y, 10y boosters | 1990 (1412, 2908) | 28 (9, 64) | 207 (134, 355) | 0 (-1, 3) | 514 (365, 752) | 7 (2, 16) |
| 6m-2y | 1855 (1293, 2686) | 28 (9, 64) | 232 (-20, 536) | 3 (-4, 11) | 581 (404, 839) | 9 (3, 20) |
| 6m-4y | 1848 (1303, 2711) | 28 (9, 65) | 168 (-39, 344) | 2 (-4, 8) | 547 (385, 803) | 8 (3, 19) |
| 6m-9y | 1867 (1293, 2727) | 28 (10, 64) | 92 (-3, 210) | 0 (-2, 3) | 490 (339, 716) | 7 (3, 17) |
| 6m-14y | 1855 (1273, 2748) | 27 (8, 64) | 65 (-2, 141) | 0 (-2, 2) | 442 (304, 656) | 7 (2, 15) |
| 5-9y | 1783 (1285, 2640) | 27 (9, 62) | 38 (-76, 168) | -2 (-6, 4) | 527 (381, 781) | 8 (3, 18) |
| 5-14y | 1789 (1239, 2687) | 26 (8, 62) | 16 (-52, 86) | -1 (-4, 1) | 476 (330, 716) | 7 (2, 16) |

# Incremental Efficiency analysis

## Extended methods

An efficiency frontier compares the benefits of an intervention against its cost, with non-dominated strategies, or those where no alternative is both cheaper and equally or more effective, falling on the frontier ^42^. The strategies on the frontier represent efficient use of resources, as the same benefits would require higher costs.

In the main text, we present the efficiency frontier (Figure 5), with the additional number of R21 doses delivered as a proxy for cost, and the additional number of cases averted per 1000 people as a proxy for benefit, both compared to a routine age-based strategy over the 30-year simulation and assuming the benefit scales proportionally with the cost. Additional doses were calculated by subtracting the number of doses in the routine age-based strategy from the number of doses required for the other vaccination scenarios for each draw, then summarising over the parameter draws to get the median and 2.5% and 97.5% quantiles. Additional cases averted were calculated similarly, by subtracting the number of cases over the simulation for each scenario from the number of cases from the routine age-based scenario. The difference between the routine age-based and additional vaccine scenarios is solely in the delivery of extra doses of R21/Matrix-M as either extra booster doses or catch-up campaigns.

With these summarised metrics, we calculated incremental cost-effectiveness ratios (ICER), the incremental cost of choosing each strategy over the next most costly strategy (in additional doses per person relative to routine age-based vaccination) divided by its incremental effectiveness (in terms of clinical or severe cases averted per 1000 people) using the {dampack} R package (v 1.0.1) ^43^. Efficiency frontiers were plotted for each transmission and seasonality setting.

Strongly dominated strategies, those with both fewer clinical or severe cases averted and higher costs (in additional doses), were excluded from the visualisations (Figures 5, S8, S13, S14). Extended (weakly) dominated scenarios, in which a linear combination of two other strategies could yield a higher benefit with fewer doses, were retained for clarity, but do not lie on the efficiency frontier.

## Expected loss

Despite their usefulness, the efficiency frontiers ignore the wide uncertainty intervals, many of which overlap, in both metrics, and do not allow comparison across different willingness-to-pay (WTP) thresholds. As a sensitivity analysis to account for uncertainty in the number of additional cases averted per person and in the number of doses delivered in each strategy per person, we calculated the expected loss across a range of WTP thresholds ^44^. To do this, we used the {dampack} package ^43^, where for each parameter draw, we calculated the mean of the expected foregone benefits, or consequences, of a specific strategy relative to the optimal strategy for that parameter draw, across all draws. For example, for each strategy, we compared the net benefit $B_{i,s}$defined as:

$$B_{i,s}=\left( {Effect}_{i,s}*WTP \right)-{Cost}_{i,s}$$

where the effect ${Effect}_{i,s}$is clinical or severe cases averted per person, WTP is some threshold value of the number of doses per additional case averted that an entity is willing to deliver (ranging between 5 and 200 additional doses per case averted), and ${Cost}_{i,s}$ is the cost in terms of doses per person for a specific strategy $s$ and a parameter draw $i$. The net benefit of the optimal strategy, $B_{i,s_{i}^{*}}$, is defined as the maximum benefit across all strategies for each parameter draw $i$, where $s_{i}^{*}$ is the optimal strategy for the parameter draw $i$.

Expected loss $\bar{L}_{s}$ for each strategy $s$ is then defined as the difference in net benefit of the optimal strategy for draw $i$, $B_{i,s_{i}^{*}}$, and the net benefit of some suboptimal strategy, $B_{i,s}$, averaged over all parameter draws. Expected loss is in terms of additional doses per person.

$$\bar{L}_{s}=\frac{1}{N}\sum_{i=1}^{N} \left[ B_{i,s_{i}^{*}}-B_{i,s} \right]$$

Figure S15 shows the expected loss (additional doses per person) for each WTP threshold (doses per person required for that strategy per case averted, relative to routine-age based vaccination) across a range of perennial transmission intensities, along with the ranking of the 10 strategies with the lowest expected loss (1: lowest expected loss, 10: 10^th^ lowest expected loss). The strategy with the lowest expected loss is optimal at each WTP threshold, taking uncertainty into account.

For example, Figure S15 shows that, in a perennial setting with 5% *Pf*PR_2-10_, catch-up vaccination to children aged 6 months to 14 years plus two extra booster doses at 5 and 10 years has the lowest expected loss at every WTP threshold value (noted by the green dashed line in panel A and rank 1 in panel E). The second lowest expected loss was catch-up vaccination to children aged 6 months to 9 years plus two extra booster doses at 5 and 10 years, but only at the lowest value of WTP, whereas this strategy drops in rankings as the WTP threshold increases, following the line associated with this scenario in panel E from left to right, ending up as the scenario with the fifth lowest expected loss. The second lowest expected loss for all WTP thresholds, except when WTP was 5 doses per person required to avert one extra case relative to routine age-based vaccination, was a catch-up campaign to children aged 6 months to 14 years plus three extra booster doses at 2, 5, and 10 years. This indicates that regardless of the number of extra doses required by a strategy, a catch-up campaign to children aged 6 months to 14 years plus two extra booster doses at 5 and 10 years has the lowest expected loss.

In a higher transmission perennial setting, such as 45% *Pf*PR_2-10_ (Figure S15C and G), the most efficient strategy changes depending on the willingness to pay threshold. If a funder or country can contribute only 5 doses per case averted relative to routine age-based vaccination, then the strategy with the lowest expected loss is a catch-up campaign to children aged 6 months to 4 years plus two extra boosters at 5 and 10 years, whereas a catch-up campaign to children between 6 months and 14 years with two extra booster doses at 5 and 10 years has the lowest expected loss for WTP thresholds above 5 additional doses per case averted.

At low WTP thresholds, the rankings of the strategies were often different from the rankings at higher WTP thresholds. When the WTP threshold is low, strategies requiring fewer doses, even if they avert fewer cases, tend to be optimal because resources are constrained. For example, at 65% *Pf*PR_2-10_ in a perennial setting (Figure S15D and H), at a WTP threshold of 5 additional doses per case averted, the scenario with the lowest expected loss is two extra booster doses at 5 and 10 years, while the scenario with the lowest expected loss when the WTP threshold was between 15 and 25 additional doses per case averted is a catch-up campaign to children aged 6 months to 4 years combined with routine age-based vaccination with 3 extra booster doses (2y, 5y, 10y post primary series). However, for WTP thresholds above 25 additional doses per case averted, the strategy with the lowest expected loss is a scenario with a catch-up campaign to children aged 6 months to 9 years with the 3 extra booster doses. When WTP is higher, resources are less constrained, making the benefit in terms of clinical or severe cases averted more important than the number of doses used, so strategies averting more cases will tend to be optimal.

At lower transmission, even though it requires a lot of doses, a catch-up campaign targeting children aged 6 months to 14 years plus two extra booster doses (5y, 10y) at 5% *Pf*PR_2-10_ had a much lower expected loss across all values of WTP than the other strategies, reaching an expected loss of 0 at certain WTP values. This is likely because at low transmission, the burden of malaria is higher in school-aged children than in young children, so strategies targeting older children are optimal ^13^. The difference in expected loss between strategies is much higher at lower transmission than at high transmission when many of the strategies have similar expected loss. The strategy with the lowest expected loss for each transmission setting is always the one with the highest benefit and the highest cost on the efficiency frontier (top right of efficiency frontier).

***
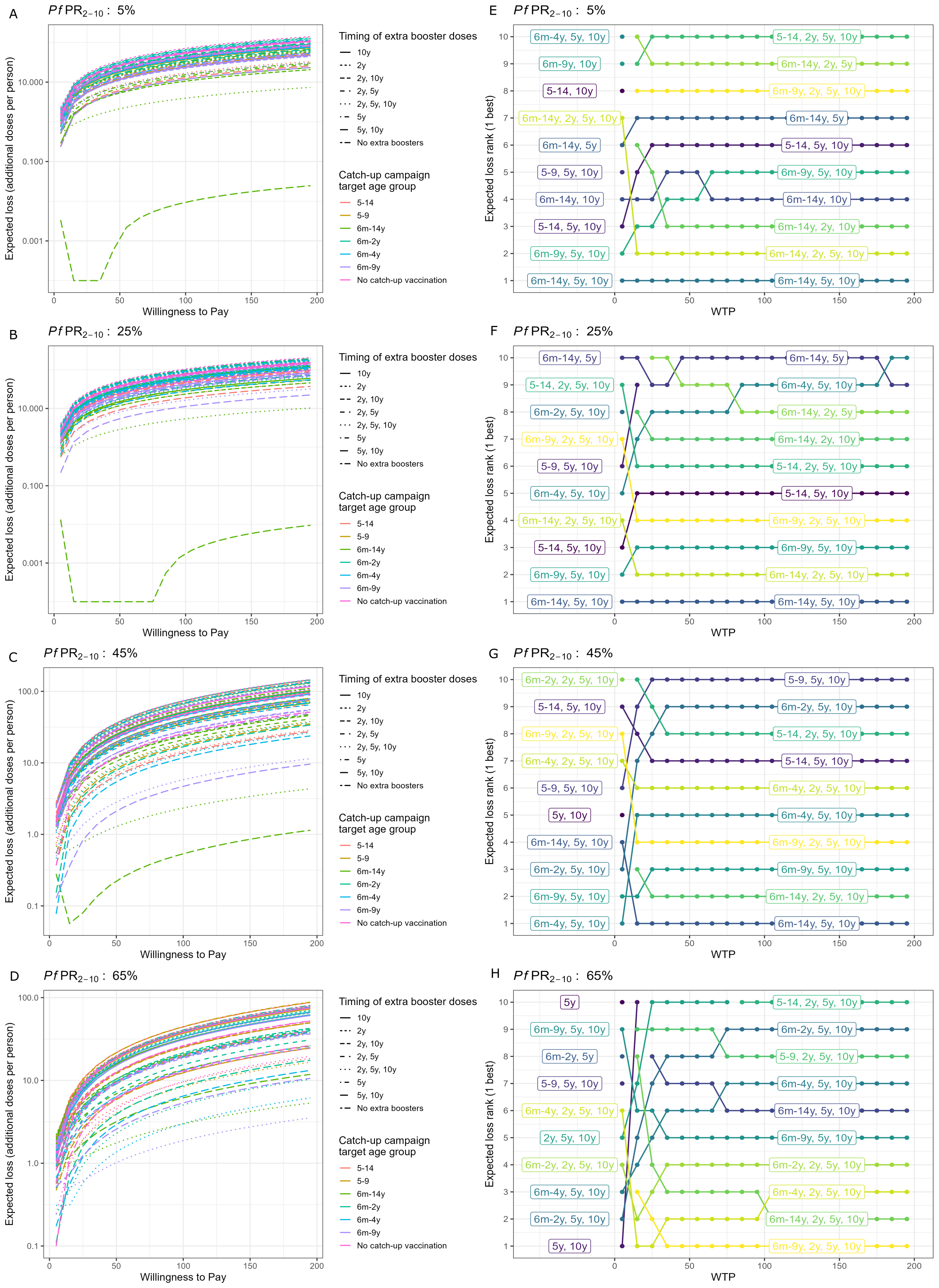
 Figure S15****. Expected loss curves of all modelled scenarios for a range of perennial transmission intensities (panels A-D) with their corresponding ranks from 1 (lowest expected loss) to 10 (10^th^ lowest expected loss) (panels E-H). Expected loss is in terms of additional doses per person, and willingness to pay is the number of doses per person required to avert 1000 cases.*
